# Classification of artificial intelligence tools for civil engineering under the notion of complex fuzzy rough Frank aggregation operators

**DOI:** 10.1038/s41598-024-60561-1

**Published:** 2024-05-24

**Authors:** Walid Emam, Jabbar Ahmmad, Tahir Mahmood, Ubaid ur Rehman, Shi Yin

**Affiliations:** 1 Department of Statistics and Operations Research, Faculty of Science, King Saud University, P.O. Box 2455, 11451 Riyadh, Saudi Arabia; 2 Department of Mathematics and Statistics, International Islamic University Islamabad, Islamabad, Pakistan; 3 College of Economics and Management, Hebei Agricultural University, Baoding, 071000 China; 4 Department of Robotics and AI, Szabist University, Islamabad, Pakistan

**Keywords:** Artificial Intelligence, Complex fuzzy rough set, Frank t-norm, t-conorm, Applied mathematics, Computer science, Pure mathematics

## Abstract

In recent days researchers have tried to handle the maximum information and use those techniques and methods in which there is no chance of data loss or loss of information is minimum. The structure like fuzzy set and complex fussy set cannot discuss the upper and lower approximations. Moreover, we can observe that a fuzzy rough set cannot discuss the second dimension and in this case, there is a chance of data loss. To cover all these issues in previous ideas, the notion of a complex fuzzy rough set in Cartesian form is the demand of the day because this structure can discuss the second dimension as well as upper and lower approximations. For this purpose, in this manuscript, we have developed the theory of complex fuzzy relation and complex fuzzy rough set in Cartesian form. Moreover, we have initiated the fundamental laws for complex fuzzy rough numbers based on Frank t-norm and t-conorm. The fundamental tools that can convert the overall input into a single output are called aggregation operators (AOs). So based on the characteristics of AOs, we have defined the notion of complex fuzzy rough Frank average and complex fuzzy rough Frank geometric AOs. The utilization of the developed theory is necessary to show the importance and validity of the delivered approach. So based on developed notions, we have defined an algorithm for this purpose along with an illustrative example. We have utilized the introduced structure for the classification of AI tools for civil engineering. Moreover, the comparative analysis of the delivered approach shows the advancement of the introduced structure as compared to existing notions.

## Introduction

### AI significance in civil engineering

Engineering has seen major advancements because of artificial intelligence (AI), which could completely transform several engineering-related fields. AI can help engineers create and improve complex systems. It can swiftly produce and assess design alternatives, assisting engineers in selecting the best options based on performance, cost, and other factors. AI-based computer vision systems can accurately inspect and find product flaws, ensuring quality control in production procedures. By examining previous project data to create more accurate forecasts regarding project timeframes and resource allocation, AI can help with project planning and scheduling. AI can be used to optimize how much energy is utilized in factories and buildings. Machine learning algorithms can adapt systems to reduce energy usage without compromising performance by learning from data. The use of AI in industrial robotics and automation is essential. AI may be used to train robots to carry out manufacturing, assembly, and construction jobs with greater efficiency and accuracy. Many researchers have analyzed the utilization of AI in the sector of civil engineering like Manzoor et al.^1^ study the influence of AI in civil engineering towards sustainable development. Reich^2^ in 1996 study the utilization of AI in bridge engineering. Also, Zhang et al.^3^ analyze the safety management of civil engineering construction based on AI and machine vision technology. Also, Reich^4^ studied the machine learning technique for civil engineering problems. Moreover, Zhang et al.^5^ initiated genetic programming in civil engineering and they have also proposed its application and studied the future trends. Furthermore, Harandizadeh and Toufigh^6^ proposed the application of newly developed AI approaches in civil engineering for ultimate pile-bearing capacity prediction in soil based on experimental datasets. Moreover, Shuford^7^ explored the impact of deep reinforcement learning on enhancing the decision-making capabilities of AI systems. As frank operators have their importance, Wang et al.^8^ proposed the notion of Frank AOs.

### Literature review

The notion of fuzzy set (FS)^9^ provides a mathematical framework that connects the qualitative, unpredictability of human cognition with the demand for formal, computational procedures in diverse domains. FS and fuzzy logic provide a potent tool for dealing with and controlling uncertainty, ambiguity, and imprecision in a variety of applications. Information is not always exact or binary (true/false) in many real-world applications. Information that is uncertain or imprecise can be represented and used in FSs. For instance, it would be more accurate to describe the concept of "partly cloudy" as "fuzzy" rather than using the clear-cut crisp categories of "cloudy" or "not cloudy." The uncertainty in human language and perception is better captured by FSs. Traditional set theory is challenging to apply to natural language since it is frequently ambiguous and context-dependent. These linguistic words can be represented in a quantitative way using FSs. Different granularities of information can be modeled using FSs. FSs can represent a continuous range of heights that move gradually from one category to another, representing the reality that there is no sharp distinction between "tall" and "short," for instance, when defining a person's height as "tall" or "short." The notion of FS uses the membership grade (MG) whose range belongs to [0, 1]. FS has many applications in civil engineering. Chameau et al.^10^ utilize the idea of FS and they have proposed the potential application of FS in civil engineering. Chan et al.^11^ provide a study of the application of fuzzy techniques in construction management research. Moreover, Ayyub and Haldar^12^ study project scheduling using the FS concepts. Lorterapong and Moselhi^13^ study the project network analysis using the idea of FS. Guan et al.^14^ have used the notion of complex linear diophantine FS over AG-groupoids with application in civil engineering. Gurcanli and Mungen^15^ study an occupational safety risk analysis method at construction sites using the concept of FS. Gong et al.^16^ analyze the geotechnical design of earth slopes using the notion of FS. Also, Narayanamoorthy et al.^17^ utilize a novel augmented Fermatean MCDM approach for the identification of renewable energy power plant locations. In many decision-making situations, we have discussed the second dimension of a variable. So we need to discuss such a kind of fuzzy structure that handles such kind of information. FS has its drawback that it can never consider the second dimension of a variable. So the notion of complex fuzzy set (CFS) has been initiated. Two kinds of attempts have been made in this regard, one by Ramot et al.^18^ and a second by Tamir et al.^19^. In the first structure developed by Ramot et al.^18^, they used the range of MG belonging to the unit circle in a complex plane. But Tamir et al.^19^ generalized this theory and used this range belonging to unit squares instead of the unit circle in the complex plane. CFS is a more generalized form than FS due to considering the second dimension (extra fuzzy information) of a variable. CFS enhances the range of data and there is less chance of data loss in this structure, whenever decision-makers want to take their data in the form of CFS. The literature is rich with the application of CFS in different sectors. Ma et al.^20^ proposed a method for multiple periodic factor prediction problems using CFS. Bi et al.^21^ used the notion of CFS and proposed the concept of CF arithmetic AOs. Hu et al.^22^ establish the orthogonality relation of CFS. Chen et al.^23^ established the adaptive neuro-complex fuzzy inferential system and applied this system to the domain of time series forecasting. Bi et al.^24^ proposed two classes of similarity measures for CFSs. Moreover, Tuncer et al.^25^ developed a discrete fuzzy transform-based face image recognition method.

Rough set (RS)^26^ theory is motivated by its capacity to deal with data uncertainty and imprecision, offers a systematic method for feature selection and reduction, helps decision-making processes, produces comprehensible results, and promotes knowledge discovery across a variety of fields. It has found use in a variety of domains, including knowledge representation and decision support systems as well as data analysis and machine learning. Many new developments have been designed based on RS and FS theory like the idea of the fuzzy rough set (FRS)^27^, intuitionistic fuzzy rough set (IFRS)^28^, Pythagorean fuzzy rough set (PyFRS)^29^, and q-ring orthopair fuzzy rough set (q-ROFRS)^30^. All the above theories have their applications in different fields. Tang et al.^31^ developed the topological structures of FRSs. Sharma et al.^32^ introduced similarity and distance measures based on FRS and proposed their applications. Zhou et al.^33^ study the characterization of IFRS based on IF implicators. Zhang^34^ developed the classification rule mining algorithm by using the notion of IFRS and genetic algorithm. Liu et al.^35^ discuss the decision support methodology based on covering-based interval-valued PyFRS and its application in hospital open-source. Moreover, Akram and Zahid^36^ developed the group decision-making method with PyFR numbers for the evaluation of the best design concept. Qahtan et al.^37^ study the performance assessment of sustainable transportation in the shipping industry by using the notion of q-ROFRSs. Khoshaim et al.^38^ proposed emergency decision-making based on q-ROFR AOs.

### Motivation of the research study

The use of the generalized structure in fuzzy set theory is currently necessary, and numerous developments have been made in this direction. According to the literature, there are still limitations that are discussed as follows.

The idea of FS falls short when dealing with information of this nature when the second dimension is discussed in a single structure. Take note that Ramot et al.^18^ concept of a complex fuzzy set uses the range of MG as a unit circle in the complex plane, and the idea of CFS proposed by Tamir et al.^19^ uses the range of MG belonging to the unit square in the complex plane. However, we can notice that both of these structures lack the property of considering the upper and lower approximations. So there is a chance of data loss in all these structures.

### The main contribution of this study

**Figure 1:**
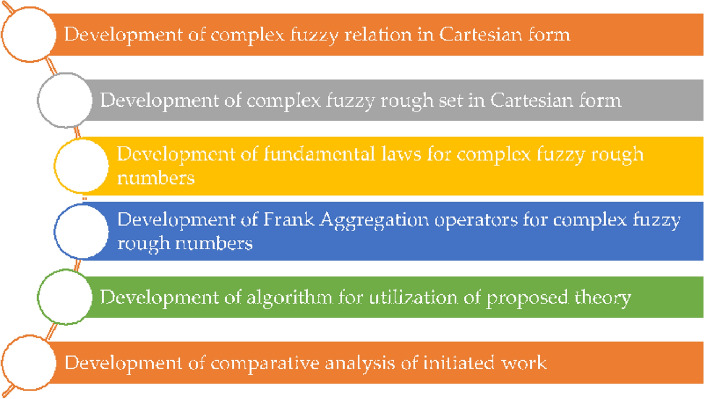
Graphical representation of the established theory.

Based on the observations of the literature given in the above section, it is necessary for the literature to discuss notions that can handle the upper and lower approximation and can also discuss the second dimension in one structure. Moreover, the range of that structure must be a unit square. So, the idea of a complex fuzzy rough set in Cartesian form is in high demand right now because it can discuss both upper and lower approximations as well as the second dimension, which can be taken into account by some already existing structures like FS and CFS. So in this article, the theory of complex fuzzy relation and complex fuzzy rough set in Cartesian form has been developed. Moreover, we have initiated the fundamental laws for complex fuzzy rough numbers based on Frank t-norm and t-conorm. The fundamental tools that can convert the overall input into a single output are called AOs. Thus, we defined the concepts of complex fuzzy rough Frank average and complex fuzzy rough Frank geometric AOs. The utilization of the developed theory is necessary to show the importance and validity of the delivered approach. So based on developed notions, we have defined an algorithm for this purpose along with an illustrative example. We have utilized the introduced structure for the classification of AI tools for civil engineering. Moreover, the comparative analysis of the delivered approach shows the advancement of the introduced structure as compared to existing notions. The graphical representation of the proposed work is given in Fig. 1.

### Study structure

The remainder of the paper is organized as follows. We have revised the definitions of RS, FS, CFS, and frank t-norm and t-conorm in “Preliminaries”. The concept of a complex fuzzy rough set in Cartesian form and a complex fuzzy relation is covered in “Construction of complex fuzzy rough set (CFRS) in Cartesian form”. Furthermore, the basic operating rules for complex fuzzy rough numbers are also discussed in “Construction of complex fuzzy rough set (CFRS) in Cartesian form”. The complex fuzzy rough frank average and geometric AOs are covered in “Complex fuzzy rough Frank (CFRF) aggregation operators (AOs)” and “Complex fuzzy rough Frank geometric (CFRFG) AOs”. In “MADM Approach for the developed notions”, the introduced work's application algorithm and numerical examples are discussed. The comparative analysis is developed in “Comparative analysis”. “Conclusion” discusses the developed theory's conclusion. “Abbreviations” is for abbreviations.

## Preliminaries

In this section, we will review the basic definitions of RS, FS, CFS, and Frank t-norm and t-conorm.

### Definition 1

^26^ Let $\rotatebox{180}{{\rm h}}$ be the universal set (US) and ${R}_{el.}$ represent an equivalence relation, the pair $(\rotatebox{180}{{\rm h}}, {R}_{el.})$ is referred to as an approximation space. For a non-empty set $A\subset \rotatebox{180}{{\rm h}}$, if the set $A$ can be expressed as the union of some equivalency classes then $A$ is said to be definable. Otherwise, it is not definable. Now, if $A$ is not definable then we can approximate the set $A$ into definable subsets referred to as lower and upper approximations given by

$$\underline{{R}_{el.}}\left(A\right)=\left\{\left(a\in \rotatebox{180}{{\rm h}}:{\left[a\right]}_{{R}_{el.}}\subseteq A\right)\right\}$$

$$\overline{{R}_{el.}}\left(A\right)=\left\{\left(a\in \rotatebox{180}{{\rm h}}: {\left[a\right]}_{{R}_{el.}}\cap A\ne \varnothing \right)\right\}$$

The pair $\left(\underline{{R}_{el.}}\left(A\right), \overline{{R}_{el.}}\left(A\right)\right)$ is called RS, where $\underline{{R}_{el.}}\left(A\right)\ne \overline{{R}_{el.}}\left(A\right)$

### Example 1

Let $\rotatebox{180}{{\rm h}}=\{{\rotatebox{180}{{\rm h}}}_{1}, {\rotatebox{180}{{\rm h}}}_{2}, \dots , {\rotatebox{180}{{\rm h}}}_{5}\}$ and $${R}_{el.}=\left\{\begin{array}{c}({\rotatebox{180}{{\rm h}}}_{1}, {\rotatebox{180}{{\rm h}}}_{1}), ({\rotatebox{180}{{\rm h}}}_{2}, {\rotatebox{180}{{\rm h}}}_{2}), ({\rotatebox{180}{{\rm h}}}_{3}, {\rotatebox{180}{{\rm h}}}_{3}), ({\rotatebox{180}{{\rm h}}}_{4}, {\rotatebox{180}{{\rm h}}}_{4}), ({\rotatebox{180}{{\rm h}}}_{5}, {\rotatebox{180}{{\rm h}}}_{5}),\\ ({\rotatebox{180}{{\rm h}}}_{1}, {\rotatebox{180}{{\rm h}}}_{3}), ({\rotatebox{180}{{\rm h}}}_{3}, {\rotatebox{180}{{\rm h}}}_{1}), ({\rotatebox{180}{{\rm h}}}_{1}, {\rotatebox{180}{{\rm h}}}_{5}), ({\rotatebox{180}{{\rm h}}}_{5}, {\rotatebox{180}{{\rm h}}}_{1})\end{array}\right\}.$$ Then ${R}_{el.}$ is an equivalence relation on $\rotatebox{180}{{\rm h}}$. Now equivalence classes are given by

$${[{\rotatebox{180}{{\rm h}}}_{1}]}_{{R}_{el.}}=\{{\rotatebox{180}{{\rm h}}}_{1}, {\rotatebox{180}{{\rm h}}}_{3}, {\rotatebox{180}{{\rm h}}}_{5}\}, {[{\rotatebox{180}{{\rm h}}}_{2}]}_{{R}_{el.}}=\{{\rotatebox{180}{{\rm h}}}_{2}\}, {[{\rotatebox{180}{{\rm h}}}_{3}]}_{{R}_{el.}}=\{{\rotatebox{180}{{\rm h}}}_{1}, {\rotatebox{180}{{\rm h}}}_{3}\}, {[{\rotatebox{180}{{\rm h}}}_{4}]}_{{R}_{el.}}=\{{\rotatebox{180}{{\rm h}}}_{4}\}$$ and $${[{\rotatebox{180}{{\rm h}}}_{5}]}_{{R}_{el.}}=\{{\rotatebox{180}{{\rm h}}}_{1}, {\rotatebox{180}{{\rm h}}}_{5}\}$$.

Now let $A=\{{\rotatebox{180}{{\rm h}}}_{2}, {\rotatebox{180}{{\rm h}}}_{3},{\rotatebox{180}{{\rm h}}}_{4}, {\rotatebox{180}{{\rm h}}}_{5}\}$ then

$$\underline{{R}_{el.}}(A)=\{{\rotatebox{180}{{\rm h}}}_{2}, {\rotatebox{180}{{\rm h}}}_{4}\}$$ and $$\overline{{R}_{el.}}(A)=\{{\rotatebox{180}{{\rm h}}}_{1}, {\rotatebox{180}{{\rm h}}}_{2}, {\rotatebox{180}{{\rm h}}}_{3}, {\rotatebox{180}{{\rm h}}}_{4}, {\rotatebox{180}{{\rm h}}}_{5}\}.$$

As $\underline{{R}_{el.}}(A)\ne \overline{{R}_{el.}}(A)$ then ${R}_{el.}(A)=(\underline{{R}_{el.}}(A), \overline{{R}_{el.}}(A))$ is called a rough set.

### Definition 2

^8^ The structure of FS has the form

$$FS=\left\{\left(x, \mathfrak{L}\left(x\right)\right)| 0\le \mathfrak{L}\left(x\right)\le 1\right\}$$

Here $\mathfrak{L}\left(x\right)$ represent the MG and $\mathfrak{L}:\rotatebox{180}{{\rm h}}\to \left[0, 1\right].$  

### Definition 3

^19^ For a US $\rotatebox{180}{{\rm h}},$ the notion of CFS is given by

$$CFS=\left\{x, \mathfrak{L}\left(x\right)+i \frown\!\!\!\!\!\! {\smallint} \left(x\right)| 0\le \mathfrak{L}\left(x\right)\le 1\,\, {\text{and}}\,\, 0\le \frown\!\!\!\!\!\! {\smallint} \left(x\right)\le 1\right\}$$

where $\mathfrak{L}\left(x\right)$ represent the real part of the MG and $\mathfrak{L}:\rotatebox{180}{{\rm h}}\to \left[0, 1\right]$ and $\frown\!\!\!\!\!\! {\smallint} \left(x\right)$ represent imaginary part of the MG with $\frown\!\!\!\!\!\! {\smallint} \rotatebox{180}{{\rm h}}\to \left[0, 1\right]$.

### Definition 4

^8^ The notion of Frank t-norm and Frank t-conorm is given as

1. $$x\oplus y=\left(1-{{\text{log}}}_{{{{\sf (} \! \rotatebox{146}{\sf c}}}^{\divideontimes }}\left(1+\frac{\left({{{{\sf (} \! \rotatebox{146}{\sf c}}}^{\divideontimes }}^{1-x}-1\right)\left({{{{\sf (} \! \rotatebox{146}{\sf c}}}^{\divideontimes }}^{1-{\text{y}}}-1\right)}{{{{\sf (} \! \rotatebox{146}{\sf c}}}^{\divideontimes }-1}\right)\right)$$ for all $$\left(x, y\right)\in \left[0, 1\right]\times \left[0, 1\right]$$
2. $$x \otimes y=\left({{\text{log}}}_{{{{\sf (} \! \rotatebox{146}{\sf c}}}^{\divideontimes }}\left(1+\frac{\left({{{{\sf (} \! \rotatebox{146}{\sf c}}}^{\divideontimes }}^{1-x}-1\right)\left({{{{\sf (} \! \rotatebox{146}{\sf c}}}^{\divideontimes }}^{1-{\text{y}}}-1\right)}{{{{\sf (} \! \rotatebox{146}{\sf c}}}^{\divideontimes }-1}\right)\right)$$ for all $$\left(x, y\right)\in \left[0, 1\right]\times \left[0, 1\right]$$

## Construction of complex fuzzy rough set (CFRS) in Cartesian form

To define the notion of a complex fuzzy rough set in Cartesian form has its importance and there is no such idea has been developed. For this purpose, the importance of complex fuzzy relations in Cartesian form cannot be denied. So in this part of the article, first of all, we have established the idea of complex fuzzy relation and then based on this relation we have delivered the idea of CFRS in Cartesian form. The overall discussion is given by:

### Definition 5

For any two sets $P$ and $Q$, the structure ${R}_{el.}=\left\{\left({\mathbbm{p}},{\mathbbm{q}}\right),\mathfrak{M}\left({\mathbbm{p}},{\mathbbm{q}}\right)|\mathfrak{M}\left({\mathbbm{p}},{\mathbbm{q}}\right)=\mathcal{S}\left({\mathbbm{p}},{\mathbbm{q}}\right)+\mathfrak{i}\mathcal{T}\left({\mathbbm{p}},{\mathbbm{q}}\right)\, {\text{where}}\, {\mathbbm{p}}\in P\, {\text{and}}\, {\mathbbm{q}}\in Q\right\}$ that is the complex fuzzy subset of $P\times Q,$ where $0\le \mathcal{S}\left({\mathbbm{p}},{\mathbbm{q}}\right)\le 1, 0\le \mathcal{T}\left({\mathbbm{p}},{\mathbbm{q}}\right)\le 1$ and $\mathfrak{M}\left({\mathbbm{p}},{\mathbbm{q}}\right):P\times Q\to \left[0, 1\right]+\mathfrak{i}\left[0, 1\right]$, is called complex fuzzy relation (CFR) from $P$ to $Q$.

### Definition 6

For any arbitrary set $P$ the structure of the form ${R}_{el.}=\left\{\left({\mathbbm{p}},{\mathbbm{q}}\right),\mathfrak{M}\left({\mathbbm{p}},{\mathbbm{q}}\right)|\mathfrak{M}\left({\mathbbm{p}},{\mathbbm{q}}\right)=\mathcal{S}\left({\mathbbm{p}},{\mathbbm{q}}\right)+\mathfrak{i}\mathcal{T}\left({\mathbbm{p}},{\mathbbm{q}}\right)\, where\, P, {\mathbbm{q}}\in P \right\}$ that is the complex fuzzy subset $"{R}_{el.}"$ of $P\times P,$ where $0\le \mathcal{S}\left({\mathbbm{p}},{\mathbbm{q}}\right)\le 1, 0\le \mathcal{T}\left({\mathbbm{p}},{\mathbbm{q}}\right)\le 1$ and $\mathfrak{M}\left({\mathbbm{p}},{\mathbbm{q}}\right):P\times Q\to \left[0, 1\right]+\mathfrak{i}\left[0, 1\right]$ is called CFR on $P$.

### Definition 7

Assume that ${R}_{el.}$ be a CFR on $P$ then $\left(P, {R}_{el.}\right)$ define the complex fuzzy approximation space (CFAS). Now for any set ${\mathcal{A}}\in CFS\left(P\right),{\mathcal{A}}=\left\{{\mathcalligra{a}}\left({\mathbbm{p}}\right)+\iota {\mathcalligra{b}}\left({\mathbbm{p}}\right)\right\}$ then upper and lower approximations of $A$ concerning $\left(P, {R}_{el.}\right)$ is defined as follows

$${R}_{el.}^{{\rotatebox{180}{{\rm h}}}^{\diamond }}\left(A\right)=\left\{\left({\mathbbm{p}},{\mathfrak{O}}_{{R}_{el.}^{{\rotatebox{180}{{\rm h}}}^{\diamond }}}\left({\mathbbm{p}}\right)\right)|{\mathbbm{p}}\in P\right\}$$

$${R}_{el.}^{{ { \bar{\d{\rm L}}} }^{\diamond }}\left(A\right)=\left\{\left({\mathbbm{p}},{\mathfrak{O}}_{{R}_{el.}^{{ { \bar{\d{\rm L}}} }^{\diamond }}}\left({\mathbbm{p}}\right)\right)|{\mathbbm{p}}\in P\right\}$$

where

$${\mathfrak{O}}_{{R}_{el.}^{{\rotatebox{180}{{\rm h}}}^{\diamond }}}\left({\mathbbm{p}}\right)={\bigvee }_{{\mathbbm{q}}\in P}\left[\mathcal{S}\left({\mathbbm{p}},{\mathbbm{q}}\right)\bigwedge {\mathcalligra{a}}\left({\mathbbm{q}}\right)\right]+\mathfrak{i}{\bigvee }_{{\mathbbm{p}}\in P}\left[\mathcal{T}\left({\mathbbm{p}},{\mathbbm{q}}\right)\bigwedge {\mathcalligra{b}}\left({\mathbbm{q}}\right)\right]$$

$${\mathfrak{O}}_{{R}_{el.}^{{{{ { \bar{\d{\rm L}}} }}}^{\diamond }}}\left({\mathbbm{p}}\right)={\bigwedge }_{{\mathbb{p}}\in P}\left[1-\mathcal{S}\left({\mathbb{p}},{\mathbb{q}}\right)\bigvee \mathcal{a}\left({\mathbb{q}}\right)\right]+\mathfrak{i}{\bigwedge }_{{\mathbb{p}}\in P}\left[1-\mathcal{T}\left({\mathbb{p}},{\mathbb{q}}\right)\bigvee \mathcal{b}\left({\mathbb{q}}\right)\right]$$

The pair $\left({R}_{el.}^{{\rotatebox{180}{{\rm h}}}^{\diamond }}\left(A\right), {R}_{el.}^{{ { \bar{\d{\rm L}}} }^{\diamond }}\left(A\right)\right)$ is called complex fuzzy rough set (CFRS) with respect $\left(P, {R}_{el.}\right)$ if ${R}_{el.}^{{\rotatebox{180}{{\rm h}}}^{\diamond }}\left(A\right)\ne {R}_{el.}^{{ { \bar{\d{\rm L}}} }^{\diamond }}\left(A\right).$ For simplicity, we will say that ${\hat{C} }_{om.}=\left({\mathfrak{L}}^{{\rotatebox{180}{{\rm h}}}^{\diamond }}+\iota { \frown\!\!\!\!\!\! {\smallint} }^{{\rotatebox{180}{{\rm h}}}^{\diamond }}, {\mathfrak{L}}^{{ { \bar{\d{\rm L}}} }^{\diamond }}+\iota { \frown\!\!\!\!\!\! {\smallint} }^{{ { \bar{\d{\rm L}}} }^{\diamond }}\right)$ represent the CFRN.

### Example 2

Let $P=\left\{{\mathbbm{p}}_{1}, {\mathbbm{p}}_{2},{\mathbbm{p}}_{3}, {\mathbbm{p}}_{4}, {\mathbbm{p}}_{5}, {\mathbbm{p}}_{6}\right\}$ be a universal set. So CFR is given in Table 1 as follows.

**Table 1:** CFR.

${\varvec{R}}$	${\mathbbm{p}}_{1}$	${\mathbbm{p}}_{2}$	${\mathbbm{p}}_{3}$	${\mathbbm{p}}_{4}$	${\mathbbm{p}}_{5}$
${\mathbbm{p}}_{1}$	$0.50+\mathfrak{i}0.24$	$0.16+\mathfrak{i}0.11$	$0.17+\mathfrak{i}0.32$	$0.13+\mathfrak{i}0.26$	$0.26+\mathfrak{i}0.19$
${\mathbbm{p}}_{2}$	$0.21+\mathfrak{i}0.13$	$0.12+\mathfrak{i}0.42$	$0.35+\mathfrak{i}0.41$	$0.47+\mathfrak{i}0.23$	$0.15+\mathfrak{i}0.33$
${\mathbbm{p}}_{3}$	$0.21+\mathfrak{i}0.14$	$0.15+\mathfrak{i}0.44$	$0.14+\mathfrak{i}0.53$	$0.17+\mathfrak{i}0.42$	$0.16+\mathfrak{i}0.23$
${\mathbbm{p}}_{4}$	$0.15+\mathfrak{i}0.14$	$0.45+\mathfrak{i}0.48$	$0.61+\mathfrak{i}0.16$	$0.18+\mathfrak{i}0.27$	$0.32+\mathfrak{i}0.27$
${\mathbbm{p}}_{5}$	$0.19+\mathfrak{i}0.13$	$0.16+\mathfrak{i}0.13$	$0.21+\mathfrak{i}0.39$	$0.39+\mathfrak{i}0.28$	$0.41+\mathfrak{i}0.27$

Now we assume that $A=\left\{\left({\mathbbm{p}}_{1}, 0.23+\mathfrak{i}0.15\right), \left({\mathbbm{p}}_{2}, 0.18+\mathfrak{i}0.16\right), \left({\mathbbm{p}}_{3}, 0.14+\mathfrak{i}0.26\right), \left({\mathbbm{p}}_{4}, 0.17+\mathfrak{i}0.18\right), \left({\mathbbm{p}}_{5}, 0.19+\mathfrak{i}0.44\right)\right\}$ be a CFS, then upper and lower approximations are given by

$${\mathfrak{O}}_{{R}_{el.}^{{\rotatebox{180}{{\rm h}}}^{\diamond }}}\left({\mathbbm{p}}\right)=\left\{\begin{array}{c}\left({\mathbbm{p}}_{1}, 0.23+\mathfrak{i}0.26\right), \left({\mathbbm{p}}_{2}, 0.21+\mathfrak{i}0.33\right), \left({\mathbbm{p}}_{3}, 0.21+\mathfrak{i}0.26\right),\\ \left({\mathbbm{p}}_{4}, 0.19+\mathfrak{i}0.27\right), \left({\mathbbm{p}}_{5}, 0.19+\mathfrak{i}0.27\right)\end{array}\right\}$$

$${\mathfrak{O}}_{{R}_{el.}^{{ { \bar{\d{\rm L}}} }^{\diamond }}}\left({\mathbbm{p}}\right)=\left\{\begin{array}{c}\left({\mathbbm{p}}_{1}, 0.5+\mathfrak{i}0.68\right), \left({\mathbbm{p}}_{2}, 0.53+\mathfrak{i}0.58\right), \left({\mathbbm{p}}_{3}, 0.79+\mathfrak{i}0.47\right),\\ \left({\mathbbm{p}}_{4}, 0.39+\mathfrak{i}0.52\right), \left({\mathbbm{p}}_{5}, 0.61+\mathfrak{i}0.61\right)\end{array}\right\}$$

Hence it is seen that

$$\left({\mathfrak{O}}_{{R}_{el.}^{{\rotatebox{180}{{\rm h}}}^{\diamond }}}\left({\mathbbm{p}}\right), {\mathfrak{O}}_{{R}_{el.}^{{ { \bar{\d{\rm L}}} }^{\diamond }}}\left({\mathbbm{p}}\right)\right)=\left(\begin{array}{c}\left\{\begin{array}{c}\left({\mathbbm{p}}_{1}, 0.23+\mathfrak{i}0.26\right), \left({\mathbbm{p}}_{2}, 0.21+\mathfrak{i}0.33\right), \left({\mathbbm{p}}_{3}, 0.21+\mathfrak{i}0.26\right),\\ \left({\mathbbm{p}}_{4}, 0.19+\mathfrak{i}0.27\right), \left({\mathbbm{p}}_{5}, 0.19+\mathfrak{i}0.27\right) \end{array}\right\}, \\ \left\{\begin{array}{c}\left({\mathbbm{p}}_{1}, 0.5+\mathfrak{i}0.68\right), \left({\mathbbm{p}}_{2}, 0.53+\mathfrak{i}0.58\right), \left({\mathbbm{p}}_{3}, 0.79+\mathfrak{i}0.47\right),\\ \left({\mathbbm{p}}_{4}, 0.39+\mathfrak{i}0.52\right), \left({\mathbbm{p}}_{5}, 0.61+\mathfrak{i}0.61\right)\end{array}\right\}\end{array}\right)$$ is a CFRS because ${\mathfrak{O}}_{{R}_{el.}^{{\rotatebox{180}{{\rm h}}}^{\diamond }}}\left({\mathbbm{p}}\right)\ne {\mathfrak{O}}_{{R}_{el.}^{{ { \bar{\d{\rm L}}} }^{\diamond }}}\left({\mathbbm{p}}\right).$

### Operational rules for CFRNs based on Frank t-norm and t-conorm

This section is devoted to proposing some elementary operational rules based on Frank t-norm and t-conorm for CFRNs.

#### Definition 8

Assume that ${\hat{C} }_{om.-1}=\left({\mathfrak{L}}_{1}^{{\rotatebox{180}{{\rm h}}}^{\diamond }}+\iota { \frown\!\!\!\!\!\! {\smallint} }_{1}^{{\rotatebox{180}{{\rm h}}}^{\diamond }}, {\mathfrak{L}}_{1}^{{ { \bar{\d{\rm L}}} }^{\diamond }}+\iota { \frown\!\!\!\!\!\! {\smallint} }_{1}^{{ { \bar{\d{\rm L}}} }^{\diamond }}\right)$ and ${\hat{C} }_{om.-2}=\left({\mathfrak{L}}_{2}^{{\rotatebox{180}{{\rm h}}}^{\diamond }}+\iota { \frown\!\!\!\!\!\! {\smallint} }_{2}^{{\rotatebox{180}{{\rm h}}}^{\diamond }}, {\mathfrak{L}}_{2}^{{ { \bar{\d{\rm L}}} }^{\diamond }}+\iota { \frown\!\!\!\!\!\! {\smallint} }_{2}^{{ { \bar{\d{\rm L}}} }^{\diamond }}\right)$ be two CFRNs, ${{{\sf (} \! \rotatebox{146}{\sf c}}}^{\divideontimes }>1$, and ${\mathfrak{t}}^{\ddagger }>0$ is any real number. Then the operational rules based on Frank t-norm and t-conorm for CFRNs are given by

1. $$\begin{aligned} & {\hat{C} }_{om.-1}\oplus {\hat{C} }_{om.-2}\\ & \quad =\left(\begin{array}{c}1-{{\text{log}}}_{{{{\sf (} \! \rotatebox{146}{\sf c}}}^{\divideontimes }}\left(1+\frac{\left({{{{\sf (} \! \rotatebox{146}{\sf c}}}^{\divideontimes }}^{1-{\mathfrak{L}}_{1}^{{\rotatebox{180}{{\rm h}}}^{\diamond }}}-1\right)\left({{{{\sf (} \! \rotatebox{146}{\sf c}}}^{\divideontimes }}^{1-{\mathfrak{L}}_{2}^{{\rotatebox{180}{{\rm h}}}^{\diamond }}}-1\right)}{{{{\sf (} \! \rotatebox{146}{\sf c}}}^{\divideontimes }-1}\right)+\iota 1-{{\text{log}}}_{{{{\sf (} \! \rotatebox{146}{\sf c}}}^{\divideontimes }}\left(1+\frac{\left({{{{\sf (} \! \rotatebox{146}{\sf c}}}^{\divideontimes }}^{1-{ \frown\!\!\!\!\!\! {\smallint} }_{1}^{{\rotatebox{180}{{\rm h}}}^{\diamond }}}-1\right)\left({{{{\sf (} \! \rotatebox{146}{\sf c}}}^{\divideontimes }}^{1-{ \frown\!\!\!\!\!\! {\smallint} }_{2}^{{\rotatebox{180}{{\rm h}}}^{\diamond }}}-1\right)}{{{{\sf (} \! \rotatebox{146}{\sf c}}}^{\divideontimes }-1}\right),\\ 1-{{\text{log}}}_{{{{\sf (} \! \rotatebox{146}{\sf c}}}^{\divideontimes }}\left(1+\frac{\left({{{{\sf (} \! \rotatebox{146}{\sf c}}}^{\divideontimes }}^{1-{\mathfrak{L}}_{1}^{{ { \bar{\d{\rm L}}} }^{\diamond }}}-1\right)\left({{{{\sf (} \! \rotatebox{146}{\sf c}}}^{\divideontimes }}^{1-{\mathfrak{L}}_{2}^{{ { \bar{\d{\rm L}}} }^{\diamond }}}-1\right)}{{{{\sf (} \! \rotatebox{146}{\sf c}}}^{\divideontimes }-1}\right)+\iota 1-{{\text{log}}}_{{{{\sf (} \! \rotatebox{146}{\sf c}}}^{\divideontimes }}\left(1+\frac{\left({{{{\sf (} \! \rotatebox{146}{\sf c}}}^{\divideontimes }}^{1-{ \frown\!\!\!\!\!\! {\smallint} }_{1}^{{ { \bar{\d{\rm L}}} }^{\diamond }}}-1\right)\left({{{{\sf (} \! \rotatebox{146}{\sf c}}}^{\divideontimes }}^{1-{ \frown\!\!\!\!\!\! {\smallint} }_{2}^{{ { \bar{\d{\rm L}}} }^{\diamond }}}-1\right)}{{{{\sf (} \! \rotatebox{146}{\sf c}}}^{\divideontimes }-1}\right)\end{array}\right)\end{aligned}$$
2. $${\hat{C} }_{om.-1}\otimes {\hat{C} }_{om.-2}=\left(\begin{array}{c}{{\text{log}}}_{{{{\sf (} \! \rotatebox{146}{\sf c}}}^{\divideontimes }}\left(1+\frac{\left({{{{\sf (} \! \rotatebox{146}{\sf c}}}^{\divideontimes }}^{{\mathfrak{L}}_{1}^{{\rotatebox{180}{{\rm h}}}^{\diamond }}}-1\right)\left({{{{\sf (} \! \rotatebox{146}{\sf c}}}^{\divideontimes }}^{{\mathfrak{L}}_{2}^{{\rotatebox{180}{{\rm h}}}^{\diamond }}}-1\right)}{{{{\sf (} \! \rotatebox{146}{\sf c}}}^{\divideontimes }-1}\right)+\iota {{\text{log}}}_{{{{\sf (} \! \rotatebox{146}{\sf c}}}^{\divideontimes }}\left(1+\frac{\left({{{{\sf (} \! \rotatebox{146}{\sf c}}}^{\divideontimes }}^{{ \frown\!\!\!\!\!\! {\smallint} }_{1}^{{\rotatebox{180}{{\rm h}}}^{\diamond }}}-1\right)\left({{{{\sf (} \! \rotatebox{146}{\sf c}}}^{\divideontimes }}^{{ \frown\!\!\!\!\!\! {\smallint} }_{2}^{{\rotatebox{180}{{\rm h}}}^{\diamond }}}-1\right)}{{{{\sf (} \! \rotatebox{146}{\sf c}}}^{\divideontimes }-1}\right),\\ {{\text{log}}}_{{{{\sf (} \! \rotatebox{146}{\sf c}}}^{\divideontimes }}\left(1+\frac{\left({{{{\sf (} \! \rotatebox{146}{\sf c}}}^{\divideontimes }}^{{\mathfrak{L}}_{1}^{{ { \bar{\d{\rm L}}} }^{\diamond }}}-1\right)\left({{{{\sf (} \! \rotatebox{146}{\sf c}}}^{\divideontimes }}^{{\mathfrak{L}}_{2}^{{ { \bar{\d{\rm L}}} }^{\diamond }}}-1\right)}{{{{\sf (} \! \rotatebox{146}{\sf c}}}^{\divideontimes }-1}\right)+\iota {{\text{log}}}_{{{{\sf (} \! \rotatebox{146}{\sf c}}}^{\divideontimes }}\left(1+\frac{\left({{{{\sf (} \! \rotatebox{146}{\sf c}}}^{\divideontimes }}^{{ \frown\!\!\!\!\!\! {\smallint} }_{1}^{{ { \bar{\d{\rm L}}} }^{\diamond }}}-1\right)\left({{{{\sf (} \! \rotatebox{146}{\sf c}}}^{\divideontimes }}^{{ \frown\!\!\!\!\!\! {\smallint} }_{2}^{{ { \bar{\d{\rm L}}} }^{\diamond }}}-1\right)}{{{{\sf (} \! \rotatebox{146}{\sf c}}}^{\divideontimes }-1}\right)\end{array}\right)$$
3. $${\mathfrak{t}}^{\ddagger }{\hat{C} }_{om.-1}=\left(\begin{array}{c}1-{{\text{log}}}_{{{{\sf (} \! \rotatebox{146}{\sf c}}}^{\divideontimes }}\left(1+\frac{{\left({{{{\sf (} \! \rotatebox{146}{\sf c}}}^{\divideontimes }}^{1-{\mathfrak{L}}_{1}^{{\rotatebox{180}{{\rm h}}}^{\diamond }}}-1\right)}^{{\mathfrak{t}}^{\ddagger }}}{{\left({{{\sf (} \! \rotatebox{146}{\sf c}}}^{\divideontimes }-1\right)}^{{\mathfrak{t}}^{\ddagger }-1}}\right)+\iota 1-{{\text{log}}}_{{{{\sf (} \! \rotatebox{146}{\sf c}}}^{\divideontimes }}\left(1+\frac{{\left({{{{\sf (} \! \rotatebox{146}{\sf c}}}^{\divideontimes }}^{1-{ \frown\!\!\!\!\!\! {\smallint} }_{1}^{{\rotatebox{180}{{\rm h}}}^{\diamond }}}-1\right)}^{{\mathfrak{t}}^{\ddagger }}}{{\left({{{\sf (} \! \rotatebox{146}{\sf c}}}^{\divideontimes }-1\right)}^{v-1}}\right), \\ 1-{{\text{log}}}_{{{{\sf (} \! \rotatebox{146}{\sf c}}}^{\divideontimes }}\left(1+\frac{{\left({{{{\sf (} \! \rotatebox{146}{\sf c}}}^{\divideontimes }}^{1-{\mathfrak{L}}_{1}^{{ { \bar{\d{\rm L}}} }^{\diamond }}}-1\right)}^{{\mathfrak{t}}^{\ddagger }}}{{\left({{{\sf (} \! \rotatebox{146}{\sf c}}}^{\divideontimes }-1\right)}^{{\mathfrak{t}}^{\ddagger }-1}}\right)+\iota 1-{{\text{log}}}_{{{{\sf (} \! \rotatebox{146}{\sf c}}}^{\divideontimes }}\left(1+\frac{{\left({{{{\sf (} \! \rotatebox{146}{\sf c}}}^{\divideontimes }}^{1-{ \frown\!\!\!\!\!\! {\smallint} }_{1}^{{ { \bar{\d{\rm L}}} }^{\diamond }}}-1\right)}^{{\mathfrak{t}}^{\ddagger }}}{{\left({{{\sf (} \! \rotatebox{146}{\sf c}}}^{\divideontimes }-1\right)}^{{\mathfrak{t}}^{\ddagger }-1}}\right)\end{array}\right)$$
4. $${\left({\hat{C} }_{om.-1}\right)}^{{\mathfrak{t}}^{\ddagger }}=\left(\begin{array}{c}{{\text{log}}}_{{{{\sf (} \! \rotatebox{146}{\sf c}}}^{\divideontimes }}\left(1+\frac{{\left({{{{\sf (} \! \rotatebox{146}{\sf c}}}^{\divideontimes }}^{{\mathfrak{L}}_{1}^{{\rotatebox{180}{{\rm h}}}^{\diamond }}}-1\right)}^{{\mathfrak{t}}^{\ddagger }}}{{\left({{{\sf (} \! \rotatebox{146}{\sf c}}}^{\divideontimes }-1\right)}^{{\mathfrak{t}}^{\ddagger }-1}}\right)+\iota {{\text{log}}}_{{{{\sf (} \! \rotatebox{146}{\sf c}}}^{\divideontimes }}\left(1+\frac{{\left({{{{\sf (} \! \rotatebox{146}{\sf c}}}^{\divideontimes }}^{{ \frown\!\!\!\!\!\! {\smallint} }_{1}^{{\rotatebox{180}{{\rm h}}}^{\diamond }}}-1\right)}^{{\mathfrak{t}}^{\ddagger }}}{{\left({{{\sf (} \! \rotatebox{146}{\sf c}}}^{\divideontimes }-1\right)}^{{\mathfrak{t}}^{\ddagger }-1}}\right),\\ {{\text{log}}}_{{{{\sf (} \! \rotatebox{146}{\sf c}}}^{\divideontimes }}\left(1+\frac{{\left({{{{\sf (} \! \rotatebox{146}{\sf c}}}^{\divideontimes }}^{{\mathfrak{L}}_{1}^{{ { \bar{\d{\rm L}}} }^{\diamond }}}-1\right)}^{{\mathfrak{t}}^{\ddagger }}}{{\left({{{\sf (} \! \rotatebox{146}{\sf c}}}^{\divideontimes }-1\right)}^{{\mathfrak{t}}^{\ddagger }-1}}\right)+\iota {{\text{log}}}_{{{{\sf (} \! \rotatebox{146}{\sf c}}}^{\divideontimes }}\left(1+\frac{{\left({{{{\sf (} \! \rotatebox{146}{\sf c}}}^{\divideontimes }}^{{ \frown\!\!\!\!\!\! {\smallint} }_{1}^{{ { \bar{\d{\rm L}}} }^{\diamond }}}-1\right)}^{{\mathfrak{t}}^{\ddagger }}}{{\left({{{\sf (} \! \rotatebox{146}{\sf c}}}^{\divideontimes }-1\right)}^{{\mathfrak{t}}^{\ddagger }-1}}\right)\end{array}\right)$$

#### Definition 9

Let ${C}_{om.-1}=\left({\mathfrak{L}}_{1}^{{\rotatebox{180}{{\rm h}}}^{\diamond }}+\iota { \frown\!\!\!\!\!\! {\smallint} }_{1}^{{\rotatebox{180}{{\rm h}}}^{\diamond }}, {\mathfrak{L}}_{1}^{{ { \bar{\d{\rm L}}} }^{\diamond }}+\iota { \frown\!\!\!\!\!\! {\smallint} }_{1}^{{ { \bar{\d{\rm L}}} }^{\diamond }}\right)$ be a CFRN, then the score function and accuracy function are defined as

$$Scr.\left({ \varsigma}_{1}\right)=\frac{1}{4}\left({\mathfrak{L}}_{1}^{{\rotatebox{180}{{\rm h}}}^{\diamond }}+{ \frown\!\!\!\!\!\! {\smallint} }_{1}^{{\rotatebox{180}{{\rm h}}}^{\diamond }}+{\mathfrak{L}}_{1}^{{ { \bar{\d{\rm L}}} }^{\diamond }}+{ \frown\!\!\!\!\!\! {\smallint} }_{1}^{{ { \bar{\d{\rm L}}} }^{\diamond }}\right);Scr.\in \left[0, 1\right]$$

And

$$Ac.\left({ \varsigma}_{1}\right)=\frac{1}{4}\left({\mathfrak{L}}_{1}^{{\rotatebox{180}{{\rm h}}}^{\diamond }}+{ \frown\!\!\!\!\!\! {\smallint} }_{1}^{{\rotatebox{180}{{\rm h}}}^{\diamond }}-{\mathfrak{L}}_{1}^{{ { \bar{\d{\rm L}}} }^{\diamond }}-{ \frown\!\!\!\!\!\! {\smallint} }_{1}^{{ { \bar{\d{\rm L}}} }^{\diamond }}\right);Ac.\in \left[-1, 1\right]$$

#### Theorem 1

*Let* ${\hat{C} }_{om.-1}=\left({\mathfrak{L}}_{1}^{{\rotatebox{180}{{\rm h}}}^{\diamond }}+\iota { \frown\!\!\!\!\!\! {\smallint} }_{1}^{{\rotatebox{180}{{\rm h}}}^{\diamond }}, {\mathfrak{L}}_{1}^{{ { \bar{\d{\rm L}}} }^{\diamond }}+\iota { \frown\!\!\!\!\!\! {\smallint} }_{1}^{{ { \bar{\d{\rm L}}} }^{\diamond }}\right)$ *and* ${\hat{C} }_{om.-2}=\left({\mathfrak{L}}_{2}^{{\rotatebox{180}{{\rm h}}}^{\diamond }}+\iota { \frown\!\!\!\!\!\! {\smallint} }_{2}^{{\rotatebox{180}{{\rm h}}}^{\diamond }}, {\mathfrak{L}}_{2}^{{ { \bar{\d{\rm L}}} }^{\diamond }}+\iota { \frown\!\!\!\!\!\! {\smallint} }_{2}^{{ { \bar{\d{\rm L}}} }^{\diamond }}\right)$ *be two CFRNs*, ${{{\sf (} \! \rotatebox{146}{\sf c}}}^{\divideontimes }>1$, *and* ${\mathfrak{t}}^{\ddagger }, {\mathfrak{t}}_{1}^{\ddagger }, {\mathfrak{t}}_{2}^{\ddagger }>0$ *is any real number. Then we get*

1. $${\hat{C} }_{om.-1}\oplus {\hat{C} }_{om.-2}={\hat{C} }_{om.-2}\oplus {\hat{C} }_{om.-1}$$
2. $${\hat{C} }_{om.-1}\otimes {\hat{C} }_{om.-2}={\hat{C} }_{om.-2}\otimes {\hat{C} }_{om.-1}$$
3. $${\mathfrak{t}}^{\ddagger }\left({\hat{C} }_{om.-1}\oplus {\hat{C} }_{om.-2}\right)={\mathfrak{t}}^{\ddagger }{\hat{C} }_{om.-1}\oplus {\mathfrak{t}}^{\ddagger }{\hat{C} }_{om.-2}$$
4. $${\left({\hat{C} }_{om.-1}\otimes {\hat{C} }_{om.-2}\right)}^{{\mathfrak{t}}^{\ddagger }}={{\hat{C} }_{om.-1}}^{{\mathfrak{t}}^{\ddagger }}\otimes {{\hat{C} }_{om.-2}}^{{\mathfrak{t}}^{\ddagger }}$$
5. $${{\mathfrak{t}}^{\ddagger }}_{1}{\hat{C} }_{om.-1}\oplus {{\mathfrak{t}}^{\ddagger }}_{2}{\hat{C} }_{om.-1}=\left({{\mathfrak{t}}^{\ddagger }}_{1}+{{\mathfrak{t}}^{\ddagger }}_{2}\right){\hat{C} }_{om.-1}$$
6. $${{\hat{C} }_{om.-1}}^{{{\mathfrak{t}}^{\ddagger }}_{1}}\otimes {{\hat{C} }_{om.-1}}^{{{\mathfrak{t}}^{\ddagger }}_{2}}={{\hat{C} }_{om.-1}}^{{{\mathfrak{t}}^{\ddagger }}_{1}+{{\mathfrak{t}}^{\ddagger }}_{2}}$$
7. $${\left({{\hat{C} }_{om.-1}}^{{{\mathfrak{t}}^{\ddagger }}_{1}}\right)}^{{{\mathfrak{t}}^{\ddagger }}_{2}}={{\hat{C} }_{om.-1}}^{{{\mathfrak{t}}^{\ddagger }}_{1}{{\mathfrak{t}}^{\ddagger }}_{2}}$$.

#### Proof

1. By using Definition 8, we get
  $${\hat{C} }_{om.-1}\oplus {\hat{C} }_{om.-2}=\left(\begin{array}{c}1-{{\text{log}}}_{{{{\sf (} \! \rotatebox{146}{\sf c}}}^{\divideontimes }}\left(1+\frac{\left({{{{\sf (} \! \rotatebox{146}{\sf c}}}^{\divideontimes }}^{1-{\mathfrak{L}}_{1}^{{\rotatebox{180}{{\rm h}}}^{\diamond }}}-1\right)\left({{{{\sf (} \! \rotatebox{146}{\sf c}}}^{\divideontimes }}^{1-{\mathfrak{L}}_{2}^{{\rotatebox{180}{{\rm h}}}^{\diamond }}}-1\right)}{{{{\sf (} \! \rotatebox{146}{\sf c}}}^{\divideontimes }-1}\right)+\iota 1-{{\text{log}}}_{{{{\sf (} \! \rotatebox{146}{\sf c}}}^{\divideontimes }}\left(1+\frac{\left({{{{\sf (} \! \rotatebox{146}{\sf c}}}^{\divideontimes }}^{1-{ \frown\!\!\!\!\!\! {\smallint} }_{1}^{{\rotatebox{180}{{\rm h}}}^{\diamond }}}-1\right)\left({{{{\sf (} \! \rotatebox{146}{\sf c}}}^{\divideontimes }}^{1-{ \frown\!\!\!\!\!\! {\smallint} }_{2}^{{\rotatebox{180}{{\rm h}}}^{\diamond }}}-1\right)}{{{{\sf (} \! \rotatebox{146}{\sf c}}}^{\divideontimes }-1}\right),\\ 1-{{\text{log}}}_{{{{\sf (} \! \rotatebox{146}{\sf c}}}^{\divideontimes }}\left(1+\frac{\left({{{{\sf (} \! \rotatebox{146}{\sf c}}}^{\divideontimes }}^{1-{\mathfrak{L}}_{1}^{{ { \bar{\d{\rm L}}} }^{\diamond }}}-1\right)\left({{{{\sf (} \! \rotatebox{146}{\sf c}}}^{\divideontimes }}^{1-{\mathfrak{L}}_{2}^{{ { \bar{\d{\rm L}}} }^{\diamond }}}-1\right)}{{{{\sf (} \! \rotatebox{146}{\sf c}}}^{\divideontimes }-1}\right)+\iota 1-{{\text{log}}}_{{{{\sf (} \! \rotatebox{146}{\sf c}}}^{\divideontimes }}\left(1+\frac{\left({{{{\sf (} \! \rotatebox{146}{\sf c}}}^{\divideontimes }}^{1-{ \frown\!\!\!\!\!\! {\smallint} }_{1}^{{ { \bar{\d{\rm L}}} }^{\diamond }}}-1\right)\left({{{{\sf (} \! \rotatebox{146}{\sf c}}}^{\divideontimes }}^{1-{ \frown\!\!\!\!\!\! {\smallint} }_{2}^{{ { \bar{\d{\rm L}}} }^{\diamond }}}-1\right)}{{{{\sf (} \! \rotatebox{146}{\sf c}}}^{\divideontimes }-1}\right)\end{array}\right)$$
  $$=\left(\begin{array}{c}1-{{\text{log}}}_{{{{\sf (} \! \rotatebox{146}{\sf c}}}^{\divideontimes }}\left(1+\frac{\left({{{{\sf (} \! \rotatebox{146}{\sf c}}}^{\divideontimes }}^{1-{\mathfrak{L}}_{2}^{{\rotatebox{180}{{\rm h}}}^{\diamond }}}-1\right)\left({{{{\sf (} \! \rotatebox{146}{\sf c}}}^{\divideontimes }}^{1-{\mathfrak{L}}_{1}^{{\rotatebox{180}{{\rm h}}}^{\diamond }}}-1\right)}{{{{\sf (} \! \rotatebox{146}{\sf c}}}^{\divideontimes }-1}\right)+\iota 1-{{\text{log}}}_{{{{\sf (} \! \rotatebox{146}{\sf c}}}^{\divideontimes }}\left(1+\frac{\left({{{{\sf (} \! \rotatebox{146}{\sf c}}}^{\divideontimes }}^{1-{ \frown\!\!\!\!\!\! {\smallint} }_{2}^{{\rotatebox{180}{{\rm h}}}^{\diamond }}}-1\right)\left({{{{\sf (} \! \rotatebox{146}{\sf c}}}^{\divideontimes }}^{1-{ \frown\!\!\!\!\!\! {\smallint} }_{1}^{{\rotatebox{180}{{\rm h}}}^{\diamond }}}-1\right)}{{{{\sf (} \! \rotatebox{146}{\sf c}}}^{\divideontimes }-1}\right),\\ 1-{{\text{log}}}_{{{{\sf (} \! \rotatebox{146}{\sf c}}}^{\divideontimes }}\left(1+\frac{\left({{{{\sf (} \! \rotatebox{146}{\sf c}}}^{\divideontimes }}^{1-{\mathfrak{L}}_{2}^{{ { \bar{\d{\rm L}}} }^{\diamond }}}-1\right)\left({{{{\sf (} \! \rotatebox{146}{\sf c}}}^{\divideontimes }}^{1-{\mathfrak{L}}_{1}^{{ { \bar{\d{\rm L}}} }^{\diamond }}}-1\right)}{{{{\sf (} \! \rotatebox{146}{\sf c}}}^{\divideontimes }-1}\right)+\iota 1-{{\text{log}}}_{{{{\sf (} \! \rotatebox{146}{\sf c}}}^{\divideontimes }}\left(1+\frac{\left({{{{\sf (} \! \rotatebox{146}{\sf c}}}^{\divideontimes }}^{1-{ \frown\!\!\!\!\!\! {\smallint} }_{2}^{{ { \bar{\d{\rm L}}} }^{\diamond }}}-1\right)\left({{{{\sf (} \! \rotatebox{146}{\sf c}}}^{\divideontimes }}^{1-{ \frown\!\!\!\!\!\! {\smallint} }_{1}^{{ { \bar{\d{\rm L}}} }^{\diamond }}}-1\right)}{{{{\sf (} \! \rotatebox{146}{\sf c}}}^{\divideontimes }-1}\right)\end{array}\right)$$
  $$={\hat{C} }_{om.-2}\oplus {\hat{C} }_{om.-1}$$
2. Proof of (2) is similar to 1.
3. We have
  $${\mathfrak{t}}^{\ddagger }\left({\hat{C} }_{om.-1}\otimes {\hat{C} }_{om.-2}\right)={\mathfrak{t}}^{\ddagger }\left(\begin{array}{c}{{\text{log}}}_{{{{\sf (} \! \rotatebox{146}{\sf c}}}^{\divideontimes }}\left(1+\frac{\left({{{{\sf (} \! \rotatebox{146}{\sf c}}}^{\divideontimes }}^{{\mathfrak{L}}_{1}^{{\rotatebox{180}{{\rm h}}}^{\diamond }}}-1\right)\left({{{{\sf (} \! \rotatebox{146}{\sf c}}}^{\divideontimes }}^{{\mathfrak{L}}_{2}^{{\rotatebox{180}{{\rm h}}}^{\diamond }}}-1\right)}{{{{\sf (} \! \rotatebox{146}{\sf c}}}^{\divideontimes }-1}\right)+\iota {{\text{log}}}_{{{{\sf (} \! \rotatebox{146}{\sf c}}}^{\divideontimes }}\left(1+\frac{\left({{{{\sf (} \! \rotatebox{146}{\sf c}}}^{\divideontimes }}^{{ \frown\!\!\!\!\!\! {\smallint} }_{1}^{{\rotatebox{180}{{\rm h}}}^{\diamond }}}-1\right)\left({{{{\sf (} \! \rotatebox{146}{\sf c}}}^{\divideontimes }}^{{ \frown\!\!\!\!\!\! {\smallint} }_{2}^{{\rotatebox{180}{{\rm h}}}^{\diamond }}}-1\right)}{{{{\sf (} \! \rotatebox{146}{\sf c}}}^{\divideontimes }-1}\right),\\ {{\text{log}}}_{{{{\sf (} \! \rotatebox{146}{\sf c}}}^{\divideontimes }}\left(1+\frac{\left({{{{\sf (} \! \rotatebox{146}{\sf c}}}^{\divideontimes }}^{{\mathfrak{L}}_{1}^{{ { \bar{\d{\rm L}}} }^{\diamond }}}-1\right)\left({{{{\sf (} \! \rotatebox{146}{\sf c}}}^{\divideontimes }}^{{\mathfrak{L}}_{2}^{{ { \bar{\d{\rm L}}} }^{\diamond }}}-1\right)}{{{{\sf (} \! \rotatebox{146}{\sf c}}}^{\divideontimes }-1}\right)+\iota {{\text{log}}}_{{{{\sf (} \! \rotatebox{146}{\sf c}}}^{\divideontimes }}\left(1+\frac{\left({{{{\sf (} \! \rotatebox{146}{\sf c}}}^{\divideontimes }}^{{ \frown\!\!\!\!\!\! {\smallint} }_{1}^{{ { \bar{\d{\rm L}}} }^{\diamond }}}-1\right)\left({{{{\sf (} \! \rotatebox{146}{\sf c}}}^{\divideontimes }}^{{ \frown\!\!\!\!\!\! {\smallint} }_{2}^{{ { \bar{\d{\rm L}}} }^{\diamond }}}-1\right)}{{{{\sf (} \! \rotatebox{146}{\sf c}}}^{\divideontimes }-1}\right)\end{array}\right)$$
  $$=\left(\begin{array}{c}{{\text{log}}}_{{{{\sf (} \! \rotatebox{146}{\sf c}}}^{\divideontimes }}\left(1+\frac{{\left({{{{\sf (} \! \rotatebox{146}{\sf c}}}^{\divideontimes }}^{{\mathfrak{L}}_{1}^{{\rotatebox{180}{{\rm h}}}^{\diamond }}}-1\right)}^{{\mathfrak{t}}^{\ddagger }}{\left({{{{\sf (} \! \rotatebox{146}{\sf c}}}^{\divideontimes }}^{{\mathfrak{L}}_{2}^{{\rotatebox{180}{{\rm h}}}^{\diamond }}}-1\right)}^{{\mathfrak{t}}^{\ddagger }}}{{\left({{{\sf (} \! \rotatebox{146}{\sf c}}}^{\divideontimes }-1\right)}^{2{\mathfrak{t}}^{\ddagger }-1}}\right)+\iota {{\text{log}}}_{{{{\sf (} \! \rotatebox{146}{\sf c}}}^{\divideontimes }}\left(1+\frac{{\left({{{{\sf (} \! \rotatebox{146}{\sf c}}}^{\divideontimes }}^{{ \frown\!\!\!\!\!\! {\smallint} }_{1}^{{\rotatebox{180}{{\rm h}}}^{\diamond }}}-1\right)}^{{\mathfrak{t}}^{\ddagger }}{\left({{{{\sf (} \! \rotatebox{146}{\sf c}}}^{\divideontimes }}^{{ \frown\!\!\!\!\!\! {\smallint} }_{2}^{{\rotatebox{180}{{\rm h}}}^{\diamond }}}-1\right)}^{{\mathfrak{t}}^{\ddagger }}}{{\left({{{\sf (} \! \rotatebox{146}{\sf c}}}^{\divideontimes }-1\right)}^{{2\mathfrak{t}}^{\ddagger }-1}}\right),\\ {{\text{log}}}_{{{{\sf (} \! \rotatebox{146}{\sf c}}}^{\divideontimes }}\left(1+\frac{{\left({{{{\sf (} \! \rotatebox{146}{\sf c}}}^{\divideontimes }}^{{\mathfrak{L}}_{1}^{{ { \bar{\d{\rm L}}} }^{\diamond }}}-1\right)}^{{\mathfrak{t}}^{\ddagger }}{\left({{{{\sf (} \! \rotatebox{146}{\sf c}}}^{\divideontimes }}^{{\mathfrak{L}}_{2}^{{ { \bar{\d{\rm L}}} }^{\diamond }}}-1\right)}^{{\mathfrak{t}}^{\ddagger }}}{{\left({{{\sf (} \! \rotatebox{146}{\sf c}}}^{\divideontimes }-1\right)}^{{2\mathfrak{t}}^{\ddagger }-1}}\right)+\iota {{\text{log}}}_{{{{\sf (} \! \rotatebox{146}{\sf c}}}^{\divideontimes }}\left(1+\frac{{\left({{{{\sf (} \! \rotatebox{146}{\sf c}}}^{\divideontimes }}^{{ \frown\!\!\!\!\!\! {\smallint} }_{1}^{{ { \bar{\d{\rm L}}} }^{\diamond }}}-1\right)}^{{\mathfrak{t}}^{\ddagger }}{\left({{{{\sf (} \! \rotatebox{146}{\sf c}}}^{\divideontimes }}^{{ \frown\!\!\!\!\!\! {\smallint} }_{2}^{{ { \bar{\d{\rm L}}} }^{\diamond }}}-1\right)}^{{\mathfrak{t}}^{\ddagger }}}{{\left({{{\sf (} \! \rotatebox{146}{\sf c}}}^{\divideontimes }-1\right)}^{2{\mathfrak{t}}^{\ddagger }-1}}\right)\end{array}\right)$$
  And
  $${\mathfrak{t}}^{\ddagger }{\hat{C} }_{om.-1}=\left(\begin{array}{c}1-{{\text{log}}}_{{{{\sf (} \! \rotatebox{146}{\sf c}}}^{\divideontimes }}\left(1+\frac{{\left({{{{\sf (} \! \rotatebox{146}{\sf c}}}^{\divideontimes }}^{1-{\mathfrak{L}}_{1}^{{\rotatebox{180}{{\rm h}}}^{\diamond }}}-1\right)}^{{\mathfrak{t}}^{\ddagger }}}{{\left({{{\sf (} \! \rotatebox{146}{\sf c}}}^{\divideontimes }-1\right)}^{{\mathfrak{t}}^{\ddagger }-1}}\right)+\iota {1-{\text{log}}}_{{{{\sf (} \! \rotatebox{146}{\sf c}}}^{\divideontimes }}\left(1+\frac{{\left({{{{\sf (} \! \rotatebox{146}{\sf c}}}^{\divideontimes }}^{{1- \frown\!\!\!\!\!\! {\smallint} }_{1}^{{\rotatebox{180}{{\rm h}}}^{\diamond }}}-1\right)}^{{\mathfrak{t}}^{\ddagger }}}{{\left({{{\sf (} \! \rotatebox{146}{\sf c}}}^{\divideontimes }-1\right)}^{{\mathfrak{t}}^{\ddagger }-1}}\right),\\ 1-{{\text{log}}}_{{{{\sf (} \! \rotatebox{146}{\sf c}}}^{\divideontimes }}\left(1+\frac{{\left({{{{\sf (} \! \rotatebox{146}{\sf c}}}^{\divideontimes }}^{1-{\mathfrak{L}}_{1}^{{ { \bar{\d{\rm L}}} }^{\diamond }}}-1\right)}^{{\mathfrak{t}}^{\ddagger }}}{{\left({{{\sf (} \! \rotatebox{146}{\sf c}}}^{\divideontimes }-1\right)}^{{\mathfrak{t}}^{\ddagger }-1}}\right)+\iota {1-{\text{log}}}_{{{{\sf (} \! \rotatebox{146}{\sf c}}}^{\divideontimes }}\left(1+\frac{{\left({{{{\sf (} \! \rotatebox{146}{\sf c}}}^{\divideontimes }}^{{1- \frown\!\!\!\!\!\! {\smallint} }_{1}^{{ { \bar{\d{\rm L}}} }^{\diamond }}}-1\right)}^{{\mathfrak{t}}^{\ddagger }}}{{\left({{{\sf (} \! \rotatebox{146}{\sf c}}}^{\divideontimes }-1\right)}^{{\mathfrak{t}}^{\ddagger }-1}}\right)\end{array}\right)$$
  $${\mathfrak{t}}^{\ddagger }{\hat{C} }_{om.-2}=\left(\begin{array}{c}1-{{\text{log}}}_{{{{\sf (} \! \rotatebox{146}{\sf c}}}^{\divideontimes }}\left(1+\frac{{\left({{{{\sf (} \! \rotatebox{146}{\sf c}}}^{\divideontimes }}^{1-{\mathfrak{L}}_{2}^{{\rotatebox{180}{{\rm h}}}^{\diamond }}}-1\right)}^{{\mathfrak{t}}^{\ddagger }}}{{\left({{{\sf (} \! \rotatebox{146}{\sf c}}}^{\divideontimes }-1\right)}^{{\mathfrak{t}}^{\ddagger }-1}}\right)+\iota {1-{\text{log}}}_{{{{\sf (} \! \rotatebox{146}{\sf c}}}^{\divideontimes }}\left(1+\frac{{\left({{{{\sf (} \! \rotatebox{146}{\sf c}}}^{\divideontimes }}^{{1- \frown\!\!\!\!\!\! {\smallint} }_{2}^{{\rotatebox{180}{{\rm h}}}^{\diamond }}}-1\right)}^{{\mathfrak{t}}^{\ddagger }}}{{\left({{{\sf (} \! \rotatebox{146}{\sf c}}}^{\divideontimes }-1\right)}^{{\mathfrak{t}}^{\ddagger }-1}}\right),\\ 1-{{\text{log}}}_{{{{\sf (} \! \rotatebox{146}{\sf c}}}^{\divideontimes }}\left(1+\frac{{\left({{{{\sf (} \! \rotatebox{146}{\sf c}}}^{\divideontimes }}^{1-{\mathfrak{L}}_{2}^{{ { \bar{\d{\rm L}}} }^{\diamond }}}-1\right)}^{{\mathfrak{t}}^{\ddagger }}}{{\left({{{\sf (} \! \rotatebox{146}{\sf c}}}^{\divideontimes }-1\right)}^{{\mathfrak{t}}^{\ddagger }-1}}\right)+\iota {1-{\text{log}}}_{{{{\sf (} \! \rotatebox{146}{\sf c}}}^{\divideontimes }}\left(1+\frac{{\left({{{{\sf (} \! \rotatebox{146}{\sf c}}}^{\divideontimes }}^{{1- \frown\!\!\!\!\!\! {\smallint} }_{2}^{{ { \bar{\d{\rm L}}} }^{\diamond }}}-1\right)}^{{\mathfrak{t}}^{\ddagger }}}{{\left({{{\sf (} \! \rotatebox{146}{\sf c}}}^{\divideontimes }-1\right)}^{{\mathfrak{t}}^{\ddagger }-1}}\right)\end{array}\right)$$
  Then,
  $$\begin{aligned} & {\mathfrak{t}}^{\ddagger }{\hat{C} }_{om.-1}\oplus {\mathfrak{t}}^{\ddagger }{\hat{C} }_{om.-2} \\ & \quad =\left(\begin{array}{c}1-{{\text{log}}}_{{{{\sf (} \! \rotatebox{146}{\sf c}}}^{\divideontimes }}\left(1+\frac{{\left({{{{\sf (} \! \rotatebox{146}{\sf c}}}^{\divideontimes }}^{1-{\mathfrak{L}}_{1}^{{\rotatebox{180}{{\rm h}}}^{\diamond }}}-1\right)}^{{\mathfrak{t}}^{\ddagger }}}{{\left({{{\sf (} \! \rotatebox{146}{\sf c}}}^{\divideontimes }-1\right)}^{{\mathfrak{t}}^{\ddagger }-1}}\right)+\iota 1-{{\text{log}}}_{{{{\sf (} \! \rotatebox{146}{\sf c}}}^{\divideontimes }}\left(1+\frac{{\left({{{{\sf (} \! \rotatebox{146}{\sf c}}}^{\divideontimes }}^{1-{ \frown\!\!\!\!\!\! {\smallint} }_{1}^{{\rotatebox{180}{{\rm h}}}^{\diamond }}}-1\right)}^{{\mathfrak{t}}^{\ddagger }}}{{\left({{{\sf (} \! \rotatebox{146}{\sf c}}}^{\divideontimes }-1\right)}^{{\mathfrak{t}}^{\ddagger }-1}}\right), \\ 1-{{\text{log}}}_{{{{\sf (} \! \rotatebox{146}{\sf c}}}^{\divideontimes }}\left(1+\frac{{\left({{{{\sf (} \! \rotatebox{146}{\sf c}}}^{\divideontimes }}^{1-{\mathfrak{L}}_{1}^{{ { \bar{\d{\rm L}}} }^{\diamond }}}-1\right)}^{{\mathfrak{t}}^{\ddagger }}}{{\left({{{\sf (} \! \rotatebox{146}{\sf c}}}^{\divideontimes }-1\right)}^{{\mathfrak{t}}^{\ddagger }-1}}\right)+\iota 1-{{\text{log}}}_{{{{\sf (} \! \rotatebox{146}{\sf c}}}^{\divideontimes }}\left(1+\frac{{\left({{{{\sf (} \! \rotatebox{146}{\sf c}}}^{\divideontimes }}^{1-{ \frown\!\!\!\!\!\! {\smallint} }_{1}^{{ { \bar{\d{\rm L}}} }^{\diamond }}}-1\right)}^{{\mathfrak{t}}^{\ddagger }}}{{\left({{{\sf (} \! \rotatebox{146}{\sf c}}}^{\divideontimes }-1\right)}^{{\mathfrak{t}}^{\ddagger }-1}}\right)\end{array}\right)\\ & \qquad \oplus \left(\begin{array}{c}1-{{\text{log}}}_{{{{\sf (} \! \rotatebox{146}{\sf c}}}^{\divideontimes }}\left(1+\frac{{\left({{{{\sf (} \! \rotatebox{146}{\sf c}}}^{\divideontimes }}^{1-{\mathfrak{L}}_{2}^{{\rotatebox{180}{{\rm h}}}^{\diamond }}}-1\right)}^{{\mathfrak{t}}^{\ddagger }}}{{\left({{{\sf (} \! \rotatebox{146}{\sf c}}}^{\divideontimes }-1\right)}^{{\mathfrak{t}}^{\ddagger }-1}}\right)+\iota 1-{{\text{log}}}_{{{{\sf (} \! \rotatebox{146}{\sf c}}}^{\divideontimes }}\left(1+\frac{{\left({{{{\sf (} \! \rotatebox{146}{\sf c}}}^{\divideontimes }}^{1-{ \frown\!\!\!\!\!\! {\smallint} }_{2}^{{\rotatebox{180}{{\rm h}}}^{\diamond }}}-1\right)}^{{\mathfrak{t}}^{\ddagger }}}{{\left({{{\sf (} \! \rotatebox{146}{\sf c}}}^{\divideontimes }-1\right)}^{{\mathfrak{t}}^{\ddagger }-1}}\right), \\ 1-{{\text{log}}}_{{{{\sf (} \! \rotatebox{146}{\sf c}}}^{\divideontimes }}\left(1+\frac{{\left({{{{\sf (} \! \rotatebox{146}{\sf c}}}^{\divideontimes }}^{1-{\mathfrak{L}}_{2}^{{ { \bar{\d{\rm L}}} }^{\diamond }}}-1\right)}^{{\mathfrak{t}}^{\ddagger }}}{{\left({{{\sf (} \! \rotatebox{146}{\sf c}}}^{\divideontimes }-1\right)}^{{\mathfrak{t}}^{\ddagger }-1}}\right)+\iota 1-{{\text{log}}}_{{{{\sf (} \! \rotatebox{146}{\sf c}}}^{\divideontimes }}\left(1+\frac{{\left({{{{\sf (} \! \rotatebox{146}{\sf c}}}^{\divideontimes }}^{1-{ \frown\!\!\!\!\!\! {\smallint} }_{2}^{{ { \bar{\d{\rm L}}} }^{\diamond }}}-1\right)}^{{\mathfrak{t}}^{\ddagger }}}{{\left({{{\sf (} \! \rotatebox{146}{\sf c}}}^{\divideontimes }-1\right)}^{{\mathfrak{t}}^{\ddagger }-1}}\right)\end{array}\right)\end{aligned}$$
  $$=\left(\begin{array}{c}{{\text{log}}}_{{{{\sf (} \! \rotatebox{146}{\sf c}}}^{\divideontimes }}\left(1+\frac{{\left({{{{\sf (} \! \rotatebox{146}{\sf c}}}^{\divideontimes }}^{{\mathfrak{L}}_{1}^{{\rotatebox{180}{{\rm h}}}^{\diamond }}}-1\right)}^{{\mathfrak{t}}^{\ddagger }}{\left({{{{\sf (} \! \rotatebox{146}{\sf c}}}^{\divideontimes }}^{{\mathfrak{L}}_{2}^{{\rotatebox{180}{{\rm h}}}^{\diamond }}}-1\right)}^{{\mathfrak{t}}^{\ddagger }}}{{\left({{{\sf (} \! \rotatebox{146}{\sf c}}}^{\divideontimes }-1\right)}^{2{\mathfrak{t}}^{\ddagger }-1}}\right)+\iota {{\text{log}}}_{{{{\sf (} \! \rotatebox{146}{\sf c}}}^{\divideontimes }}\left(1+\frac{{\left({{{{\sf (} \! \rotatebox{146}{\sf c}}}^{\divideontimes }}^{{ \frown\!\!\!\!\!\! {\smallint} }_{1}^{{\rotatebox{180}{{\rm h}}}^{\diamond }}}-1\right)}^{{\mathfrak{t}}^{\ddagger }}{\left({{{{\sf (} \! \rotatebox{146}{\sf c}}}^{\divideontimes }}^{{ \frown\!\!\!\!\!\! {\smallint} }_{2}^{{\rotatebox{180}{{\rm h}}}^{\diamond }}}-1\right)}^{{\mathfrak{t}}^{\ddagger }}}{{\left({{{\sf (} \! \rotatebox{146}{\sf c}}}^{\divideontimes }-1\right)}^{{2\mathfrak{t}}^{\ddagger }-1}}\right),\\ {{\text{log}}}_{{{{\sf (} \! \rotatebox{146}{\sf c}}}^{\divideontimes }}\left(1+\frac{{\left({{{{\sf (} \! \rotatebox{146}{\sf c}}}^{\divideontimes }}^{{\mathfrak{L}}_{1}^{{ { \bar{\d{\rm L}}} }^{\diamond }}}-1\right)}^{{\mathfrak{t}}^{\ddagger }}{\left({{{{\sf (} \! \rotatebox{146}{\sf c}}}^{\divideontimes }}^{{\mathfrak{L}}_{2}^{{ { \bar{\d{\rm L}}} }^{\diamond }}}-1\right)}^{{\mathfrak{t}}^{\ddagger }}}{{\left({{{\sf (} \! \rotatebox{146}{\sf c}}}^{\divideontimes }-1\right)}^{{2\mathfrak{t}}^{\ddagger }-1}}\right)+\iota {{\text{log}}}_{{{{\sf (} \! \rotatebox{146}{\sf c}}}^{\divideontimes }}\left(1+\frac{{\left({{{{\sf (} \! \rotatebox{146}{\sf c}}}^{\divideontimes }}^{{ \frown\!\!\!\!\!\! {\smallint} }_{1}^{{ { \bar{\d{\rm L}}} }^{\diamond }}}-1\right)}^{{\mathfrak{t}}^{\ddagger }}{\left({{{{\sf (} \! \rotatebox{146}{\sf c}}}^{\divideontimes }}^{{ \frown\!\!\!\!\!\! {\smallint} }_{2}^{{ { \bar{\d{\rm L}}} }^{\diamond }}}-1\right)}^{{\mathfrak{t}}^{\ddagger }}}{{\left({{{\sf (} \! \rotatebox{146}{\sf c}}}^{\divideontimes }-1\right)}^{2{\mathfrak{t}}^{\ddagger }-1}}\right)\end{array}\right)$$
  Hence, $${\mathfrak{t}}^{\ddagger }\left({\hat{C} }_{om.-1}\oplus {\hat{C} }_{om.-2}\right)={\mathfrak{t}}^{\ddagger }{\hat{C} }_{om.-1}\oplus {\mathfrak{t}}^{\ddagger }{\hat{C} }_{om.-2}$$.
4. We have,
  $$\begin{aligned} & {\left({\hat{C} }_{om.-1}\otimes {\hat{C} }_{om.-2}\right)}^{{\mathfrak{t}}^{\ddagger }}\\ & \quad ={\left(\begin{array}{c}{{\text{log}}}_{{{{\sf (} \! \rotatebox{146}{\sf c}}}^{\divideontimes }}\left(1+\frac{\left({{{{\sf (} \! \rotatebox{146}{\sf c}}}^{\divideontimes }}^{{\mathfrak{L}}_{1}^{{\rotatebox{180}{{\rm h}}}^{\diamond }}}-1\right)\left({{{{\sf (} \! \rotatebox{146}{\sf c}}}^{\divideontimes }}^{{\mathfrak{L}}_{2}^{{\rotatebox{180}{{\rm h}}}^{\diamond }}}-1\right)}{{{{\sf (} \! \rotatebox{146}{\sf c}}}^{\divideontimes }-1}\right)+\iota {{\text{log}}}_{{{{\sf (} \! \rotatebox{146}{\sf c}}}^{\divideontimes }}\left(1+\frac{\left({{{{\sf (} \! \rotatebox{146}{\sf c}}}^{\divideontimes }}^{{ \frown\!\!\!\!\!\! {\smallint} }_{1}^{{\rotatebox{180}{{\rm h}}}^{\diamond }}}-1\right)\left({{{{\sf (} \! \rotatebox{146}{\sf c}}}^{\divideontimes }}^{{ \frown\!\!\!\!\!\! {\smallint} }_{2}^{{\rotatebox{180}{{\rm h}}}^{\diamond }}}-1\right)}{{{{\sf (} \! \rotatebox{146}{\sf c}}}^{\divideontimes }-1}\right),\\ {{\text{log}}}_{{{{\sf (} \! \rotatebox{146}{\sf c}}}^{\divideontimes }}\left(1+\frac{\left({{{{\sf (} \! \rotatebox{146}{\sf c}}}^{\divideontimes }}^{{\mathfrak{L}}_{1}^{{ { \bar{\d{\rm L}}} }^{\diamond }}}-1\right)\left({{{{\sf (} \! \rotatebox{146}{\sf c}}}^{\divideontimes }}^{{\mathfrak{L}}_{2}^{{ { \bar{\d{\rm L}}} }^{\diamond }}}-1\right)}{{{{\sf (} \! \rotatebox{146}{\sf c}}}^{\divideontimes }-1}\right)+\iota {{\text{log}}}_{{{{\sf (} \! \rotatebox{146}{\sf c}}}^{\divideontimes }}\left(1+\frac{\left({{{{\sf (} \! \rotatebox{146}{\sf c}}}^{\divideontimes }}^{{ \frown\!\!\!\!\!\! {\smallint} }_{1}^{{ { \bar{\d{\rm L}}} }^{\diamond }}}-1\right)\left({{{{\sf (} \! \rotatebox{146}{\sf c}}}^{\divideontimes }}^{{ \frown\!\!\!\!\!\! {\smallint} }_{2}^{{ { \bar{\d{\rm L}}} }^{\diamond }}}-1\right)}{{{{\sf (} \! \rotatebox{146}{\sf c}}}^{\divideontimes }-1}\right)\end{array}\right)}^{{\mathfrak{t}}^{\ddagger }}\end{aligned}$$
  $$={\left(\begin{array}{c}{{\text{log}}}_{{{{\sf (} \! \rotatebox{146}{\sf c}}}^{\divideontimes }}\left(1+\frac{{\left(\left({{{{\sf (} \! \rotatebox{146}{\sf c}}}^{\divideontimes }}^{{\mathfrak{L}}_{1}^{{\rotatebox{180}{{\rm h}}}^{\diamond }}}-1\right)\left({{{{\sf (} \! \rotatebox{146}{\sf c}}}^{\divideontimes }}^{{\mathfrak{L}}_{2}^{{\rotatebox{180}{{\rm h}}}^{\diamond }}}-1\right)\right)}^{{\mathfrak{t}}^{\ddagger }}}{{\left({{{\sf (} \! \rotatebox{146}{\sf c}}}^{\divideontimes }-1\right)}^{{2\mathfrak{t}}^{\ddagger }-1}}\right)+\iota {{\text{log}}}_{{{{\sf (} \! \rotatebox{146}{\sf c}}}^{\divideontimes }}\left(1+\frac{{\left(\left({{{{\sf (} \! \rotatebox{146}{\sf c}}}^{\divideontimes }}^{{ \frown\!\!\!\!\!\! {\smallint} }_{1}^{{\rotatebox{180}{{\rm h}}}^{\diamond }}}-1\right)\left({{{{\sf (} \! \rotatebox{146}{\sf c}}}^{\divideontimes }}^{{ \frown\!\!\!\!\!\! {\smallint} }_{2}^{{\rotatebox{180}{{\rm h}}}^{\diamond }}}-1\right)\right)}^{{\mathfrak{t}}^{\ddagger }}}{{\left({{{\sf (} \! \rotatebox{146}{\sf c}}}^{\divideontimes }-1\right)}^{{2\mathfrak{t}}^{\ddagger }-1}}\right),\\ {{\text{log}}}_{{{{\sf (} \! \rotatebox{146}{\sf c}}}^{\divideontimes }}\left(1+\frac{{\left(\left({{{{\sf (} \! \rotatebox{146}{\sf c}}}^{\divideontimes }}^{{\mathfrak{L}}_{1}^{{ { \bar{\d{\rm L}}} }^{\diamond }}}-1\right)\left({{{{\sf (} \! \rotatebox{146}{\sf c}}}^{\divideontimes }}^{{\mathfrak{L}}_{2}^{{ { \bar{\d{\rm L}}} }^{\diamond }}}-1\right)\right)}^{{\mathfrak{t}}^{\ddagger }}}{{\left({{{\sf (} \! \rotatebox{146}{\sf c}}}^{\divideontimes }-1\right)}^{{2\mathfrak{t}}^{\ddagger }-1}}\right)+\iota {{\text{log}}}_{{{{\sf (} \! \rotatebox{146}{\sf c}}}^{\divideontimes }}\left(1+\frac{\left(\left({{{{\sf (} \! \rotatebox{146}{\sf c}}}^{\divideontimes }}^{{ \frown\!\!\!\!\!\! {\smallint} }_{1}^{{ { \bar{\d{\rm L}}} }^{\diamond }}}-1\right){\left({{{{\sf (} \! \rotatebox{146}{\sf c}}}^{\divideontimes }}^{{ \frown\!\!\!\!\!\! {\smallint} }_{2}^{{ { \bar{\d{\rm L}}} }^{\diamond }}}-1\right)}^{{\mathfrak{t}}^{\ddagger }}\right)}{{\left({{{\sf (} \! \rotatebox{146}{\sf c}}}^{\divideontimes }-1\right)}^{{2\mathfrak{t}}^{\ddagger }-1}}\right)\end{array}\right)}^{{\mathfrak{t}}^{\ddagger }}$$
  $$\begin{aligned} & =\left(\begin{array}{c}{{\text{log}}}_{{{{\sf (} \! \rotatebox{146}{\sf c}}}^{\divideontimes }}\left(1+\frac{{\left({{{{\sf (} \! \rotatebox{146}{\sf c}}}^{\divideontimes }}^{{\mathfrak{L}}_{1}^{{\rotatebox{180}{{\rm h}}}^{\diamond }}}-1\right)}^{{\mathfrak{t}}^{\ddagger }}}{{\left({{{\sf (} \! \rotatebox{146}{\sf c}}}^{\divideontimes }-1\right)}^{{\mathfrak{t}}^{\ddagger }-1}}\right)+\iota {{\text{log}}}_{{{{\sf (} \! \rotatebox{146}{\sf c}}}^{\divideontimes }}\left(1+\frac{{\left({{{{\sf (} \! \rotatebox{146}{\sf c}}}^{\divideontimes }}^{{ \frown\!\!\!\!\!\! {\smallint} }_{1}^{{\rotatebox{180}{{\rm h}}}^{\diamond }}}-1\right)}^{{\mathfrak{t}}^{\ddagger }}}{{\left({{{\sf (} \! \rotatebox{146}{\sf c}}}^{\divideontimes }-1\right)}^{{\mathfrak{t}}^{\ddagger }-1}}\right),\\ {{\text{log}}}_{{{{\sf (} \! \rotatebox{146}{\sf c}}}^{\divideontimes }}\left(1+\frac{{\left({{{{\sf (} \! \rotatebox{146}{\sf c}}}^{\divideontimes }}^{{\mathfrak{L}}_{1}^{{ { \bar{\d{\rm L}}} }^{\diamond }}}-1\right)}^{{\mathfrak{t}}^{\ddagger }}}{{\left({{{\sf (} \! \rotatebox{146}{\sf c}}}^{\divideontimes }-1\right)}^{{\mathfrak{t}}^{\ddagger }-1}}\right)+\iota {{\text{log}}}_{{{{\sf (} \! \rotatebox{146}{\sf c}}}^{\divideontimes }}\left(1+\frac{{\left({{{{\sf (} \! \rotatebox{146}{\sf c}}}^{\divideontimes }}^{{ \frown\!\!\!\!\!\! {\smallint} }_{1}^{{ { \bar{\d{\rm L}}} }^{\diamond }}}-1\right)}^{{\mathfrak{t}}^{\ddagger }}}{{\left({{{\sf (} \! \rotatebox{146}{\sf c}}}^{\divideontimes }-1\right)}^{{\mathfrak{t}}^{\ddagger }-1}}\right)\end{array}\right)\\ & \quad \otimes \left(\begin{array}{c}{{\text{log}}}_{{{{\sf (} \! \rotatebox{146}{\sf c}}}^{\divideontimes }}\left(1+\frac{{\left({{{{\sf (} \! \rotatebox{146}{\sf c}}}^{\divideontimes }}^{{\mathfrak{L}}_{2}^{{\rotatebox{180}{{\rm h}}}^{\diamond }}}-1\right)}^{{\mathfrak{t}}^{\ddagger }}}{{\left({{{\sf (} \! \rotatebox{146}{\sf c}}}^{\divideontimes }-1\right)}^{{\mathfrak{t}}^{\ddagger }-1}}\right)+\iota {{\text{log}}}_{{{{\sf (} \! \rotatebox{146}{\sf c}}}^{\divideontimes }}\left(1+\frac{{\left({{{{\sf (} \! \rotatebox{146}{\sf c}}}^{\divideontimes }}^{{ \frown\!\!\!\!\!\! {\smallint} }_{2}^{{\rotatebox{180}{{\rm h}}}^{\diamond }}}-1\right)}^{{\mathfrak{t}}^{\ddagger }}}{{\left({{{\sf (} \! \rotatebox{146}{\sf c}}}^{\divideontimes }-1\right)}^{{\mathfrak{t}}^{\ddagger }-1}}\right),\\ {{\text{log}}}_{{{{\sf (} \! \rotatebox{146}{\sf c}}}^{\divideontimes }}\left(1+\frac{{\left({{{{\sf (} \! \rotatebox{146}{\sf c}}}^{\divideontimes }}^{{\mathfrak{L}}_{2}^{{ { \bar{\d{\rm L}}} }^{\diamond }}}-1\right)}^{{\mathfrak{t}}^{\ddagger }}}{{\left({{{\sf (} \! \rotatebox{146}{\sf c}}}^{\divideontimes }-1\right)}^{{\mathfrak{t}}^{\ddagger }-1}}\right)+\iota {{\text{log}}}_{{{{\sf (} \! \rotatebox{146}{\sf c}}}^{\divideontimes }}\left(1+\frac{{\left({{{{\sf (} \! \rotatebox{146}{\sf c}}}^{\divideontimes }}^{{ \frown\!\!\!\!\!\! {\smallint} }_{2}^{{ { \bar{\d{\rm L}}} }^{\diamond }}}-1\right)}^{{\mathfrak{t}}^{\ddagger }}}{{\left({{{\sf (} \! \rotatebox{146}{\sf c}}}^{\divideontimes }-1\right)}^{{\mathfrak{t}}^{\ddagger }-1}}\right)\end{array}\right)\end{aligned}$$
  $$={{\hat{C} }_{om.-1}}^{{\mathfrak{t}}^{\ddagger }}\otimes {{\hat{C} }_{om.-2}}^{{\mathfrak{t}}^{\ddagger }}$$
5. By using Definition 8, we get
  $${\mathfrak{t}}_{1}^{\ddagger }{\hat{C} }_{om.-1}=\left(\begin{array}{c}1-{{\text{log}}}_{{{{\sf (} \! \rotatebox{146}{\sf c}}}^{\divideontimes }}\left(1+\frac{{\left({{{{\sf (} \! \rotatebox{146}{\sf c}}}^{\divideontimes }}^{1-{\mathfrak{L}}_{1}^{{\rotatebox{180}{{\rm h}}}^{\diamond }}}-1\right)}^{{\mathfrak{t}}_{1}^{\ddagger }}}{{\left({{{\sf (} \! \rotatebox{146}{\sf c}}}^{\divideontimes }-1\right)}^{{\mathfrak{t}}_{1}^{\ddagger }-1}}\right)+\iota {1-{\text{log}}}_{{{{\sf (} \! \rotatebox{146}{\sf c}}}^{\divideontimes }}\left(1+\frac{{\left({{{{\sf (} \! \rotatebox{146}{\sf c}}}^{\divideontimes }}^{{1- \frown\!\!\!\!\!\! {\smallint} }_{1}^{{\rotatebox{180}{{\rm h}}}^{\diamond }}}-1\right)}^{{\mathfrak{t}}_{1}^{\ddagger }}}{{\left({{{\sf (} \! \rotatebox{146}{\sf c}}}^{\divideontimes }-1\right)}^{{\mathfrak{t}}^{\ddagger }-1}}\right),\\ 1-{{\text{log}}}_{{{{\sf (} \! \rotatebox{146}{\sf c}}}^{\divideontimes }}\left(1+\frac{{\left({{{{\sf (} \! \rotatebox{146}{\sf c}}}^{\divideontimes }}^{1-{\mathfrak{L}}_{1}^{{ { \bar{\d{\rm L}}} }^{\diamond }}}-1\right)}^{{\mathfrak{t}}_{1}^{\ddagger }}}{{\left({{{\sf (} \! \rotatebox{146}{\sf c}}}^{\divideontimes }-1\right)}^{{\mathfrak{t}}_{1}^{\ddagger }-1}}\right)+\iota {1-{\text{log}}}_{{{{\sf (} \! \rotatebox{146}{\sf c}}}^{\divideontimes }}\left(1+\frac{{\left({{{{\sf (} \! \rotatebox{146}{\sf c}}}^{\divideontimes }}^{{1- \frown\!\!\!\!\!\! {\smallint} }_{1}^{{ { \bar{\d{\rm L}}} }^{\diamond }}}-1\right)}^{{\mathfrak{t}}_{1}^{\ddagger }}}{{\left({{{\sf (} \! \rotatebox{146}{\sf c}}}^{\divideontimes }-1\right)}^{{\mathfrak{t}}_{1}^{\ddagger }-1}}\right)\end{array}\right)$$
  $${\mathfrak{t}}_{2}^{\ddagger }{\hat{C} }_{om.-1}=\left(\begin{array}{c}1-{{\text{log}}}_{{{{\sf (} \! \rotatebox{146}{\sf c}}}^{\divideontimes }}\left(1+\frac{{\left({{{{\sf (} \! \rotatebox{146}{\sf c}}}^{\divideontimes }}^{1-{\mathfrak{L}}_{1}^{{\rotatebox{180}{{\rm h}}}^{\diamond }}}-1\right)}^{{\mathfrak{t}}_{2}^{\ddagger }}}{{\left({{{\sf (} \! \rotatebox{146}{\sf c}}}^{\divideontimes }-1\right)}^{{\mathfrak{t}}_{2}^{\ddagger }-1}}\right)+\iota {1-{\text{log}}}_{{{{\sf (} \! \rotatebox{146}{\sf c}}}^{\divideontimes }}\left(1+\frac{{\left({{{{\sf (} \! \rotatebox{146}{\sf c}}}^{\divideontimes }}^{{1- \frown\!\!\!\!\!\! {\smallint} }_{1}^{{\rotatebox{180}{{\rm h}}}^{\diamond }}}-1\right)}^{{\mathfrak{t}}_{2}^{\ddagger }}}{{\left({{{\sf (} \! \rotatebox{146}{\sf c}}}^{\divideontimes }-1\right)}^{{\mathfrak{t}}_{2}^{\ddagger }-1}}\right),\\ 1-{{\text{log}}}_{{{{\sf (} \! \rotatebox{146}{\sf c}}}^{\divideontimes }}\left(1+\frac{{\left({{{{\sf (} \! \rotatebox{146}{\sf c}}}^{\divideontimes }}^{1-{\mathfrak{L}}_{1}^{{ { \bar{\d{\rm L}}} }^{\diamond }}}-1\right)}^{{\mathfrak{t}}_{2}^{\ddagger }}}{{\left({{{\sf (} \! \rotatebox{146}{\sf c}}}^{\divideontimes }-1\right)}^{{\mathfrak{t}}_{2}^{\ddagger }-1}}\right)+\iota {1-{\text{log}}}_{{{{\sf (} \! \rotatebox{146}{\sf c}}}^{\divideontimes }}\left(1+\frac{{\left({{{{\sf (} \! \rotatebox{146}{\sf c}}}^{\divideontimes }}^{{1- \frown\!\!\!\!\!\! {\smallint} }_{1}^{{ { \bar{\d{\rm L}}} }^{\diamond }}}-1\right)}^{{\mathfrak{t}}_{2}^{\ddagger }}}{{\left({{{\sf (} \! \rotatebox{146}{\sf c}}}^{\divideontimes }-1\right)}^{{\mathfrak{t}}_{2}^{\ddagger }-1}}\right)\end{array}\right)$$
  $$\begin{aligned} & {\mathfrak{t}}_{1}^{\ddagger }{\hat{C} }_{om.-1}\oplus {\mathfrak{t}}_{2}^{\ddagger }{\hat{C} }_{om.-1}\\ & \quad =\left(\begin{array}{c}1-{{\text{log}}}_{{{{\sf (} \! \rotatebox{146}{\sf c}}}^{\divideontimes }}\left(1+\frac{{\left({{{{\sf (} \! \rotatebox{146}{\sf c}}}^{\divideontimes }}^{1-{\mathfrak{L}}_{1}^{{\rotatebox{180}{{\rm h}}}^{\diamond }}}-1\right)}^{{\mathfrak{t}}_{1}^{\ddagger }}}{{\left({{{\sf (} \! \rotatebox{146}{\sf c}}}^{\divideontimes }-1\right)}^{{\mathfrak{t}}_{1}^{\ddagger }-1}}\right)+\iota {1-{\text{log}}}_{{{{\sf (} \! \rotatebox{146}{\sf c}}}^{\divideontimes }}\left(1+\frac{{\left({{{{\sf (} \! \rotatebox{146}{\sf c}}}^{\divideontimes }}^{{1- \frown\!\!\!\!\!\! {\smallint} }_{1}^{{\rotatebox{180}{{\rm h}}}^{\diamond }}}-1\right)}^{{\mathfrak{t}}_{1}^{\ddagger }}}{{\left({{{\sf (} \! \rotatebox{146}{\sf c}}}^{\divideontimes }-1\right)}^{{\mathfrak{t}}_{1}^{\ddagger }-1}}\right),\\ 1-{{\text{log}}}_{{{{\sf (} \! \rotatebox{146}{\sf c}}}^{\divideontimes }}\left(1+\frac{{\left({{{{\sf (} \! \rotatebox{146}{\sf c}}}^{\divideontimes }}^{1-{\mathfrak{L}}_{1}^{{ { \bar{\d{\rm L}}} }^{\diamond }}}-1\right)}^{{\mathfrak{t}}_{1}^{\ddagger }}}{{\left({{{\sf (} \! \rotatebox{146}{\sf c}}}^{\divideontimes }-1\right)}^{{\mathfrak{t}}_{1}^{\ddagger }-1}}\right)+\iota {1-{\text{log}}}_{{{{\sf (} \! \rotatebox{146}{\sf c}}}^{\divideontimes }}\left(1+\frac{{\left({{{{\sf (} \! \rotatebox{146}{\sf c}}}^{\divideontimes }}^{{1- \frown\!\!\!\!\!\! {\smallint} }_{1}^{{ { \bar{\d{\rm L}}} }^{\diamond }}}-1\right)}^{{\mathfrak{t}}_{1}^{\ddagger }}}{{\left({{{\sf (} \! \rotatebox{146}{\sf c}}}^{\divideontimes }-1\right)}^{{\mathfrak{t}}_{1}^{\ddagger }-1}}\right)\end{array}\right)\\ &\quad\oplus \left(\begin{array}{c}1-{{\text{log}}}_{{{{\sf (} \! \rotatebox{146}{\sf c}}}^{\divideontimes }}\left(1+\frac{{\left({{{{\sf (} \! \rotatebox{146}{\sf c}}}^{\divideontimes }}^{1-{\mathfrak{L}}_{1}^{{\rotatebox{180}{{\rm h}}}^{\diamond }}}-1\right)}^{{\mathfrak{t}}_{2}^{\ddagger }}}{{\left({{{\sf (} \! \rotatebox{146}{\sf c}}}^{\divideontimes }-1\right)}^{{\mathfrak{t}}_{2}^{\ddagger }-1}}\right)+\iota {1-{\text{log}}}_{{{{\sf (} \! \rotatebox{146}{\sf c}}}^{\divideontimes }}\left(1+\frac{{\left({{{{\sf (} \! \rotatebox{146}{\sf c}}}^{\divideontimes }}^{{1- \frown\!\!\!\!\!\! {\smallint} }_{1}^{{\rotatebox{180}{{\rm h}}}^{\diamond }}}-1\right)}^{{\mathfrak{t}}_{2}^{\ddagger }}}{{\left({{{\sf (} \! \rotatebox{146}{\sf c}}}^{\divideontimes }-1\right)}^{{\mathfrak{t}}_{2}^{\ddagger }-1}}\right),\\ 1-{{\text{log}}}_{{{{\sf (} \! \rotatebox{146}{\sf c}}}^{\divideontimes }}\left(1+\frac{{\left({{{{\sf (} \! \rotatebox{146}{\sf c}}}^{\divideontimes }}^{1-{\mathfrak{L}}_{1}^{{ { \bar{\d{\rm L}}} }^{\diamond }}}-1\right)}^{{\mathfrak{t}}_{2}^{\ddagger }}}{{\left({{{\sf (} \! \rotatebox{146}{\sf c}}}^{\divideontimes }-1\right)}^{{\mathfrak{t}}_{2}^{\ddagger }-1}}\right)+\iota {1-{\text{log}}}_{{{{\sf (} \! \rotatebox{146}{\sf c}}}^{\divideontimes }}\left(1+\frac{{\left({{{{\sf (} \! \rotatebox{146}{\sf c}}}^{\divideontimes }}^{{1- \frown\!\!\!\!\!\! {\smallint} }_{1}^{{ { \bar{\d{\rm L}}} }^{\diamond }}}-1\right)}^{{\mathfrak{t}}_{2}^{\ddagger }}}{{\left({{{\sf (} \! \rotatebox{146}{\sf c}}}^{\divideontimes }-1\right)}^{{\mathfrak{t}}_{2}^{\ddagger }-1}}\right)\end{array}\right)\end{aligned}$$
  $$=\left(\begin{array}{c}1-{{\text{log}}}_{{{{\sf (} \! \rotatebox{146}{\sf c}}}^{\divideontimes }}\left(1+\frac{{\left({{{{\sf (} \! \rotatebox{146}{\sf c}}}^{\divideontimes }}^{1-{\mathfrak{L}}_{1}^{{\rotatebox{180}{{\rm h}}}^{\diamond }}}-1\right)}^{{\mathfrak{t}}_{1}^{\ddagger }+{\mathfrak{t}}_{2}^{\ddagger }}}{{\left({{{\sf (} \! \rotatebox{146}{\sf c}}}^{\divideontimes }-1\right)}^{{\mathfrak{t}}_{1}^{\ddagger }+{\mathfrak{t}}_{2}^{\ddagger }}}\right)+\iota {1-{\text{log}}}_{{{{\sf (} \! \rotatebox{146}{\sf c}}}^{\divideontimes }}\left(1+\frac{{\left({{{{\sf (} \! \rotatebox{146}{\sf c}}}^{\divideontimes }}^{{1- \frown\!\!\!\!\!\! {\smallint} }_{1}^{{\rotatebox{180}{{\rm h}}}^{\diamond }}}-1\right)}^{{\mathfrak{t}}_{1}^{\ddagger }+{\mathfrak{t}}_{2}^{\ddagger }}}{{\left({{{\sf (} \! \rotatebox{146}{\sf c}}}^{\divideontimes }-1\right)}^{{\mathfrak{t}}_{1}^{\ddagger }+{\mathfrak{t}}_{2}^{\ddagger }}}\right),\\ 1-{{\text{log}}}_{{{{\sf (} \! \rotatebox{146}{\sf c}}}^{\divideontimes }}\left(1+\frac{{\left({{{{\sf (} \! \rotatebox{146}{\sf c}}}^{\divideontimes }}^{1-{\mathfrak{L}}_{1}^{{ { \bar{\d{\rm L}}} }^{\diamond }}}-1\right)}^{{\mathfrak{t}}_{1}^{\ddagger }{\mathfrak{t}}_{2}^{\ddagger }}}{{\left({{{\sf (} \! \rotatebox{146}{\sf c}}}^{\divideontimes }-1\right)}^{{\mathfrak{t}}_{1}^{\ddagger }+{\mathfrak{t}}_{2}^{\ddagger }}}\right)+\iota {1-{\text{log}}}_{{{{\sf (} \! \rotatebox{146}{\sf c}}}^{\divideontimes }}\left(1+\frac{{\left({{{{\sf (} \! \rotatebox{146}{\sf c}}}^{\divideontimes }}^{{1- \frown\!\!\!\!\!\! {\smallint} }_{1}^{{ { \bar{\d{\rm L}}} }^{\diamond }}}-1\right)}^{{\mathfrak{t}}_{1}^{\ddagger }+{\mathfrak{t}}_{2}^{\ddagger }}}{{\left({{{\sf (} \! \rotatebox{146}{\sf c}}}^{\divideontimes }-1\right)}^{{\mathfrak{t}}_{1}^{\ddagger }+{\mathfrak{t}}_{2}^{\ddagger }}}\right)\end{array}\right)$$
  $$=\left({\mathfrak{t}}_{1}^{\ddagger }+{\mathfrak{t}}_{2}^{\ddagger }\right){\hat{C} }_{om.-1}$$
6. By utilization of Definition 8, we get
  $${\left({\hat{C} }_{om.-1}\right)}^{{\mathfrak{t}}_{1}^{\ddagger }}=\left(\begin{array}{c}{{\text{log}}}_{{{{\sf (} \! \rotatebox{146}{\sf c}}}^{\divideontimes }}\left(1+\frac{{\left({{{{\sf (} \! \rotatebox{146}{\sf c}}}^{\divideontimes }}^{{\mathfrak{L}}_{1}^{{\rotatebox{180}{{\rm h}}}^{\diamond }}}-1\right)}^{{\mathfrak{t}}_{1}^{\ddagger }}}{{\left({{{\sf (} \! \rotatebox{146}{\sf c}}}^{\divideontimes }-1\right)}^{{\mathfrak{t}}_{1}^{\ddagger }-1}}\right)+\iota {{\text{log}}}_{{{{\sf (} \! \rotatebox{146}{\sf c}}}^{\divideontimes }}\left(1+\frac{{\left({{{{\sf (} \! \rotatebox{146}{\sf c}}}^{\divideontimes }}^{{ \frown\!\!\!\!\!\! {\smallint} }_{1}^{{\rotatebox{180}{{\rm h}}}^{\diamond }}}-1\right)}^{{\mathfrak{t}}_{1}^{\ddagger }}}{{\left({{{\sf (} \! \rotatebox{146}{\sf c}}}^{\divideontimes }-1\right)}^{{\mathfrak{t}}_{1}^{\ddagger }-1}}\right),\\ {{\text{log}}}_{{{{\sf (} \! \rotatebox{146}{\sf c}}}^{\divideontimes }}\left(1+\frac{{\left({{{{\sf (} \! \rotatebox{146}{\sf c}}}^{\divideontimes }}^{{\mathfrak{L}}_{1}^{{ { \bar{\d{\rm L}}} }^{\diamond }}}-1\right)}^{{\mathfrak{t}}_{1}^{\ddagger }}}{{\left({{{\sf (} \! \rotatebox{146}{\sf c}}}^{\divideontimes }-1\right)}^{{\mathfrak{t}}_{1}^{\ddagger }-1}}\right)+\iota {{\text{log}}}_{{{{\sf (} \! \rotatebox{146}{\sf c}}}^{\divideontimes }}\left(1+\frac{{\left({{{{\sf (} \! \rotatebox{146}{\sf c}}}^{\divideontimes }}^{{ \frown\!\!\!\!\!\! {\smallint} }_{1}^{{ { \bar{\d{\rm L}}} }^{\diamond }}}-1\right)}^{{\mathfrak{t}}_{1}^{\ddagger }}}{{\left({{{\sf (} \! \rotatebox{146}{\sf c}}}^{\divideontimes }-1\right)}^{{\mathfrak{t}}_{1}^{\ddagger }-1}}\right)\end{array}\right)$$
  $${\left({\hat{C} }_{om.-1}\right)}^{{\mathfrak{t}}_{2}^{\ddagger }}=\left(\begin{array}{c}{{\text{log}}}_{{{{\sf (} \! \rotatebox{146}{\sf c}}}^{\divideontimes }}\left(1+\frac{{\left({{{{\sf (} \! \rotatebox{146}{\sf c}}}^{\divideontimes }}^{{\mathfrak{L}}_{1}^{{\rotatebox{180}{{\rm h}}}^{\diamond }}}-1\right)}^{{\mathfrak{t}}_{2}^{\ddagger }}}{{\left({{{\sf (} \! \rotatebox{146}{\sf c}}}^{\divideontimes }-1\right)}^{{\mathfrak{t}}_{2}^{\ddagger }-1}}\right)+\iota {{\text{log}}}_{{{{\sf (} \! \rotatebox{146}{\sf c}}}^{\divideontimes }}\left(1+\frac{{\left({{{{\sf (} \! \rotatebox{146}{\sf c}}}^{\divideontimes }}^{{ \frown\!\!\!\!\!\! {\smallint} }_{1}^{{\rotatebox{180}{{\rm h}}}^{\diamond }}}-1\right)}^{{\mathfrak{t}}_{2}^{\ddagger }}}{{\left({{{\sf (} \! \rotatebox{146}{\sf c}}}^{\divideontimes }-1\right)}^{{\mathfrak{t}}_{2}^{\ddagger }-1}}\right),\\ {{\text{log}}}_{{{{\sf (} \! \rotatebox{146}{\sf c}}}^{\divideontimes }}\left(1+\frac{{\left({{{{\sf (} \! \rotatebox{146}{\sf c}}}^{\divideontimes }}^{{\mathfrak{L}}_{1}^{{ { \bar{\d{\rm L}}} }^{\diamond }}}-1\right)}^{{\mathfrak{t}}_{2}^{\ddagger }}}{{\left({{{\sf (} \! \rotatebox{146}{\sf c}}}^{\divideontimes }-1\right)}^{{\mathfrak{t}}_{2}^{\ddagger }-1}}\right)+\iota {{\text{log}}}_{{{{\sf (} \! \rotatebox{146}{\sf c}}}^{\divideontimes }}\left(1+\frac{{\left({{{{\sf (} \! \rotatebox{146}{\sf c}}}^{\divideontimes }}^{{ \frown\!\!\!\!\!\! {\smallint} }_{1}^{{ { \bar{\d{\rm L}}} }^{\diamond }}}-1\right)}^{{\mathfrak{t}}_{2}^{\ddagger }}}{{\left({{{\sf (} \! \rotatebox{146}{\sf c}}}^{\divideontimes }-1\right)}^{{\mathfrak{t}}_{2}^{\ddagger }-1}}\right)\end{array}\right)$$
  $$\begin{aligned} & {\left({\hat{C} }_{om.-1}\right)}^{{\mathfrak{t}}_{1}^{\ddagger }}\otimes {\left({\hat{C} }_{om.-1}\right)}^{{\mathfrak{t}}_{2}^{\ddagger }}\\ & \quad = \left(\begin{array}{c}{{\text{log}}}_{{{{\sf (} \! \rotatebox{146}{\sf c}}}^{\divideontimes }}\left(1+\frac{{\left({{{{\sf (} \! \rotatebox{146}{\sf c}}}^{\divideontimes }}^{{\mathfrak{L}}_{1}^{{\rotatebox{180}{{\rm h}}}^{\diamond }}}-1\right)}^{{\mathfrak{t}}_{1}^{\ddagger }}}{{\left({{{\sf (} \! \rotatebox{146}{\sf c}}}^{\divideontimes }-1\right)}^{{\mathfrak{t}}_{1}^{\ddagger }-1}}\right)+\iota {{\text{log}}}_{{{{\sf (} \! \rotatebox{146}{\sf c}}}^{\divideontimes }}\left(1+\frac{{\left({{{{\sf (} \! \rotatebox{146}{\sf c}}}^{\divideontimes }}^{{ \frown\!\!\!\!\!\! {\smallint} }_{1}^{{\rotatebox{180}{{\rm h}}}^{\diamond }}}-1\right)}^{{\mathfrak{t}}_{1}^{\ddagger }}}{{\left({{{\sf (} \! \rotatebox{146}{\sf c}}}^{\divideontimes }-1\right)}^{{\mathfrak{t}}_{1}^{\ddagger }-1}}\right),\\ {{\text{log}}}_{{{{\sf (} \! \rotatebox{146}{\sf c}}}^{\divideontimes }}\left(1+\frac{{\left({{{{\sf (} \! \rotatebox{146}{\sf c}}}^{\divideontimes }}^{{\mathfrak{L}}_{1}^{{ { \bar{\d{\rm L}}} }^{\diamond }}}-1\right)}^{{\mathfrak{t}}_{1}^{\ddagger }}}{{\left({{{\sf (} \! \rotatebox{146}{\sf c}}}^{\divideontimes }-1\right)}^{{\mathfrak{t}}_{1}^{\ddagger }-1}}\right)+\iota {{\text{log}}}_{{{{\sf (} \! \rotatebox{146}{\sf c}}}^{\divideontimes }}\left(1+\frac{{\left({{{{\sf (} \! \rotatebox{146}{\sf c}}}^{\divideontimes }}^{{ \frown\!\!\!\!\!\! {\smallint} }_{1}^{{ { \bar{\d{\rm L}}} }^{\diamond }}}-1\right)}^{{\mathfrak{t}}_{1}^{\ddagger }}}{{\left({{{\sf (} \! \rotatebox{146}{\sf c}}}^{\divideontimes }-1\right)}^{{\mathfrak{t}}_{1}^{\ddagger }-1}}\right)\end{array}\right)\\ &\quad\otimes \left(\begin{array}{c}{{\text{log}}}_{{{{\sf (} \! \rotatebox{146}{\sf c}}}^{\divideontimes }}\left(1+\frac{{\left({{{{\sf (} \! \rotatebox{146}{\sf c}}}^{\divideontimes }}^{{\mathfrak{L}}_{1}^{{\rotatebox{180}{{\rm h}}}^{\diamond }}}-1\right)}^{{\mathfrak{t}}_{2}^{\ddagger }}}{{\left({{{\sf (} \! \rotatebox{146}{\sf c}}}^{\divideontimes }-1\right)}^{{\mathfrak{t}}_{1}^{\ddagger }-1}}\right)+\iota {{\text{log}}}_{{{{\sf (} \! \rotatebox{146}{\sf c}}}^{\divideontimes }}\left(1+\frac{{\left({{{{\sf (} \! \rotatebox{146}{\sf c}}}^{\divideontimes }}^{{ \frown\!\!\!\!\!\! {\smallint} }_{1}^{{\rotatebox{180}{{\rm h}}}^{\diamond }}}-1\right)}^{{\mathfrak{t}}_{2}^{\ddagger }}}{{\left({{{\sf (} \! \rotatebox{146}{\sf c}}}^{\divideontimes }-1\right)}^{{\mathfrak{t}}_{1}^{\ddagger }-1}}\right),\\ {{\text{log}}}_{{{{\sf (} \! \rotatebox{146}{\sf c}}}^{\divideontimes }}\left(1+\frac{{\left({{{{\sf (} \! \rotatebox{146}{\sf c}}}^{\divideontimes }}^{{\mathfrak{L}}_{1}^{{ { \bar{\d{\rm L}}} }^{\diamond }}}-1\right)}^{{\mathfrak{t}}_{2}^{\ddagger }}}{{\left({{{\sf (} \! \rotatebox{146}{\sf c}}}^{\divideontimes }-1\right)}^{{\mathfrak{t}}_{2}^{\ddagger }-1}}\right)+\iota {{\text{log}}}_{{{{\sf (} \! \rotatebox{146}{\sf c}}}^{\divideontimes }}\left(1+\frac{{\left({{{{\sf (} \! \rotatebox{146}{\sf c}}}^{\divideontimes }}^{{ \frown\!\!\!\!\!\! {\smallint} }_{1}^{{ { \bar{\d{\rm L}}} }^{\diamond }}}-1\right)}^{{\mathfrak{t}}_{2}^{\ddagger }}}{{\left({{{\sf (} \! \rotatebox{146}{\sf c}}}^{\divideontimes }-1\right)}^{{\mathfrak{t}}_{2}^{\ddagger }-1}}\right)\end{array}\right)\end{aligned}$$
  $$=\left(\begin{array}{c}{{\text{log}}}_{{{{\sf (} \! \rotatebox{146}{\sf c}}}^{\divideontimes }}\left(1+\frac{{\left({{{{\sf (} \! \rotatebox{146}{\sf c}}}^{\divideontimes }}^{{\mathfrak{L}}_{1}^{{\rotatebox{180}{{\rm h}}}^{\diamond }}}-1\right)}^{{\mathfrak{t}}_{1}^{\ddagger }+{\mathfrak{t}}_{2}^{\ddagger }}}{{\left({{{\sf (} \! \rotatebox{146}{\sf c}}}^{\divideontimes }-1\right)}^{{\mathfrak{t}}_{1}^{\ddagger }+{\mathfrak{t}}_{2}^{\ddagger }-1}}\right)+\iota {{\text{log}}}_{{{{\sf (} \! \rotatebox{146}{\sf c}}}^{\divideontimes }}\left(1+\frac{{\left({{{{\sf (} \! \rotatebox{146}{\sf c}}}^{\divideontimes }}^{{ \frown\!\!\!\!\!\! {\smallint} }_{1}^{{\rotatebox{180}{{\rm h}}}^{\diamond }}}-1\right)}^{{\mathfrak{t}}_{1}^{\ddagger }+{\mathfrak{t}}_{2}^{\ddagger }}}{{\left({{{\sf (} \! \rotatebox{146}{\sf c}}}^{\divideontimes }-1\right)}^{{\mathfrak{t}}_{1}^{\ddagger }+{\mathfrak{t}}_{2}^{\ddagger }-1}}\right),\\ {{\text{log}}}_{{{{\sf (} \! \rotatebox{146}{\sf c}}}^{\divideontimes }}\left(1+\frac{{\left({{{{\sf (} \! \rotatebox{146}{\sf c}}}^{\divideontimes }}^{{\mathfrak{L}}_{1}^{{ { \bar{\d{\rm L}}} }^{\diamond }}}-1\right)}^{{\mathfrak{t}}_{1}^{\ddagger }+{\mathfrak{t}}_{2}^{\ddagger }}}{{\left({{{\sf (} \! \rotatebox{146}{\sf c}}}^{\divideontimes }-1\right)}^{{\mathfrak{t}}_{1}^{\ddagger }+{\mathfrak{t}}_{2}^{\ddagger }-1}}\right)+\iota {{\text{log}}}_{{{{\sf (} \! \rotatebox{146}{\sf c}}}^{\divideontimes }}\left(1+\frac{{\left({{{{\sf (} \! \rotatebox{146}{\sf c}}}^{\divideontimes }}^{{ \frown\!\!\!\!\!\! {\smallint} }_{1}^{{ { \bar{\d{\rm L}}} }^{\diamond }}}-1\right)}^{{\mathfrak{t}}_{1}^{\ddagger }+{\mathfrak{t}}_{2}^{\ddagger }}}{{\left({{{\sf (} \! \rotatebox{146}{\sf c}}}^{\divideontimes }-1\right)}^{{\mathfrak{t}}_{1}^{\ddagger }+{\mathfrak{t}}_{2}^{\ddagger }-1}}\right)\end{array}\right)$$
  $$={\left({\hat{C} }_{om.-1}\right)}^{{\mathfrak{t}}_{1}^{\ddagger }+{\mathfrak{t}}_{2}^{\ddagger }}$$
7. It is obvious.

## Complex fuzzy rough Frank (CFRF) Aggregation operators (AOs)

In this section, we have to discuss the notion of AOs called CFRF AOs. Now throughout this section,

${\hat{C} }_{om.-j}=\left({\mathfrak{L}}_{j}^{{\rotatebox{180}{{\rm h}}}^{\diamond }}+\iota { \frown\!\!\!\!\!\! {\smallint} }_{j}^{{\rotatebox{180}{{\rm h}}}^{\diamond }}, {\mathfrak{L}}_{j}^{{ { \bar{\d{\rm L}}} }^{\diamond }}+\iota { \frown\!\!\!\!\!\! {\smallint} }_{j}^{{ { \bar{\d{\rm L}}} }^{\diamond }}\right) \left(j=1, 2, .., n\right)$ be a collection of CFRNs. Also, assume that ${\Omega }_{\mathbbm{w}}=\left({\Omega }_{{\mathbbm{w}}-1}, {\Omega }_{{\mathbbm{w}}-2}\dots , {\Omega }_{{\mathbbm{w}}-n}\right)$ denote the weight vector (WV) such that $0\le {\Omega }_{{\mathbbm{w}}-j}\le 1$, and $\sum_{j=1}^{n}{\Omega }_{{\mathbbm{w}}-j}=1.$

### Complex fuzzy rough Frank arithmetic AOs

This part of the article is devoted to defining the notion of CFRF arithmetic AOs. The overall discussion is given by:

#### Definition 10

For the family of CFRNs, the CFR Frank weighted average (CFRFWA) AO is the function ${\left({\hat{C} }_{om.}\right)}^{n}\to \left({\hat{C} }_{om.}\right)$ given by

1 $$CFRFWA \left({\hat{\text{C}} }_{om.-1}, {\hat{\text{C}} }_{om.-2}, \dots , {\hat{\text{C}} }_{om.-n}\right)=\begin{array}{c}n\\ \oplus \\ j=1\end{array}{\Omega }_{{\mathbbm{w}}-j}{\hat{\text{C}} }_{om.-j}$$

#### Theorem 2

*Let* ${\hat{C} }_{om.-j}=\left({\mathfrak{L}}_{j}^{{\rotatebox{180}{{\rm h}}}^{\diamond }}+\iota { \frown\!\!\!\!\!\! {\smallint} }_{j}^{{\rotatebox{180}{{\rm h}}}^{\diamond }}, {\mathfrak{L}}_{j}^{{ { \bar{\d{\rm L}}} }^{\diamond }}+\iota { \frown\!\!\!\!\!\! {\smallint} }_{j}^{{ { \bar{\d{\rm L}}} }^{\diamond }}\right) \left(j=1, 2, .., n\right)$ *be a collection of CFRNs, the aggregating result by using* Eq. (1) is again CFRN given as

2 $$\begin{aligned} & CFRFWA\left({\hat{\text{C}} }_{om.-1}, {\hat{\text{C}} }_{om.-2}, \dots , {\hat{\text{C}} }_{om.-n}\right)\\ & \quad =\left(\begin{array}{c}1-\left({{\text{log}}}_{{{{{{\sf (} \! \rotatebox{146}{\sf c}}}}}^{\divideontimes }}\left(1+\prod_{j=1}^{n}{\left({{{{{{\sf (} \! \rotatebox{146}{\sf c}}}}}^{\divideontimes }}^{1-{\mathfrak{L}}_{j}^{{{{\rotatebox{180}{{\rm h}}}}}^{\diamond }}}-1\right)}^{{\Omega }_{{\mathbbm{w}}-j}}\right)\right)+\iota 1-\left({{\text{log}}}_{{{{{{\sf (} \! \rotatebox{146}{\sf c}}}}}^{\divideontimes }}\left(1+\prod_{j=1}^{n}{\left({{{{{{\sf (} \! \rotatebox{146}{\sf c}}}}}^{\divideontimes }}^{1-{ \frown\!\!\!\!\!\! {\smallint} }_{j}^{{{{\rotatebox{180}{{\rm h}}}}}^{\diamond }}}-1\right)}^{{\Omega }_{{\mathbbm{w}}-j}}\right)\right),\\ 1-\left({{\text{log}}}_{{{{{{\sf (} \! \rotatebox{146}{\sf c}}}}}^{\divideontimes }}\left(1+\prod_{j=1}^{n}{\left({{{{{{\sf (} \! \rotatebox{146}{\sf c}}}}}^{\divideontimes }}^{1-{\mathfrak{L}}_{j}^{{{{ { \bar{\d{\rm L}}} }}}^{\diamond }}}-1\right)}^{{\Omega }_{{\mathbbm{w}}-j}}\right)\right)+\iota 1-\left({{\text{log}}}_{{{{{{\sf (} \! \rotatebox{146}{\sf c}}}}}^{\divideontimes }}\left(1+\prod_{j=1}^{n}{\left({{{{{{\sf (} \! \rotatebox{146}{\sf c}}}}}^{\divideontimes }}^{1-{ \frown\!\!\!\!\!\! {\smallint} }_{j}^{{{{ { \bar{\d{\rm L}}} }}}^{\diamond }}}-1\right)}^{{\Omega }_{{\mathbbm{w}}-j}}\right)\right),\end{array}\right) \end{aligned}$$

#### Proof

 Using the method of mathematical induction, for $n=2$, we get

$$CFRFWA\left({\hat{C} }_{om.-1}, {\hat{C} }_{om.-2}\right)={\Omega }_{{\mathbbm{w}}-1}{\hat{C} }_{om.-1}\oplus {\Omega }_{{\mathbbm{w}}-2}{\hat{C} }_{om.-2}$$

$$=\left(\begin{array}{c}1-{{\text{log}}}_{{{{\sf (} \! \rotatebox{146}{\sf c}}}^{\divideontimes }}\left(1+\frac{{\left({{{{\sf (} \! \rotatebox{146}{\sf c}}}^{\divideontimes }}^{1-{\mathfrak{L}}_{1}^{{\rotatebox{180}{{\rm h}}}^{\diamond }}}-1\right)}^{{\Omega }_{{\mathbbm{w}}-1}}}{{\left({{{\sf (} \! \rotatebox{146}{\sf c}}}^{\divideontimes }-1\right)}^{{\Omega }_{{\mathbbm{w}}-1}-1}}\right)+\iota {1-{\text{log}}}_{{{{\sf (} \! \rotatebox{146}{\sf c}}}^{\divideontimes }}\left(1+\frac{{\left({{{{\sf (} \! \rotatebox{146}{\sf c}}}^{\divideontimes }}^{{1- \frown\!\!\!\!\!\! {\smallint} }_{1}^{{\rotatebox{180}{{\rm h}}}^{\diamond }}}-1\right)}^{{\Omega }_{{\mathbbm{w}}-1}}}{{\left({{{\sf (} \! \rotatebox{146}{\sf c}}}^{\divideontimes }-1\right)}^{{\Omega }_{{\mathbbm{w}}-1}-1}}\right),\\ 1-{{\text{log}}}_{{{{\sf (} \! \rotatebox{146}{\sf c}}}^{\divideontimes }}\left(1+\frac{{\left({{{{\sf (} \! \rotatebox{146}{\sf c}}}^{\divideontimes }}^{1-{\mathfrak{L}}_{1}^{{ { \bar{\d{\rm L}}} }^{\diamond }}}-1\right)}^{{\Omega }_{{\mathbbm{w}}-1}}}{{\left({{{\sf (} \! \rotatebox{146}{\sf c}}}^{\divideontimes }-1\right)}^{{\Omega }_{{\mathbbm{w}}-1}-1}}\right)+\iota {1-{\text{log}}}_{{{{\sf (} \! \rotatebox{146}{\sf c}}}^{\divideontimes }}\left(1+\frac{{\left({{{{\sf (} \! \rotatebox{146}{\sf c}}}^{\divideontimes }}^{{1- \frown\!\!\!\!\!\! {\smallint} }_{1}^{{ { \bar{\d{\rm L}}} }^{\diamond }}}-1\right)}^{{\Omega }_{{\mathbbm{w}}-1}}}{{\left({{{\sf (} \! \rotatebox{146}{\sf c}}}^{\divideontimes }-1\right)}^{{\Omega }_{{\mathbbm{w}}-1}-1}}\right)\end{array}\right)$$

$$\oplus \left(\begin{array}{c}1-{{\text{log}}}_{{{{\sf (} \! \rotatebox{146}{\sf c}}}^{\divideontimes }}\left(1+\frac{{\left({{{{\sf (} \! \rotatebox{146}{\sf c}}}^{\divideontimes }}^{1-{\mathfrak{L}}_{2}^{{\rotatebox{180}{{\rm h}}}^{\diamond }}}-1\right)}^{{\Omega }_{{\mathbbm{w}}-2}}}{{\left({{{\sf (} \! \rotatebox{146}{\sf c}}}^{\divideontimes }-1\right)}^{{\Omega }_{{\mathbbm{w}}-2}-1}}\right)+\iota {1-{\text{log}}}_{{{{\sf (} \! \rotatebox{146}{\sf c}}}^{\divideontimes }}\left(1+\frac{{\left({{{{\sf (} \! \rotatebox{146}{\sf c}}}^{\divideontimes }}^{{1- \frown\!\!\!\!\!\! {\smallint} }_{2}^{{\rotatebox{180}{{\rm h}}}^{\diamond }}}-1\right)}^{{\Omega }_{{\mathbbm{w}}-2}}}{{\left({{{\sf (} \! \rotatebox{146}{\sf c}}}^{\divideontimes }-1\right)}^{{\Omega }_{{\mathbbm{w}}-2}-1}}\right),\\ 1-{{\text{log}}}_{{{{\sf (} \! \rotatebox{146}{\sf c}}}^{\divideontimes }}\left(1+\frac{{\left({{{{\sf (} \! \rotatebox{146}{\sf c}}}^{\divideontimes }}^{1-{\mathfrak{L}}_{2}^{{ { \bar{\d{\rm L}}} }^{\diamond }}}-1\right)}^{{\Omega }_{{\mathbbm{w}}-2}}}{{\left({{{\sf (} \! \rotatebox{146}{\sf c}}}^{\divideontimes }-1\right)}^{{\Omega }_{{\mathbbm{w}}-2}-1}}\right)+\iota {1-{\text{log}}}_{{{{\sf (} \! \rotatebox{146}{\sf c}}}^{\divideontimes }}\left(1+\frac{{\left({{{{\sf (} \! \rotatebox{146}{\sf c}}}^{\divideontimes }}^{{1- \frown\!\!\!\!\!\! {\smallint} }_{2}^{{ { \bar{\d{\rm L}}} }^{\diamond }}}-1\right)}^{{\Omega }_{{\mathbbm{w}}-2}}}{{\left({{{\sf (} \! \rotatebox{146}{\sf c}}}^{\divideontimes }-1\right)}^{{\Omega }_{{\mathbbm{w}}-2}-1}}\right)\end{array}\right)$$

$$=\left(\begin{array}{c}1-{{\text{log}}}_{{{{\sf (} \! \rotatebox{146}{\sf c}}}^{\divideontimes }}\left(1+\frac{\prod_{j=1}^{2}{\left({{{{\sf (} \! \rotatebox{146}{\sf c}}}^{\divideontimes }}^{1-{\mathfrak{L}}_{j}^{{\rotatebox{180}{{\rm h}}}^{\diamond }}}-1\right)}^{{\Omega }_{{\mathbbm{w}}-j}}}{{\left({{{\sf (} \! \rotatebox{146}{\sf c}}}^{\divideontimes }-1\right)}^{\sum_{j=1}^{2}{\Omega }_{{\mathbbm{w}}-j}-1}}\right)+\iota 1-{{\text{log}}}_{{{{\sf (} \! \rotatebox{146}{\sf c}}}^{\divideontimes }}\left(1+\frac{\prod_{j=1}^{2}{\left({{{{\sf (} \! \rotatebox{146}{\sf c}}}^{\divideontimes }}^{1-{ \frown\!\!\!\!\!\! {\smallint} }_{j}^{{\rotatebox{180}{{\rm h}}}^{\diamond }}}-1\right)}^{{\Omega }_{{\mathbbm{w}}-j}}}{{\left({{{\sf (} \! \rotatebox{146}{\sf c}}}^{\divideontimes }-1\right)}^{\sum_{j=1}^{2}{\Omega }_{{\mathbbm{w}}-j}-1}}\right),\\ 1-{{\text{log}}}_{{{{\sf (} \! \rotatebox{146}{\sf c}}}^{\divideontimes }}\left(1+\frac{\prod_{j=1}^{2}{\left({{{{\sf (} \! \rotatebox{146}{\sf c}}}^{\divideontimes }}^{1-{\mathfrak{L}}_{j}^{{ { \bar{\d{\rm L}}} }^{\diamond }}}-1\right)}^{{\Omega }_{{\mathbbm{w}}-j}}}{{\left({{{\sf (} \! \rotatebox{146}{\sf c}}}^{\divideontimes }-1\right)}^{\sum_{j=1}^{2}{\Omega }_{{\mathbbm{w}}-j}-1}}\right)+\iota 1-{{\text{log}}}_{{{{\sf (} \! \rotatebox{146}{\sf c}}}^{\divideontimes }}\left(1+\frac{\prod_{j=1}^{2}{\left({{{{\sf (} \! \rotatebox{146}{\sf c}}}^{\divideontimes }}^{1-{ \frown\!\!\!\!\!\! {\smallint} }_{j}^{{ { \bar{\d{\rm L}}} }^{\diamond }}}-1\right)}^{{\Omega }_{{\mathbbm{w}}-j}}}{{\left({{{\sf (} \! \rotatebox{146}{\sf c}}}^{\divideontimes }-1\right)}^{\sum_{j=1}^{2}{\Omega }_{{\mathbbm{w}}-j}-1}}\right)\end{array}\right)$$

Hence, Eq. (2) is true for $n=2$.

Now suppose Eq. (2) holds for some ${\mathcalligra{k}}$ i.e.

$$\begin{aligned} & CFRFWA\left({\hat{C} }_{om.-1}, {\hat{C} }_{om.-2}, \dots , {\hat{C} }_{om.-{\mathcalligra{k}}}\right)\\ &\quad=\left(\begin{array}{c}1-{{\text{log}}}_{{{{\sf (} \! \rotatebox{146}{\sf c}}}^{\divideontimes }}\left(1+\frac{\prod_{j=1}^{{\mathcalligra{k}}}{\left({{{{\sf (} \! \rotatebox{146}{\sf c}}}^{\divideontimes }}^{1-{\mathfrak{L}}_{j}^{{\rotatebox{180}{{\rm h}}}^{\diamond }}}-1\right)}^{{\Omega }_{{\mathbbm{w}}-j}}}{{\left({{{\sf (} \! \rotatebox{146}{\sf c}}}^{\divideontimes }-1\right)}^{\sum_{j=1}^{{\mathcalligra{k}}}{\Omega }_{{\mathbbm{w}}-j}-1}}\right)+\iota 1-{{\text{log}}}_{{{{\sf (} \! \rotatebox{146}{\sf c}}}^{\divideontimes }}\left(1+\frac{\prod_{j=1}^{{\mathcalligra{k}}}{\left({{{{\sf (} \! \rotatebox{146}{\sf c}}}^{\divideontimes }}^{1-{ \frown\!\!\!\!\!\! {\smallint} }_{j}^{{\rotatebox{180}{{\rm h}}}^{\diamond }}}-1\right)}^{{\Omega }_{{\mathbbm{w}}-j}}}{{\left({{{\sf (} \! \rotatebox{146}{\sf c}}}^{\divideontimes }-1\right)}^{\sum_{j=1}^{{\mathcalligra{k}}}{\Omega }_{{\mathbbm{w}}-j}-1}}\right),\\ 1-{{\text{log}}}_{{{{\sf (} \! \rotatebox{146}{\sf c}}}^{\divideontimes }}\left(1+\frac{\prod_{j=1}^{{\mathcalligra{k}}}{\left({{{{\sf (} \! \rotatebox{146}{\sf c}}}^{\divideontimes }}^{1-{\mathfrak{L}}_{j}^{{ { \bar{\d{\rm L}}} }^{\diamond }}}-1\right)}^{{\Omega }_{{\mathbbm{w}}-j}}}{{\left({{{\sf (} \! \rotatebox{146}{\sf c}}}^{\divideontimes }-1\right)}^{\sum_{j=1}^{{\mathcalligra{k}}}{\Omega }_{{\mathbbm{w}}-j}-1}}\right)+\iota 1-{{\text{log}}}_{{{{\sf (} \! \rotatebox{146}{\sf c}}}^{\divideontimes }}\left(1+\frac{\prod_{j=1}^{{\mathcalligra{k}}}{\left({{{{\sf (} \! \rotatebox{146}{\sf c}}}^{\divideontimes }}^{1-{ \frown\!\!\!\!\!\! {\smallint} }_{j}^{{ { \bar{\d{\rm L}}} }^{\diamond }}}-1\right)}^{{\Omega }_{{\mathbbm{w}}-j}}}{{\left({{{\sf (} \! \rotatebox{146}{\sf c}}}^{\divideontimes }-1\right)}^{\sum_{j=1}^{{\mathcalligra{k}}}{\Omega }_{{\mathbbm{w}}-j}-1}}\right)\end{array}\right)\end{aligned}$$

Now we prove that Eq. (2) holds for $n={\mathcalligra{k}}+1$.

$$CFRFWA\left({\hat{C} }_{om.-1}, {\hat{C} }_{om.-2}, \dots , {\hat{C} }_{om.-{\mathcalligra{k}}}, {\hat{C} }_{om.-{\mathcalligra{k}}+1}\right)=CFRFWA\left({\hat{C} }_{om.-1}, {\hat{C} }_{om.-2}, \dots , {\hat{C} }_{om.-{\mathcalligra{k}}}\right)\oplus {\Omega }_{{\mathbbm{w}}-{\mathcalligra{k}}+1}{\hat{C} }_{om.-{\mathcalligra{k}}+1}$$

$$=\left(\begin{array}{c}1-{{\text{log}}}_{{{{\sf (} \! \rotatebox{146}{\sf c}}}^{\divideontimes }}\left(1+\frac{\prod_{j=1}^{{\mathcalligra{k}}}{\left({{{{\sf (} \! \rotatebox{146}{\sf c}}}^{\divideontimes }}^{1-{\mathfrak{L}}_{j}^{{\rotatebox{180}{{\rm h}}}^{\diamond }}}-1\right)}^{{\Omega }_{{\mathbbm{w}}-j}}}{{\left({{{\sf (} \! \rotatebox{146}{\sf c}}}^{\divideontimes }-1\right)}^{\sum_{j=1}^{{\mathcalligra{k}}}{\Omega }_{{\mathbbm{w}}-j}-1}}\right)+\iota 1-{{\text{log}}}_{{{{\sf (} \! \rotatebox{146}{\sf c}}}^{\divideontimes }}\left(1+\frac{\prod_{j=1}^{{\mathcalligra{k}}}{\left({{{{\sf (} \! \rotatebox{146}{\sf c}}}^{\divideontimes }}^{1-{ \frown\!\!\!\!\!\! {\smallint} }_{j}^{{\rotatebox{180}{{\rm h}}}^{\diamond }}}-1\right)}^{{\Omega }_{{\mathbbm{w}}-j}}}{{\left({{{\sf (} \! \rotatebox{146}{\sf c}}}^{\divideontimes }-1\right)}^{\sum_{j=1}^{{\mathcalligra{k}}}{\Omega }_{{\mathbbm{w}}-j}-1}}\right),\\ 1-{{\text{log}}}_{{{{\sf (} \! \rotatebox{146}{\sf c}}}^{\divideontimes }}\left(1+\frac{\prod_{j=1}^{{\mathcalligra{k}}}{\left({{{{\sf (} \! \rotatebox{146}{\sf c}}}^{\divideontimes }}^{1-{\mathfrak{L}}_{j}^{{ { \bar{\d{\rm L}}} }^{\diamond }}}-1\right)}^{{\Omega }_{{\mathbbm{w}}-j}}}{{\left({{{\sf (} \! \rotatebox{146}{\sf c}}}^{\divideontimes }-1\right)}^{\sum_{j=1}^{{\mathcalligra{k}}}{\Omega }_{{\mathbbm{w}}-j}-1}}\right)+\iota 1-{{\text{log}}}_{{{{\sf (} \! \rotatebox{146}{\sf c}}}^{\divideontimes }}\left(1+\frac{\prod_{j=1}^{{\mathcalligra{k}}}{\left({{{{\sf (} \! \rotatebox{146}{\sf c}}}^{\divideontimes }}^{1-{ \frown\!\!\!\!\!\! {\smallint} }_{j}^{{ { \bar{\d{\rm L}}} }^{\diamond }}}-1\right)}^{{\Omega }_{{\mathbbm{w}}-j}}}{{\left({{{\sf (} \! \rotatebox{146}{\sf c}}}^{\divideontimes }-1\right)}^{\sum_{j=1}^{{\mathcalligra{k}}}{\Omega }_{{\mathbbm{w}}-j}-1}}\right)\end{array}\right)$$

$$\oplus \left(\begin{array}{c}1-{{\text{log}}}_{{{{\sf (} \! \rotatebox{146}{\sf c}}}^{\divideontimes }}\left(1+\frac{{\left({{{{\sf (} \! \rotatebox{146}{\sf c}}}^{\divideontimes }}^{1-{\mathfrak{L}}_{1}^{{\rotatebox{180}{{\rm h}}}^{\diamond }}}-1\right)}^{{\Omega }_{{\mathbbm{w}}-{\mathcalligra{k}}+1}}}{{\left({{{\sf (} \! \rotatebox{146}{\sf c}}}^{\divideontimes }-1\right)}^{{\Omega }_{{\mathbbm{w}}-1}-{\mathcalligra{k}}+1}}\right)+\iota {1-{\text{log}}}_{{{{\sf (} \! \rotatebox{146}{\sf c}}}^{\divideontimes }}\left(1+\frac{{\left({{{{\sf (} \! \rotatebox{146}{\sf c}}}^{\divideontimes }}^{{1- \frown\!\!\!\!\!\! {\smallint} }_{1}^{{\rotatebox{180}{{\rm h}}}^{\diamond }}}-1\right)}^{{\Omega }_{{\mathbbm{w}}-{\mathcalligra{k}}+1}}}{{\left({{{\sf (} \! \rotatebox{146}{\sf c}}}^{\divideontimes }-1\right)}^{{\Omega }_{{\mathbbm{w}}-1}-{\mathcalligra{k}}+1}}\right),\\ 1-{{\text{log}}}_{{{{\sf (} \! \rotatebox{146}{\sf c}}}^{\divideontimes }}\left(1+\frac{{\left({{{{\sf (} \! \rotatebox{146}{\sf c}}}^{\divideontimes }}^{1-{\mathfrak{L}}_{1}^{{ { \bar{\d{\rm L}}} }^{\diamond }}}-1\right)}^{{\Omega }_{{\mathbbm{w}}-{\mathcalligra{k}}+1}}}{{\left({{{\sf (} \! \rotatebox{146}{\sf c}}}^{\divideontimes }-1\right)}^{{\Omega }_{{\mathbbm{w}}-1}-{\mathcalligra{k}}+1}}\right)+\iota {1-{\text{log}}}_{{{{\sf (} \! \rotatebox{146}{\sf c}}}^{\divideontimes }}\left(1+\frac{{\left({{{{\sf (} \! \rotatebox{146}{\sf c}}}^{\divideontimes }}^{{1- \frown\!\!\!\!\!\! {\smallint} }_{1}^{{ { \bar{\d{\rm L}}} }^{\diamond }}}-1\right)}^{{\Omega }_{{\mathbbm{w}}-{\mathcalligra{k}}+1}}}{{\left({{{\sf (} \! \rotatebox{146}{\sf c}}}^{\divideontimes }-1\right)}^{{\Omega }_{{\mathbbm{w}}-1}-{\mathcalligra{k}}+1}}\right)\end{array}\right)$$

$$=\left(A, B\right)$$

where

$$A=\left(\begin{array}{c}1-{{\text{log}}}_{{{{\sf (} \! \rotatebox{146}{\sf c}}}^{\divideontimes }}\left(1+\frac{\left({{{{\sf (} \! \rotatebox{146}{\sf c}}}^{\divideontimes }}^{{{\text{log}}}_{{{{\sf (} \! \rotatebox{146}{\sf c}}}^{\divideontimes }}\left(1+\frac{\prod_{j=1}^{{\mathcalligra{k}}}{\left({{{{\sf (} \! \rotatebox{146}{\sf c}}}^{\divideontimes }}^{1-{\mathfrak{L}}_{j}^{{\rotatebox{180}{{\rm h}}}^{\diamond }}}-1\right)}^{{\Omega }_{{\mathbbm{w}}-j}}}{{\left({{{\sf (} \! \rotatebox{146}{\sf c}}}^{\divideontimes }-1\right)}^{\sum_{j=1}^{{\mathcalligra{k}}}{\Omega }_{{\mathbbm{w}}-j}-1}}\right)}-1\right)\left({{{{\sf (} \! \rotatebox{146}{\sf c}}}^{\divideontimes }}^{{{\text{log}}}_{{{{\sf (} \! \rotatebox{146}{\sf c}}}^{\divideontimes }}\left(1+\frac{{\left({{{{\sf (} \! \rotatebox{146}{\sf c}}}^{\divideontimes }}^{1-{\mathfrak{L}}_{j}^{{\rotatebox{180}{{\rm h}}}^{\diamond }}}-1\right)}^{{\Omega }_{{\mathbbm{w}}-{\mathcalligra{k}}+1}}}{{\left({{{\sf (} \! \rotatebox{146}{\sf c}}}^{\divideontimes }-1\right)}^{{\Omega }_{{\mathbbm{w}}-{\mathcalligra{k}}+1}-1}}\right)}-1\right)}{{{{\sf (} \! \rotatebox{146}{\sf c}}}^{\divideontimes }-1}\right)\\ +\iota 1-{{\text{log}}}_{{{{\sf (} \! \rotatebox{146}{\sf c}}}^{\divideontimes }}\left(1+\frac{\left({{{{\sf (} \! \rotatebox{146}{\sf c}}}^{\divideontimes }}^{{{\text{log}}}_{{{{\sf (} \! \rotatebox{146}{\sf c}}}^{\divideontimes }}\left(1+\frac{\prod_{j=1}^{{\mathcalligra{k}}}{\left({{{{\sf (} \! \rotatebox{146}{\sf c}}}^{\divideontimes }}^{1-{ \frown\!\!\!\!\!\! {\smallint} }_{j}^{{\rotatebox{180}{{\rm h}}}^{\diamond }}}-1\right)}^{{\Omega }_{{\mathbbm{w}}-j}}}{{\left({{{\sf (} \! \rotatebox{146}{\sf c}}}^{\divideontimes }-1\right)}^{\sum_{j=1}^{{\mathcalligra{k}}}{\Omega }_{{\mathbbm{w}}-j}-1}}\right)}-1\right)\left({{{{\sf (} \! \rotatebox{146}{\sf c}}}^{\divideontimes }}^{{{\text{log}}}_{{{{\sf (} \! \rotatebox{146}{\sf c}}}^{\divideontimes }}\left(1+\frac{{\left({{{{\sf (} \! \rotatebox{146}{\sf c}}}^{\divideontimes }}^{1-{ \frown\!\!\!\!\!\! {\smallint} }_{j}^{{\rotatebox{180}{{\rm h}}}^{\diamond }}}-1\right)}^{{\Omega }_{{\mathbbm{w}}-{\mathcalligra{k}}+1}}}{{\left({{{\sf (} \! \rotatebox{146}{\sf c}}}^{\divideontimes }-1\right)}^{{\Omega }_{{\mathbbm{w}}-{\mathcalligra{k}}+1}-1}}\right)}-1\right)}{{{{\sf (} \! \rotatebox{146}{\sf c}}}^{\divideontimes }-1}\right)\end{array}\right)$$

And

$$B=\left(\begin{array}{c}1-{{\text{log}}}_{{{{\sf (} \! \rotatebox{146}{\sf c}}}^{\divideontimes }}\left(1+\frac{\left({{{{\sf (} \! \rotatebox{146}{\sf c}}}^{\divideontimes }}^{{{\text{log}}}_{{{{\sf (} \! \rotatebox{146}{\sf c}}}^{\divideontimes }}\left(1+\frac{\prod_{j=1}^{{\mathcalligra{k}}}{\left({{{{\sf (} \! \rotatebox{146}{\sf c}}}^{\divideontimes }}^{1-{\mathfrak{L}}_{j}^{{\rotatebox{180}{{\rm h}}}^{\diamond }}}-1\right)}^{{\Omega }_{{\mathbbm{w}}-j}}}{{\left({{{\sf (} \! \rotatebox{146}{\sf c}}}^{\divideontimes }-1\right)}^{\sum_{j=1}^{{\mathcalligra{k}}}{\Omega }_{{\mathbbm{w}}-j}-1}}\right)}-1\right)\left({{{{\sf (} \! \rotatebox{146}{\sf c}}}^{\divideontimes }}^{{{\text{log}}}_{{{{\sf (} \! \rotatebox{146}{\sf c}}}^{\divideontimes }}\left(1+\frac{{\left({{{{\sf (} \! \rotatebox{146}{\sf c}}}^{\divideontimes }}^{1-{\mathfrak{L}}_{j}^{{\rotatebox{180}{{\rm h}}}^{\diamond }}}-1\right)}^{{\Omega }_{{\mathbbm{w}}-{\mathcalligra{k}}+1}}}{{\left({{{\sf (} \! \rotatebox{146}{\sf c}}}^{\divideontimes }-1\right)}^{{\Omega }_{{\mathbbm{w}}-{\mathcalligra{k}}+1}-1}}\right)}-1\right)}{{{{\sf (} \! \rotatebox{146}{\sf c}}}^{\divideontimes }-1}\right)\\ +\iota 1-{{\text{log}}}_{{{{\sf (} \! \rotatebox{146}{\sf c}}}^{\divideontimes }}\left(1+\frac{\left({{{{\sf (} \! \rotatebox{146}{\sf c}}}^{\divideontimes }}^{{{\text{log}}}_{{{{\sf (} \! \rotatebox{146}{\sf c}}}^{\divideontimes }}\left(1+\frac{\prod_{j=1}^{{\mathcalligra{k}}}{\left({{{{\sf (} \! \rotatebox{146}{\sf c}}}^{\divideontimes }}^{1-{ \frown\!\!\!\!\!\! {\smallint} }_{j}^{{\rotatebox{180}{{\rm h}}}^{\diamond }}}-1\right)}^{{\Omega }_{{\mathbbm{w}}-j}}}{{\left({{{\sf (} \! \rotatebox{146}{\sf c}}}^{\divideontimes }-1\right)}^{\sum_{j=1}^{{\mathcalligra{k}}}{\Omega }_{{\mathbbm{w}}-j}-1}}\right)}-1\right)\left({{{{\sf (} \! \rotatebox{146}{\sf c}}}^{\divideontimes }}^{{{\text{log}}}_{{{{\sf (} \! \rotatebox{146}{\sf c}}}^{\divideontimes }}\left(1+\frac{{\left({{{{\sf (} \! \rotatebox{146}{\sf c}}}^{\divideontimes }}^{1-{ \frown\!\!\!\!\!\! {\smallint} }_{j}^{{\rotatebox{180}{{\rm h}}}^{\diamond }}}-1\right)}^{{\Omega }_{{\mathbbm{w}}-{\mathcalligra{k}}+1}}}{{\left({{{\sf (} \! \rotatebox{146}{\sf c}}}^{\divideontimes }-1\right)}^{{\Omega }_{{\mathbbm{w}}-{\mathcalligra{k}}+1}-1}}\right)}-1\right)}{{{{\sf (} \! \rotatebox{146}{\sf c}}}^{\divideontimes }-1}\right)\end{array}\right)$$

$$=\left(\begin{array}{c}1-{{\text{log}}}_{{{{\sf (} \! \rotatebox{146}{\sf c}}}^{\divideontimes }}\left(1+\frac{\prod_{j=1}^{{\mathcalligra{k}}+1}{\left({{{{\sf (} \! \rotatebox{146}{\sf c}}}^{\divideontimes }}^{1-{\mathfrak{L}}_{j}^{{\rotatebox{180}{{\rm h}}}^{\diamond }}}-1\right)}^{{\Omega }_{{\mathbbm{w}}-j}}}{{\left({{{\sf (} \! \rotatebox{146}{\sf c}}}^{\divideontimes }-1\right)}^{\sum_{j=1}^{{\mathcalligra{k}}+1}{\Omega }_{{\mathbbm{w}}-j}-1}}\right)+\iota 1-{{\text{log}}}_{{{{\sf (} \! \rotatebox{146}{\sf c}}}^{\divideontimes }}\left(1+\frac{\prod_{j=1}^{{\mathcalligra{k}}+1}{\left({{{{\sf (} \! \rotatebox{146}{\sf c}}}^{\divideontimes }}^{1-{ \frown\!\!\!\!\!\! {\smallint} }_{j}^{{\rotatebox{180}{{\rm h}}}^{\diamond }}}-1\right)}^{{\Omega }_{{\mathbbm{w}}-j}}}{{\left({{{\sf (} \! \rotatebox{146}{\sf c}}}^{\divideontimes }-1\right)}^{\sum_{j=1}^{{\mathcalligra{k}}+1}{\Omega }_{{\mathbbm{w}}-j}-1}}\right),\\ 1-{{\text{log}}}_{{{{\sf (} \! \rotatebox{146}{\sf c}}}^{\divideontimes }}\left(1+\frac{\prod_{j=1}^{{\mathcalligra{k}}+1}{\left({{{{\sf (} \! \rotatebox{146}{\sf c}}}^{\divideontimes }}^{1-{\mathfrak{L}}_{j}^{{ { \bar{\d{\rm L}}} }^{\diamond }}}-1\right)}^{{\Omega }_{{\mathbbm{w}}-j}}}{{\left({{{\sf (} \! \rotatebox{146}{\sf c}}}^{\divideontimes }-1\right)}^{\sum_{j=1}^{{\mathcalligra{k}}+1}{\Omega }_{{\mathbbm{w}}-j}-1}}\right)+\iota 1-{{\text{log}}}_{{{{\sf (} \! \rotatebox{146}{\sf c}}}^{\divideontimes }}\left(1+\frac{\prod_{j=1}^{{\mathcalligra{k}}+1}{\left({{{{\sf (} \! \rotatebox{146}{\sf c}}}^{\divideontimes }}^{1-{ \frown\!\!\!\!\!\! {\smallint} }_{j}^{{ { \bar{\d{\rm L}}} }^{\diamond }}}-1\right)}^{{\Omega }_{{\mathbbm{w}}-j}}}{{\left({{{\sf (} \! \rotatebox{146}{\sf c}}}^{\divideontimes }-1\right)}^{\sum_{j=1}^{{\mathcalligra{k}}+1}{\Omega }_{{\mathbbm{w}}-j}-1}}\right)\end{array}\right)$$

Hence, Eq. (2) is valid for $n={\mathcalligra{k}}+1$. Therefore, Eq. (2) is valid for all $n$.

Now we will discuss that CFRFWA AOs satisfy the following characteristics. Here ${\hat{C} }_{om.-j}=\left({\mathfrak{L}}_{j}^{{\rotatebox{180}{{\rm h}}}^{\diamond }}+\iota { \frown\!\!\!\!\!\! {\smallint} }_{j}^{{\rotatebox{180}{{\rm h}}}^{\diamond }}, {\mathfrak{L}}_{j}^{{ { \bar{\d{\rm L}}} }^{\diamond }}+\iota { \frown\!\!\!\!\!\! {\smallint} }_{j}^{{ { \bar{\d{\rm L}}} }^{\diamond }}\right)$ and ${\hat{C} }_{om.-j}^{\divideontimes }=\left({{\mathfrak{L}}^{\divideontimes }}_{j}^{{\rotatebox{180}{{\rm h}}}^{\diamond }}+\iota { \frown\!\!\!\!\!\! {\smallint} \divideontimes }_{j}^{{\rotatebox{180}{{\rm h}}}^{\diamond }}, {{\mathfrak{L}}^{\divideontimes }}_{j}^{{ { \bar{\d{\rm L}}} }^{\diamond }}+\iota { \frown\!\!\!\!\!\! {\smallint} \divideontimes }_{j}^{{ { \bar{\d{\rm L}}} }^{\diamond }}\right) \left(j=1, 2, .., n\right)$ represent two collections of CFRNs, then

1. (Idempotency): If all ${\hat{C} }_{om.-j}={\hat{C} }_{om.} \forall j$ then

$$CFRFWA\left({\hat{C} }_{om.-1}, {\hat{C} }_{om.-2}, \dots , {\hat{C} }_{om.-n}\right)={\hat{C} }_{om.}$$

2. Boundedness: Let ${{\hat{C} }_{om.}}^{-}=\left(\underset{j}{{\text{min}}}\left\{{\mathfrak{L}}_{j}^{{\rotatebox{180}{{\rm h}}}^{\diamond }}\right\}+\iota \underset{j}{{\text{min}}}\left\{{ \frown\!\!\!\!\!\! {\smallint} }_{j}^{{\rotatebox{180}{{\rm h}}}^{\diamond }}\right\}, \underset{j}{{\text{min}}}\left\{{\mathfrak{L}}_{j}^{{ { \bar{\d{\rm L}}} }^{\diamond }}\right\}+\iota \underset{j}{{\text{min}}}\left\{{ \frown\!\!\!\!\!\! {\smallint} }_{j}^{{ { \bar{\d{\rm L}}} }^{\diamond }}\right\}\right),$ and ${{\hat{C} }_{om.}}^{+}=\left(\underset{j}{{\text{max}}}\left\{{\mathfrak{L}}_{j}^{{\rotatebox{180}{{\rm h}}}^{\diamond }}\right\}+\iota \underset{j}{{\text{max}}}\left\{{ \frown\!\!\!\!\!\! {\smallint} }_{j}^{{\rotatebox{180}{{\rm h}}}^{\diamond }}\right\}, \underset{j}{{\text{max}}}\left\{{\mathfrak{L}}_{j}^{{ { \bar{\d{\rm L}}} }^{\diamond }}\right\}+\iota \underset{j}{{\text{max}}}\left\{{ \frown\!\!\!\!\!\! {\smallint} }_{j}^{{ { \bar{\d{\rm L}}} }^{\diamond }}\right\}\right)$. Then

$${{\hat{C} }_{om.}}^{-}\le CFRFWA\left({\hat{C} }_{om.-1}, {\hat{C} }_{om.-2}, \dots , {\hat{C} }_{om.-n}\right)\le {{\hat{C} }_{om.}}^{+}$$

3. Monotonicity: If ${\mathfrak{L}}_{j}^{{\rotatebox{180}{{\rm h}}}^{\diamond }}\le {{\mathfrak{L}}^{\divideontimes }}_{j}^{{\rotatebox{180}{{\rm h}}}^{\diamond }}$, ${ \frown\!\!\!\!\!\! {\smallint} }_{j}^{{\rotatebox{180}{{\rm h}}}^{\diamond }}\le { \frown\!\!\!\!\!\! {\smallint} \divideontimes }_{j}^{{\rotatebox{180}{{\rm h}}}^{\diamond }}$, ${\mathfrak{L}}_{j}^{{ { \bar{\d{\rm L}}} }^{\diamond }}\le {{\mathfrak{L}}^{\divideontimes }}_{j}^{{ { \bar{\d{\rm L}}} }^{\diamond }}$, ${ \frown\!\!\!\!\!\! {\smallint} }_{j}^{{ { \bar{\d{\rm L}}} }^{\diamond }}\le { \frown\!\!\!\!\!\! {\smallint} \divideontimes }_{j}^{{ { \bar{\d{\rm L}}} }^{\diamond }}\forall j$, then

$$CFRFWA\left({\hat{C} }_{om.-1}, {\hat{C} }_{om.-2}, \dots , {\hat{C} }_{om.-n}\right)\le CFRFWA\left({\hat{C} }_{om.-1}^{\divideontimes }, {\hat{C} }_{om.-2}^{\divideontimes }, \dots , {\hat{C} }_{om.-n}^{\divideontimes }\right)$$

### Complex fuzzy rough Frank ordered weighted average (CFRFOWA) AOs

The notion of CFROWA AO is defined here. Moreover, we will analyze the characteristics of this delivered approach.

#### Definition 11

Let ${\hat{C} }_{om.-j}=\left({\mathfrak{L}}_{j}^{{\rotatebox{180}{{\rm h}}}^{\diamond }}+\iota { \frown\!\!\!\!\!\! {\smallint} }_{j}^{{\rotatebox{180}{{\rm h}}}^{\diamond }}, {\mathfrak{L}}_{j}^{{ { \bar{\d{\rm L}}} }^{\diamond }}+\iota { \frown\!\!\!\!\!\! {\smallint} }_{j}^{{ { \bar{\d{\rm L}}} }^{\diamond }}\right) \left(j=1, 2, .., n\right)$ be a collection of CFRNs, then

3 $$CFRFOWA \left({\hat{\text{C}}}_{om.-1}, {\hat{\text{C}} }_{om.-2}, \ldots , {\hat{\text{C}} }_{om.-n}\right)=\begin{array}{c}n\\ \oplus \\ j=1\end{array}{\Omega }_{{\mathbbm{w}}-j}{\hat{\text{C}} }_{om.-\mathfrak{d}\left(j\right)}$$

Where, $\left(\mathfrak{d}\left(1\right),\mathfrak{d}\left(2\right),\dots ,\mathfrak{d}\left(n\right)\right)$ represent the permutation of $j=1, 2, .., n$ with ${\hat{C} }_{om.-\mathfrak{d}\left(j-1\right)}\ge {\hat{C} }_{om.-\mathfrak{d}\left(j\right)}$ for all $j.$

#### Theorem 3

*Let* ${\hat{C} }_{om.-j}=\left({\mathfrak{L}}_{j}^{{\rotatebox{180}{{\rm h}}}^{\diamond }}+\iota { \frown\!\!\!\!\!\! {\smallint} }_{j}^{{\rotatebox{180}{{\rm h}}}^{\diamond }}, {\mathfrak{L}}_{j}^{{ { \bar{\d{\rm L}}} }^{\diamond }}+\iota { \frown\!\!\!\!\!\! {\smallint} }_{j}^{{ { \bar{\d{\rm L}}} }^{\diamond }}\right) \left(j=1, 2, .., n\right)$ *be a collection of CFRNs, then the aggregated result by using* Eq. (3) is again CFRN

4 $$\begin{aligned} & CFRFOWA\left({\hat{C} }_{om.-1}, {\hat{C} }_{om.-2}, \dots , {\hat{C} }_{om.-n}\right)\\ & \quad =\left(\begin{array}{c}1-\left({{\text{log}}}_{{{{\sf (} \! \rotatebox{146}{\sf c}}}^{\divideontimes }}\left(1+\prod_{j=1}^{n}{\left({{{{\sf (} \! \rotatebox{146}{\sf c}}}^{\divideontimes }}^{1-{\mathfrak{L}}_{\mathfrak{d}\left(j\right)}^{{\rotatebox{180}{{\rm h}}}^{\diamond }}}-1\right)}^{{\Omega }_{{\mathbbm{w}}-j}}\right)\right)+\iota 1-\left({{\text{log}}}_{{{{\sf (} \! \rotatebox{146}{\sf c}}}^{\divideontimes }}\left(1+\prod_{j=1}^{n}{\left({{{{\sf (} \! \rotatebox{146}{\sf c}}}^{\divideontimes }}^{1-{ \frown\!\!\!\!\!\! {\smallint} }_{\mathfrak{d}\left(j\right)}^{{\rotatebox{180}{{\rm h}}}^{\diamond }}}-1\right)}^{{\Omega }_{{\mathbbm{w}}-j}}\right)\right),\\ 1-\left({{\text{log}}}_{{{{\sf (} \! \rotatebox{146}{\sf c}}}^{\divideontimes }}\left(1+\prod_{j=1}^{n}{\left({{{{\sf (} \! \rotatebox{146}{\sf c}}}^{\divideontimes }}^{1-{\mathfrak{L}}_{\mathfrak{d}\left(j\right)}^{{ { \bar{\d{\rm L}}} }^{\diamond }}}-1\right)}^{{\Omega }_{{\mathbbm{w}}-j}}\right)\right)+\iota 1-\left({{\text{log}}}_{{{{\sf (} \! \rotatebox{146}{\sf c}}}^{\divideontimes }}\left(1+\prod_{j=1}^{n}{\left({{{{\sf (} \! \rotatebox{146}{\sf c}}}^{\divideontimes }}^{1-{ \frown\!\!\!\!\!\! {\smallint} }_{\mathfrak{d}\left(j\right)}^{{ { \bar{\d{\rm L}}} }^{\diamond }}}-1\right)}^{{\Omega }_{{\mathbbm{w}}-j}}\right)\right),\end{array}\right)\end{aligned}$$

#### Proof

Proof is the same as Theorem 2.

Let ${\hat{C} }_{om.-j}=\left({\mathfrak{L}}_{j}^{{\rotatebox{180}{{\rm h}}}^{\diamond }}+\iota { \frown\!\!\!\!\!\! {\smallint} }_{j}^{{\rotatebox{180}{{\rm h}}}^{\diamond }}, {\mathfrak{L}}_{j}^{{ { \bar{\d{\rm L}}} }^{\diamond }}+\iota { \frown\!\!\!\!\!\! {\smallint} }_{j}^{{ { \bar{\d{\rm L}}} }^{\diamond }}\right)$ and ${\hat{C} }_{om.-j}^{\divideontimes }=\left({{\mathfrak{L}}^{\divideontimes }}_{j}^{{\rotatebox{180}{{\rm h}}}^{\diamond }}+\iota { \frown\!\!\!\!\!\! {\smallint} \divideontimes }_{j}^{{\rotatebox{180}{{\rm h}}}^{\diamond }}, {{\mathfrak{L}}^{\divideontimes }}_{j}^{{ { \bar{\d{\rm L}}} }^{\diamond }}+\iota { \frown\!\!\!\!\!\! {\smallint} \divideontimes }_{j}^{{ { \bar{\d{\rm L}}} }^{\diamond }}\right) \left(j=1, 2, .., n\right)$ be two collections of CFRNs, then CFRFOWA AOs follow the characteristics given by

1. (Idempotency): If all ${\hat{C} }_{om.-j}={\hat{C} }_{om.} \forall j$ then

$$CFRFOWA\left({\hat{C} }_{om.-1}, {\hat{C} }_{om.-2}, \dots , {\hat{C} }_{om.-n}\right)={\hat{C} }_{om.}$$

2. Boundedness: Let ${{\hat{C} }_{om.}}^{-}=\left(\underset{j}{{\text{min}}}\left\{{\mathfrak{L}}_{j}^{{\rotatebox{180}{{\rm h}}}^{\diamond }}\right\}+\iota \underset{j}{{\text{min}}}\left\{{ \frown\!\!\!\!\!\! {\smallint} }_{j}^{{\rotatebox{180}{{\rm h}}}^{\diamond }}\right\}, \underset{j}{{\text{min}}}\left\{{\mathfrak{L}}_{j}^{{ { \bar{\d{\rm L}}} }^{\diamond }}\right\}+\iota \underset{j}{{\text{min}}}\left\{{ \frown\!\!\!\!\!\! {\smallint} }_{j}^{{ { \bar{\d{\rm L}}} }^{\diamond }}\right\}\right),$ and ${{\hat{C} }_{om.}}^{+}=\left(\underset{j}{{\text{max}}}\left\{{\mathfrak{L}}_{j}^{{\rotatebox{180}{{\rm h}}}^{\diamond }}\right\}+\iota \underset{j}{{\text{max}}}\left\{{ \frown\!\!\!\!\!\! {\smallint} }_{j}^{{\rotatebox{180}{{\rm h}}}^{\diamond }}\right\}, \underset{j}{{\text{max}}}\left\{{\mathfrak{L}}_{j}^{{ { \bar{\d{\rm L}}} }^{\diamond }}\right\}+\iota \underset{j}{{\text{max}}}\left\{{ \frown\!\!\!\!\!\! {\smallint} }_{j}^{{ { \bar{\d{\rm L}}} }^{\diamond }}\right\}\right)$. Then

$${{\hat{C} }_{om.}}^{-}\le CFRFOWA\left({\hat{C} }_{om.-1}, {\hat{C} }_{om.-2}, \dots , {\hat{C} }_{om.-n}\right)\le {{\hat{C} }_{om.}}^{+}$$

3. Monotonicity: If ${\mathfrak{L}}_{j}^{{\rotatebox{180}{{\rm h}}}^{\diamond }}\le {{\mathfrak{L}}^{\divideontimes }}_{j}^{{\rotatebox{180}{{\rm h}}}^{\diamond }}$, ${ \frown\!\!\!\!\!\! {\smallint} }_{j}^{{\rotatebox{180}{{\rm h}}}^{\diamond }}\le { \frown\!\!\!\!\!\! {\smallint} \divideontimes }_{j}^{{\rotatebox{180}{{\rm h}}}^{\diamond }}$, ${\mathfrak{L}}_{j}^{{ { \bar{\d{\rm L}}} }^{\diamond }}\le {{\mathfrak{L}}^{\divideontimes }}_{j}^{{ { \bar{\d{\rm L}}} }^{\diamond }}$, ${ \frown\!\!\!\!\!\! {\smallint} }_{j}^{{ { \bar{\d{\rm L}}} }^{\diamond }}\le { \frown\!\!\!\!\!\! {\smallint} \divideontimes }_{j}^{{ { \bar{\d{\rm L}}} }^{\diamond }}\forall j$, then

$$CFRFOWA\left({\hat{C} }_{om.-1}, {\hat{C} }_{om.-2}, \dots , {\hat{C} }_{om.-n}\right)\le CFRFOWA\left({\hat{C} }_{om.-1}^{\divideontimes }, {\hat{C} }_{om.-2}^{\divideontimes }, \dots , {\hat{C} }_{om.-n}^{\divideontimes }\right)$$

### Complex fuzzy rough Frank hybrid weighted average (CFRHWA) AOs

Here in this section, by combining the characteristics of the CFRFWA and CFRFOWA, we define the notion of CFRFHWA AOs as: 

#### Definition 12

 Let ${\hat{C} }_{om.-j}=\left({\mathfrak{L}}_{j}^{{\rotatebox{180}{{\rm h}}}^{\diamond }}+\iota { \frown\!\!\!\!\!\! {\smallint} }_{j}^{{\rotatebox{180}{{\rm h}}}^{\diamond }}, {\mathfrak{L}}_{j}^{{ { \bar{\d{\rm L}}} }^{\diamond }}+\iota { \frown\!\!\!\!\!\! {\smallint} }_{j}^{{ { \bar{\d{\rm L}}} }^{\diamond }}\right) \left(j=1, 2, .., n\right)$ be a collection of CFRNs, then CFRHWA AOs are given by

$$CFRFHWA\left({\hat{C} }_{om.-1}, {\hat{C} }_{om.-2}, \dots , {\hat{C} }_{om.-n}\right)=\begin{array}{c}n\\ \oplus \\ j=1\end{array}{{\Omega }^{\between }}_{{\mathbbm{w}}-j}{{\hat{C} }^{\between }}_{om.-\mathfrak{d}\left(j\right)}$$

$$=\left(\begin{array}{c}1-{{\text{log}}}_{{{{\sf (} \! \rotatebox{146}{\sf c}}}^{\divideontimes }}\left(1+\prod_{j=1}^{n}{\left({{{{\sf (} \! \rotatebox{146}{\sf c}}}^{\divideontimes }}^{1-{\mathfrak{L}}_{\mathfrak{d}\left(j\right)}^{{\rotatebox{180}{{\rm h}}}^{\diamond }}}-1\right)}^{{\Omega }_{{\mathbbm{w}}-j}}\right)+\iota 1-{{\text{log}}}_{{{{\sf (} \! \rotatebox{146}{\sf c}}}^{\divideontimes }}\left(1+\prod_{j=1}^{n}{\left({{{{\sf (} \! \rotatebox{146}{\sf c}}}^{\divideontimes }}^{1-{ \frown\!\!\!\!\!\! {\smallint} }_{\mathfrak{d}\left(j\right)}^{{\rotatebox{180}{{\rm h}}}^{\diamond }}}-1\right)}^{{\Omega }_{{\mathbbm{w}}-j}}\right),\\ 1-{{\text{log}}}_{{{{\sf (} \! \rotatebox{146}{\sf c}}}^{\divideontimes }}\left(1+\prod_{j=1}^{n}{\left({{{{\sf (} \! \rotatebox{146}{\sf c}}}^{\divideontimes }}^{1-{\mathfrak{L}}_{\mathfrak{d}\left(j\right)}^{{ { \bar{\d{\rm L}}} }^{\diamond }}}-1\right)}^{{\Omega }_{{\mathbbm{w}}-j}}\right)+\iota 1-{{\text{log}}}_{{{{\sf (} \! \rotatebox{146}{\sf c}}}^{\divideontimes }}\left(1+\prod_{j=1}^{n}{\left({{{{\sf (} \! \rotatebox{146}{\sf c}}}^{\divideontimes }}^{1-{ \frown\!\!\!\!\!\! {\smallint} }_{\mathfrak{d}\left(j\right)}^{{ { \bar{\d{\rm L}}} }^{\diamond }}}-1\right)}^{{\Omega }_{{\mathbbm{w}}-j}}\right)\end{array}\right)$$

where ${\Omega }_{\mathbbm{w}}^{*}=\left({\Omega }_{{\mathbbm{w}}-1}^{*}, {\Omega }_{{\mathbbm{w}}-2}^{*}\dots , {\Omega }_{{\mathbbm{w}}-n}^{*}\right)$ is the linked WV such that $0\le {\Omega }_{{\mathbbm{w}}-j}^{*}\le 1$, and $\sum_{j=1}^{n}{\Omega }_{{\mathbbm{w}}-j}^{*}=1, {\Omega }_{\mathbbm{w}}=\left({\Omega }_{{\mathbbm{w}}-1}, {\Omega }_{{\mathbbm{w}}-2}\dots , {\Omega }_{{\mathbbm{w}}-n}\right)$ is the WV of ${\hat{C} }_{om.-j}\left(j=1, 2, \dots , n\right)$ such that $0\le {\Omega }_{{\mathbbm{w}}-j}\le 1$, and $\sum_{j=1}^{n}{\Omega }_{{\mathbbm{w}}-j}=1$ is the $jth$ major weighted CFR values of ${\hat{C} }_{om.-j}^{*}\left({\hat{C} }_{om.-j}^{*}=n{\Omega }_{{\mathbbm{w}}-j}{\hat{C} }_{om.-j}\right), n$ is the balancing coefficient.

Let ${\hat{C} }_{om.-j}=\left({\mathfrak{L}}_{j}^{{\rotatebox{180}{{\rm h}}}^{\diamond }}+\iota { \frown\!\!\!\!\!\! {\smallint} }_{j}^{{\rotatebox{180}{{\rm h}}}^{\diamond }}, {\mathfrak{L}}_{j}^{{ { \bar{\d{\rm L}}} }^{\diamond }}+\iota { \frown\!\!\!\!\!\! {\smallint} }_{j}^{{ { \bar{\d{\rm L}}} }^{\diamond }}\right)$ and ${\hat{C} }_{om.-j}^{\divideontimes }=\left({{\mathfrak{L}}^{\divideontimes }}_{j}^{{\rotatebox{180}{{\rm h}}}^{\diamond }}+\iota { \frown\!\!\!\!\!\! {\smallint} \divideontimes }_{j}^{{\rotatebox{180}{{\rm h}}}^{\diamond }}, {{\mathfrak{L}}^{\divideontimes }}_{j}^{{ { \bar{\d{\rm L}}} }^{\diamond }}+\iota { \frown\!\!\!\!\!\! {\smallint} \divideontimes }_{j}^{{ { \bar{\d{\rm L}}} }^{\diamond }}\right) \left(j=1, 2, .., n\right)$ be two collections of CFRNs, then the following properties hold for the structure of CFRFHWA AOs.

1. (Idempotency): If all ${\hat{C} }_{om.-j}={\hat{C} }_{om.} \forall j$ then

$$CFRFHWA\left({\hat{C} }_{om.-1}, {\hat{C} }_{om.-2}, \dots , {\hat{C} }_{om.-n}\right)={\hat{C} }_{om.}$$

2. Boundedness: Let ${{\hat{C} }_{om.}}^{-}=\left(\underset{j}{{\text{min}}}\left\{{\mathfrak{L}}_{j}^{{\rotatebox{180}{{\rm h}}}^{\diamond }}\right\}+\iota \underset{j}{{\text{min}}}\left\{{ \frown\!\!\!\!\!\! {\smallint} }_{j}^{{\rotatebox{180}{{\rm h}}}^{\diamond }}\right\}, \underset{j}{{\text{min}}}\left\{{\mathfrak{L}}_{j}^{{ { \bar{\d{\rm L}}} }^{\diamond }}\right\}+\iota \underset{j}{{\text{min}}}\left\{{ \frown\!\!\!\!\!\! {\smallint} }_{j}^{{ { \bar{\d{\rm L}}} }^{\diamond }}\right\}\right),$ and ${{\hat{C} }_{om.}}^{+}=\left(\underset{j}{{\text{max}}}\left\{{\mathfrak{L}}_{j}^{{\rotatebox{180}{{\rm h}}}^{\diamond }}\right\}+\iota \underset{j}{{\text{max}}}\left\{{ \frown\!\!\!\!\!\! {\smallint} }_{j}^{{\rotatebox{180}{{\rm h}}}^{\diamond }}\right\}, \underset{j}{{\text{max}}}\left\{{\mathfrak{L}}_{j}^{{ { \bar{\d{\rm L}}} }^{\diamond }}\right\}+\iota \underset{j}{{\text{max}}}\left\{{ \frown\!\!\!\!\!\! {\smallint} }_{j}^{{ { \bar{\d{\rm L}}} }^{\diamond }}\right\}\right)$. Then

$${{\hat{C} }_{om.}}^{-}\le CFRFHWA\left({\hat{C} }_{om.-1}, {\hat{C} }_{om.-2}, \dots , {\hat{C} }_{om.-n}\right)\le {{\hat{C} }_{om.}}^{+}$$

3. Monotonicity: If ${\mathfrak{L}}_{j}^{{\rotatebox{180}{{\rm h}}}^{\diamond }}\le {{\mathfrak{L}}^{\divideontimes }}_{j}^{{\rotatebox{180}{{\rm h}}}^{\diamond }}$, ${ \frown\!\!\!\!\!\! {\smallint} }_{j}^{{\rotatebox{180}{{\rm h}}}^{\diamond }}\le { \frown\!\!\!\!\!\! {\smallint} \divideontimes }_{j}^{{\rotatebox{180}{{\rm h}}}^{\diamond }}$, ${\mathfrak{L}}_{j}^{{ { \bar{\d{\rm L}}} }^{\diamond }}\le {{\mathfrak{L}}^{\divideontimes }}_{j}^{{ { \bar{\d{\rm L}}} }^{\diamond }}$, ${ \frown\!\!\!\!\!\! {\smallint} }_{j}^{{ { \bar{\d{\rm L}}} }^{\diamond }}\le { \frown\!\!\!\!\!\! {\smallint} \divideontimes }_{j}^{{ { \bar{\d{\rm L}}} }^{\diamond }}\forall j$, then

$$CFRFHWA\left({\hat{C} }_{om.-1}, {\hat{C} }_{om.-2}, \dots , {\hat{C} }_{om.-n}\right)\le CFRFHWA\left({\hat{C} }_{om.-1}^{\divideontimes }, {\hat{C} }_{om.-2}^{\divideontimes }, \dots , {\hat{C} }_{om.-n}^{\divideontimes }\right).$$

#### Remark 1

1. By using ${\Omega }_{\mathbbm{w}}={\left(\frac{1}{n}, \frac{1}{n}, \dots , \frac{1}{n}\right)}^{t}$ we have ${\hat{C} }_{om.-j}^{\divideontimes }=n\times \frac{1}{n}\times {\hat{C} }_{om.-j}={\hat{C} }_{om.-j}$ for $j=1, 2, .., n$. Hence, CFRFHWA AOs degenerate into CFRFOWA AOs.
2. By utilizing ${\Omega }_{{\mathbbm{w}}-j}^{*}={\left(\frac{1}{n}, \frac{1}{n}, \dots , \frac{1}{n}\right)}^{t}$, the CFRFWHA AOs degenerate into CFRFWA AOs.

## Complex fuzzy rough Frank geometric (CFRFG) AOs

The notions of CFRFWG, CFRFOWG, and CFRFHWG AOs are discussed here. Furthermore, the properties of these notions have been discussed.

### Complex fuzzy rough Frank weighted geometric (CFRFWG) AOs

Here, we have elaborated the structure of CFRFWG AOs. Also, we have discussed the properties of these ideas.

#### Definition 13

For the family of CFRNs, the CFRFWG operator is the function ${\left({\hat{C} }_{om.}\right)}^{n}\to \left({\hat{C} }_{om.}\right)$ given by

5 $$CFRFWG\left({\hat{C} }_{om.-1}, {\hat{C} }_{om.-2}, \dots , {\hat{C} }_{om.-n}\right)=\begin{array}{c}n\\ \otimes \\ j=1\end{array}{\left({\hat{C} }_{om.-j}\right)}^{{\Omega }_{{\mathbbm{w}}-j}}$$

#### Theorem 4

*Let* ${\hat{C} }_{om.-j}=\left({\mathfrak{L}}_{j}^{{\rotatebox{180}{{\rm h}}}^{\diamond }}+\iota { \frown\!\!\!\!\!\! {\smallint} }_{j}^{{\rotatebox{180}{{\rm h}}}^{\diamond }}, {\mathfrak{L}}_{j}^{{ { \bar{\d{\rm L}}} }^{\diamond }}+\iota { \frown\!\!\!\!\!\! {\smallint} }_{j}^{{ { \bar{\d{\rm L}}} }^{\diamond }}\right) \left(j=1, 2, .., n\right)$ *be a collection of CFRNs, the aggregating result by using Eq*. (5) *is again CFRN given as*

6 $$\begin{aligned} & CFRFWG\left({\hat{C} }_{om.-1}, {\hat{C} }_{om.-2}, \dots , {\hat{C} }_{om.-n}\right)\\ & \quad =\left(\begin{array}{c}\left({{\text{log}}}_{{{{\sf (} \! \rotatebox{146}{\sf c}}}^{\divideontimes }}\left(1+\prod_{j=1}^{n}{\left({{{{\sf (} \! \rotatebox{146}{\sf c}}}^{\divideontimes }}^{{\mathfrak{L}}_{j}^{{\rotatebox{180}{{\rm h}}}^{\diamond }}}-1\right)}^{{\Omega }_{{\mathbbm{w}}-j}}\right)\right)+\iota \left({{\text{log}}}_{{{{\sf (} \! \rotatebox{146}{\sf c}}}^{\divideontimes }}\left(1+\prod_{j=1}^{n}{\left({{{{\sf (} \! \rotatebox{146}{\sf c}}}^{\divideontimes }}^{{ \frown\!\!\!\!\!\! {\smallint} }_{j}^{{\rotatebox{180}{{\rm h}}}^{\diamond }}}-1\right)}^{{\Omega }_{{\mathbbm{w}}-j}}\right)\right),\\ \left({{\text{log}}}_{{{{\sf (} \! \rotatebox{146}{\sf c}}}^{\divideontimes }}\left(1+\prod_{j=1}^{n}{\left({{{{\sf (} \! \rotatebox{146}{\sf c}}}^{\divideontimes }}^{{\mathfrak{L}}_{j}^{{ { \bar{\d{\rm L}}} }^{\diamond }}}-1\right)}^{{\Omega }_{{\mathbbm{w}}-j}}\right)\right)+\iota \left({{\text{log}}}_{{{{\sf (} \! \rotatebox{146}{\sf c}}}^{\divideontimes }}\left(1+\prod_{j=1}^{n}{\left({{{{\sf (} \! \rotatebox{146}{\sf c}}}^{\divideontimes }}^{{ \frown\!\!\!\!\!\! {\smallint} }_{j}^{{ { \bar{\d{\rm L}}} }^{\diamond }}}-1\right)}^{{\Omega }_{{\mathbbm{w}}-j}}\right)\right),\end{array}\right)\end{aligned}$$

#### Proof

Proof is the same as Theorem 2.

Let ${\hat{C} }_{om.-j}=\left({\mathfrak{L}}_{j}^{{\rotatebox{180}{{\rm h}}}^{\diamond }}+\iota { \frown\!\!\!\!\!\! {\smallint} }_{j}^{{\rotatebox{180}{{\rm h}}}^{\diamond }}, {\mathfrak{L}}_{j}^{{ { \bar{\d{\rm L}}} }^{\diamond }}+\iota { \frown\!\!\!\!\!\! {\smallint} }_{j}^{{ { \bar{\d{\rm L}}} }^{\diamond }}\right)$ and ${\hat{C} }_{om.-j}^{\divideontimes }=\left({{\mathfrak{L}}^{\divideontimes }}_{j}^{{\rotatebox{180}{{\rm h}}}^{\diamond }}+\iota { \frown\!\!\!\!\!\! {\smallint} \divideontimes }_{j}^{{\rotatebox{180}{{\rm h}}}^{\diamond }}, {{\mathfrak{L}}^{\divideontimes }}_{j}^{{ { \bar{\d{\rm L}}} }^{\diamond }}+\iota { \frown\!\!\!\!\!\! {\smallint} \divideontimes }_{j}^{{ { \bar{\d{\rm L}}} }^{\diamond }}\right) \left(j=1, 2, .., n\right)$ be two collections of CFRNs, then the following properties hold for CFRFWG AOs.

1. (Idempotency): If all ${\hat{C} }_{om.-j}={\hat{C} }_{om.} \forall j$ then

$$CFRFWG\left({\hat{C} }_{om.-1}, {\hat{C} }_{om.-2}, \dots , {\hat{C} }_{om.-n}\right)={\hat{C} }_{om.}$$

2. Boundedness: Let ${{\hat{\text{C}} }_{om.}}^{-}=\left(\underset{j}{{\text{min}}}\left\{{\mathfrak{L}}_{j}^{{{{\rotatebox{180}{{\rm h}}}}}{\diamond }}\right\}+\iota \underset{j}{{\text{min}}}\left\{{ \frown\!\!\!\!\!\! {\smallint} }_{j}^{{{{\rotatebox{180}{{\rm h}}}}}{\diamond }}\right\}, \underset{j}{{\text{min}}}\left\{{\mathfrak{L}}_{j}^{{{{ { \bar{\d{\rm L}}} }}}{\diamond }}\right\}+\iota \underset{j}{{\text{min}}}\left\{{ \frown\!\!\!\!\!\! {\smallint} }_{j}^{{{{ { \bar{\d{\rm L}}} }}}{\diamond }}\right\}\right),$ and ${{\hat{\text{C}} }_{om.}}^{+}=\left(\underset{j}{{\text{max}}}\left\{{\mathfrak{L}}_{j}^{{{{\rotatebox{180}{{\rm h}}}}}{\diamond }}\right\}+\iota \underset{j}{{\text{max}}}\left\{{ \frown\!\!\!\!\!\! {\smallint} }_{j}^{{{{\rotatebox{180}{{\rm h}}}}}{\diamond }}\right\}, \underset{j}{{\text{max}}}\left\{{\mathfrak{L}}_{j}^{{{{ { \bar{\d{\rm L}}} }}}{\diamond }}\right\}+\iota \underset{j}{{\text{max}}}\left\{{ \frown\!\!\!\!\!\! {\smallint} }_{j}^{{{{ { \bar{\d{\rm L}}} }}}{\diamond }}\right\}\right)$. Then

$${{\hat{C} }_{om.}}^{-}\le CFRFWG\left({\hat{C} }_{om.-1}, {\hat{C} }_{om.-2}, \dots , {\hat{C} }_{om.-n}\right)\le {{\hat{C} }_{om.}}^{+}$$

3. Monotonicity: If ${\mathfrak{L}}_{j}^{{\rotatebox{180}{{\rm h}}}^{\diamond }}\le {{\mathfrak{L}}^{\divideontimes }}_{j}^{{\rotatebox{180}{{\rm h}}}^{\diamond }}$, ${ \frown\!\!\!\!\!\! {\smallint} }_{j}^{{\rotatebox{180}{{\rm h}}}^{\diamond }}\le { \frown\!\!\!\!\!\! {\smallint} \divideontimes }_{j}^{{\rotatebox{180}{{\rm h}}}^{\diamond }}$, ${\mathfrak{L}}_{j}^{{ { \bar{\d{\rm L}}} }^{\diamond }}\le {{\mathfrak{L}}^{\divideontimes }}_{j}^{{ { \bar{\d{\rm L}}} }^{\diamond }}$, ${ \frown\!\!\!\!\!\! {\smallint} }_{j}^{{ { \bar{\d{\rm L}}} }^{\diamond }}\le { \frown\!\!\!\!\!\! {\smallint} \divideontimes }_{j}^{{ { \bar{\d{\rm L}}} }^{\diamond }}\forall j$, then

$$CFRFWG\left({\hat{C} }_{om.-1}, {\hat{C} }_{om.-2}, \dots , {\hat{C} }_{om.-n}\right)\le CFRFWG\left({\hat{C} }_{om.-1}^{\divideontimes }, {\hat{C} }_{om.-2}^{\divideontimes }, \dots , {\hat{C} }_{om.-n}^{\divideontimes }\right)$$

### Complex fuzzy rough Frank-ordered weighted geometric (CFRFOWG) AOs

This idea of CFRFOWG AOs is elaborated here. Moreover, we have delivered the properties of these introduced ideas.

#### Definition 14

Let ${\hat{C} }_{om.-j}=\left({\mathfrak{L}}_{j}^{{\rotatebox{180}{{\rm h}}}^{\diamond }}+\iota { \frown\!\!\!\!\!\! {\smallint} }_{j}^{{\rotatebox{180}{{\rm h}}}^{\diamond }}, {\mathfrak{L}}_{j}^{{ { \bar{\d{\rm L}}} }^{\diamond }}+\iota { \frown\!\!\!\!\!\! {\smallint} }_{j}^{{ { \bar{\d{\rm L}}} }^{\diamond }}\right) \left(j=1, 2, .., n\right)$ be a collection of CFRNs, then

7 $$CFRFOWG\left({\hat{\text{C}} }_{om.-1}, {\hat{\text{C}} }_{om.-2}, \ldots , {\hat{\text{C}} }_{om.-n}\right)=\begin{array}{c}n\\ \otimes \\ j=1\end{array}{\left({\hat{\text{C}} }_{om.-\mathfrak{d}\left(j\right)} \right)}^{{\Omega }_{{\mathbbm{w}}-j}}$$

where, $\left(\mathfrak{d}\left(1\right),\mathfrak{d}\left(2\right),\dots ,\mathfrak{d}\left(n\right)\right)$ represent the permutation of $j=1, 2, .., n$ with ${\hat{C} }_{om.-\mathfrak{d}\left(j-1\right)}\ge {\hat{C} }_{om.-\mathfrak{d}\left(j\right)}$ for all $j.$

#### Theorem 5

*Let* ${\hat{C} }_{om.-j}=\left({\mathfrak{L}}_{j}^{{\rotatebox{180}{{\rm h}}}^{\diamond }}+\iota { \frown\!\!\!\!\!\! {\smallint} }_{j}^{{\rotatebox{180}{{\rm h}}}^{\diamond }}, {\mathfrak{L}}_{j}^{{ { \bar{\d{\rm L}}} }^{\diamond }}+\iota { \frown\!\!\!\!\!\! {\smallint} }_{j}^{{ { \bar{\d{\rm L}}} }^{\diamond }}\right) \left(j=1, 2, .., n\right)$ *be a collection of CFRNs, then the aggregated result by using* Eq. (7) *is again CFRN*

8 $$\begin{aligned} & CFRFOWG\left({\hat{C} }_{om.-1}, {\hat{C} }_{om.-2}, \dots , {\hat{C} }_{om.-n}\right)\\ & \quad =\left(\begin{array}{c}\left({{\text{log}}}_{{{{\sf (} \! \rotatebox{146}{\sf c}}}^{\divideontimes }}\left(1+\prod_{j=1}^{n}{\left({{{{\sf (} \! \rotatebox{146}{\sf c}}}^{\divideontimes }}^{{\mathfrak{L}}_{\mathfrak{d}\left(j\right)}^{{\rotatebox{180}{{\rm h}}}^{\diamond }}}-1\right)}^{{\Omega }_{{\mathbbm{w}}-j}}\right)\right)+\iota \left({{\text{log}}}_{{{{\sf (} \! \rotatebox{146}{\sf c}}}^{\divideontimes }}\left(1+\prod_{j=1}^{n}{\left({{{{\sf (} \! \rotatebox{146}{\sf c}}}^{\divideontimes }}^{{ \frown\!\!\!\!\!\! {\smallint} }_{\mathfrak{d}\left(j\right)}^{{\rotatebox{180}{{\rm h}}}^{\diamond }}}-1\right)}^{{\Omega }_{{\mathbbm{w}}-j}}\right)\right),\\ \left({{\text{log}}}_{{{{\sf (} \! \rotatebox{146}{\sf c}}}^{\divideontimes }}\left(1+\prod_{j=1}^{n}{\left({{{{\sf (} \! \rotatebox{146}{\sf c}}}^{\divideontimes }}^{{\mathfrak{L}}_{\mathfrak{d}\left(j\right)}^{{ { \bar{\d{\rm L}}} }^{\diamond }}}-1\right)}^{{\Omega }_{{\mathbbm{w}}-j}}\right)\right)+\iota \left({{\text{log}}}_{{{{\sf (} \! \rotatebox{146}{\sf c}}}^{\divideontimes }}\left(1+\prod_{j=1}^{n}{\left({{{{\sf (} \! \rotatebox{146}{\sf c}}}^{\divideontimes }}^{{ \frown\!\!\!\!\!\! {\smallint} }_{\mathfrak{d}\left(j\right)}^{{ { \bar{\d{\rm L}}} }^{\diamond }}}-1\right)}^{{\Omega }_{{\mathbbm{w}}-j}}\right)\right),\end{array}\right)\end{aligned}$$

#### Proof

Proof is the same as Theorem 2.

Let ${\hat{C} }_{om.-j}=\left({\mathfrak{L}}_{j}^{{\rotatebox{180}{{\rm h}}}^{\diamond }}+\iota { \frown\!\!\!\!\!\! {\smallint} }_{j}^{{\rotatebox{180}{{\rm h}}}^{\diamond }}, {\mathfrak{L}}_{j}^{{ { \bar{\d{\rm L}}} }^{\diamond }}+\iota { \frown\!\!\!\!\!\! {\smallint} }_{j}^{{ { \bar{\d{\rm L}}} }^{\diamond }}\right)$ and ${\hat{C} }_{om.-j}^{\divideontimes }=\left({{\mathfrak{L}}^{\divideontimes }}_{j}^{{\rotatebox{180}{{\rm h}}}^{\diamond }}+\iota { \frown\!\!\!\!\!\! {\smallint} \divideontimes }_{j}^{{\rotatebox{180}{{\rm h}}}^{\diamond }}, {{\mathfrak{L}}^{\divideontimes }}_{j}^{{ { \bar{\d{\rm L}}} }^{\diamond }}+\iota { \frown\!\!\!\!\!\! {\smallint} \divideontimes }_{j}^{{ { \bar{\d{\rm L}}} }^{\diamond }}\right) \left(j=1, 2, .., n\right)$ be two collections of CFRNs, then the following properties hold for CFRFWG AOs.

1. (Idempotency): If all ${\hat{C} }_{om.-j}={\hat{C} }_{om.} \forall j$ then

$$CFRFOWG\left({\hat{C} }_{om.-1}, {\hat{C} }_{om.-2}, \dots , {\hat{C} }_{om.-n}\right)={\hat{C} }_{om.}$$

2. Boundedness: Let ${{\hat{\text{C}} }_{om.}}^{-}=\left(\underset{j}{{\text{min}}}\left\{{\mathfrak{L}}_{j}^{{{{\rotatebox{180}{{\rm h}}}}}{\diamond }}\right\}+\iota \underset{j}{{\text{min}}}\left\{{ \frown\!\!\!\!\!\! {\smallint} }_{j}^{{{{\rotatebox{180}{{\rm h}}}}}{\diamond }}\right\}, \underset{j}{{\text{min}}}\left\{{\mathfrak{L}}_{j}^{{{{ { \bar{\d{\rm L}}} }}}{\diamond }}\right\}+\iota \underset{j}{{\text{min}}}\left\{{ \frown\!\!\!\!\!\! {\smallint} }_{j}^{{{{ { \bar{\d{\rm L}}} }}}{\diamond }}\right\}\right),$ and ${{\hat{\text{C}} }_{om.}}^{+}=\left(\underset{j}{{\text{max}}}\left\{{\mathfrak{L}}_{j}^{{{{\rotatebox{180}{{\rm h}}}}}{\diamond }}\right\}+\iota \underset{j}{{\text{max}}}\left\{{ \frown\!\!\!\!\!\! {\smallint} }_{j}^{{{{\rotatebox{180}{{\rm h}}}}}{\diamond }}\right\}, \underset{j}{{\text{max}}}\left\{{\mathfrak{L}}_{j}^{{{{ { \bar{\d{\rm L}}} }}}{\diamond }}\right\}+\iota \underset{j}{{\text{max}}}\left\{{ \frown\!\!\!\!\!\! {\smallint} }_{j}^{{{{ { \bar{\d{\rm L}}} }}}{\diamond }}\right\}\right)$. Then

$${{\hat{C} }_{om.}}^{-}\le CFRFOWG\left({\hat{C} }_{om.-1}, {\hat{C} }_{om.-2}, \dots , {\hat{C} }_{om.-n}\right)\le {{\hat{C} }_{om.}}^{+}$$

3. Monotonicity: If ${\mathfrak{L}}_{j}^{{\rotatebox{180}{{\rm h}}}^{\diamond }}\le {{\mathfrak{L}}^{\divideontimes }}_{j}^{{\rotatebox{180}{{\rm h}}}^{\diamond }}$, ${ \frown\!\!\!\!\!\! {\smallint} }_{j}^{{\rotatebox{180}{{\rm h}}}^{\diamond }}\le { \frown\!\!\!\!\!\! {\smallint} \divideontimes }_{j}^{{\rotatebox{180}{{\rm h}}}^{\diamond }}$, ${\mathfrak{L}}_{j}^{{ { \bar{\d{\rm L}}} }^{\diamond }}\le {{\mathfrak{L}}^{\divideontimes }}_{j}^{{ { \bar{\d{\rm L}}} }^{\diamond }}$, ${ \frown\!\!\!\!\!\! {\smallint} }_{j}^{{ { \bar{\d{\rm L}}} }^{\diamond }}\le { \frown\!\!\!\!\!\! {\smallint} \divideontimes }_{j}^{{ { \bar{\d{\rm L}}} }^{\diamond }}\forall j$, then

$$CFRFOWG\left({\hat{C} }_{om.-1}, {\hat{C} }_{om.-2}, \dots , {\hat{C} }_{om.-n}\right)\le CFRFOWG\left({\hat{C} }_{om.-1}^{\divideontimes }, {\hat{C} }_{om.-2}^{\divideontimes }, \dots , {\hat{C} }_{om.-n}^{\divideontimes }\right)$$

### Complex fuzzy rough Frank hybrid weighted geometric (CFRHWG) AOs

By combining the characteristics of the CFRFWG and CFRFOWG, we have developed the structure of CFRFHWG AOs.

#### Definition 15

 Let ${\hat{C} }_{om.-j}=\left({\mathfrak{L}}_{j}^{{\rotatebox{180}{{\rm h}}}^{\diamond }}+\iota { \frown\!\!\!\!\!\! {\smallint} }_{j}^{{\rotatebox{180}{{\rm h}}}^{\diamond }}, {\mathfrak{L}}_{j}^{{ { \bar{\d{\rm L}}} }^{\diamond }}+\iota { \frown\!\!\!\!\!\! {\smallint} }_{j}^{{ { \bar{\d{\rm L}}} }^{\diamond }}\right) \left(j=1, 2, .., n\right)$ be a collection of CFRNs, then CFRHWG AOs are given by

$$CFRFHWG\left({\hat{C} }_{om.-1}, {\hat{C} }_{om.-2}, \dots , {\hat{C} }_{om.-n}\right)=\begin{array}{c}n\\ \otimes \\ j=1\end{array}{\left({{\hat{C} }^{\between }}_{om.-\mathfrak{d}\left(j\right)}\right)}^{{{\Omega }^{\between }}_{{\mathbbm{w}}-j}}$$

$$=\left(\begin{array}{c}{{\text{log}}}_{{{{\sf (} \! \rotatebox{146}{\sf c}}}^{\divideontimes }}\left(1+\prod_{j=1}^{n}{\left({{{{\sf (} \! \rotatebox{146}{\sf c}}}^{\divideontimes }}^{{\mathfrak{L}}_{\mathfrak{d}\left(j\right)}^{{\rotatebox{180}{{\rm h}}}^{\diamond }}}-1\right)}^{{\Omega }_{{\mathbbm{w}}-j}}\right)+\iota {{\text{log}}}_{{{{\sf (} \! \rotatebox{146}{\sf c}}}^{\divideontimes }}\left(1+\prod_{j=1}^{n}{\left({{{{\sf (} \! \rotatebox{146}{\sf c}}}^{\divideontimes }}^{{ \frown\!\!\!\!\!\! {\smallint} }_{\mathfrak{d}\left(j\right)}^{{\rotatebox{180}{{\rm h}}}^{\diamond }}}-1\right)}^{{\Omega }_{{\mathbbm{w}}-j}}\right),\\ {{\text{log}}}_{{{{\sf (} \! \rotatebox{146}{\sf c}}}^{\divideontimes }}\left(1+\prod_{j=1}^{n}{\left({{{{\sf (} \! \rotatebox{146}{\sf c}}}^{\divideontimes }}^{{\mathfrak{L}}_{\mathfrak{d}\left(j\right)}^{{ { \bar{\d{\rm L}}} }^{\diamond }}}-1\right)}^{{\Omega }_{{\mathbbm{w}}-j}}\right)+\iota 1-{{\text{log}}}_{{{{\sf (} \! \rotatebox{146}{\sf c}}}^{\divideontimes }}\left(1+\prod_{j=1}^{n}{\left({{{{\sf (} \! \rotatebox{146}{\sf c}}}^{\divideontimes }}^{{ \frown\!\!\!\!\!\! {\smallint} }_{\mathfrak{d}\left(j\right)}^{{ { \bar{\d{\rm L}}} }^{\diamond }}}-1\right)}^{{\Omega }_{{\mathbbm{w}}-j}}\right)\end{array}\right)$$

where ${\Omega }_{\mathbbm{w}}^{*}=\left({\Omega }_{{\mathbbm{w}}-1}^{*}, {\Omega }_{{\mathbbm{w}}-2}^{*}\dots , {\Omega }_{{\mathbbm{w}}-n}^{*}\right)$ is the linked WV such that $0\le {\Omega }_{{\mathbbm{w}}-j}^{*}\le 1$, and $\sum_{j=1}^{n}{\Omega }_{{\mathbbm{w}}-j}^{*}=1, {\Omega }_{\mathbbm{w}}=\left({\Omega }_{{\mathbbm{w}}-1}, {\Omega }_{{\mathbbm{w}}-2}\dots , {\Omega }_{{\mathbbm{w}}-n}\right)$ is the WV of ${\hat{C} }_{om.-j}\left(j=1, 2, \dots , n\right)$ such that $0\le {\Omega }_{{\mathbbm{w}}-j}\le 1$, and $\sum_{j=1}^{n}{\Omega }_{{\mathbbm{w}}-j}=1$ is the $jth$ major weighted CFR values of ${\hat{C} }_{om.-j}^{*}\left({\hat{C} }_{om.-j}^{*}=n{\Omega }_{{\mathbbm{w}}-j}{\hat{C} }_{om.-j}\right), n$ is the balancing coefficient.

Let ${\hat{C} }_{om.-j}=\left({\mathfrak{L}}_{j}^{{\rotatebox{180}{{\rm h}}}^{\diamond }}+\iota { \frown\!\!\!\!\!\! {\smallint} }_{j}^{{\rotatebox{180}{{\rm h}}}^{\diamond }}, {\mathfrak{L}}_{j}^{{ { \bar{\d{\rm L}}} }^{\diamond }}+\iota { \frown\!\!\!\!\!\! {\smallint} }_{j}^{{ { \bar{\d{\rm L}}} }^{\diamond }}\right)$ and ${\hat{C} }_{om.-j}^{\divideontimes }=\left({{\mathfrak{L}}^{\divideontimes }}_{j}^{{\rotatebox{180}{{\rm h}}}^{\diamond }}+\iota { \frown\!\!\!\!\!\! {\smallint} \divideontimes }_{j}^{{\rotatebox{180}{{\rm h}}}^{\diamond }}, {{\mathfrak{L}}^{\divideontimes }}_{j}^{{ { \bar{\d{\rm L}}} }^{\diamond }}+\iota { \frown\!\!\!\!\!\! {\smallint} \divideontimes }_{j}^{{ { \bar{\d{\rm L}}} }^{\diamond }}\right) \left(j=1, 2, .., n\right)$ be two collections of CFRNs, then the following characteristics hold for CFRFHWG AOs.

1. (Idempotency): If all ${\hat{C} }_{om.-j}={\hat{C} }_{om.} \forall j$ then

$$CFRFHWG\left({\hat{C} }_{om.-1}, {\hat{C} }_{om.-2}, \dots , {\hat{C} }_{om.-n}\right)={\hat{C} }_{om.}$$

2. Boundedness: Let ${{\hat{\text{C}} }_{om.}}^{-}=\left(\underset{j}{{\text{min}}}\left\{{\mathfrak{L}}_{j}^{{{{\rotatebox{180}{{\rm h}}}}}{\diamond }}\right\}+\iota \underset{j}{{\text{min}}}\left\{{ \frown\!\!\!\!\!\! {\smallint} }_{j}^{{{{\rotatebox{180}{{\rm h}}}}}{\diamond }}\right\}, \underset{j}{{\text{min}}}\left\{{\mathfrak{L}}_{j}^{{{{ { \bar{\d{\rm L}}} }}}{\diamond }}\right\}+\iota \underset{j}{{\text{min}}}\left\{{ \frown\!\!\!\!\!\! {\smallint} }_{j}^{{{{ { \bar{\d{\rm L}}} }}}{\diamond }}\right\}\right),$ and ${{\hat{\text{C}} }_{om.}}^{+}=\left(\underset{j}{{\text{max}}}\left\{{\mathfrak{L}}_{j}^{{{{\rotatebox{180}{{\rm h}}}}}{\diamond }}\right\}+\iota \underset{j}{{\text{max}}}\left\{{ \frown\!\!\!\!\!\! {\smallint} }_{j}^{{{{\rotatebox{180}{{\rm h}}}}}{\diamond }}\right\}, \underset{j}{{\text{max}}}\left\{{\mathfrak{L}}_{j}^{{{{ { \bar{\d{\rm L}}} }}}{\diamond }}\right\}+\iota \underset{j}{{\text{max}}}\left\{{ \frown\!\!\!\!\!\! {\smallint} }_{j}^{{{{ { \bar{\d{\rm L}}} }}}{\diamond }}\right\}\right)$. Then

$${{\hat{C} }_{om.}}^{-}\le CFRFHWG\left({\hat{C} }_{om.-1}, {\hat{C} }_{om.-2}, \dots , {\hat{C} }_{om.-n}\right)\le {{\hat{C} }_{om.}}^{+}$$

3. Monotonicity: If ${\mathfrak{L}}_{j}^{{\rotatebox{180}{{\rm h}}}^{\diamond }}\le {{\mathfrak{L}}^{\divideontimes }}_{j}^{{\rotatebox{180}{{\rm h}}}^{\diamond }}$, ${ \frown\!\!\!\!\!\! {\smallint} }_{j}^{{\rotatebox{180}{{\rm h}}}^{\diamond }}\le { \frown\!\!\!\!\!\! {\smallint} \divideontimes }_{j}^{{\rotatebox{180}{{\rm h}}}^{\diamond }}$, ${\mathfrak{L}}_{j}^{{ { \bar{\d{\rm L}}} }^{\diamond }}\le {{\mathfrak{L}}^{\divideontimes }}_{j}^{{ { \bar{\d{\rm L}}} }^{\diamond }}$, ${ \frown\!\!\!\!\!\! {\smallint} }_{j}^{{ { \bar{\d{\rm L}}} }^{\diamond }}\le { \frown\!\!\!\!\!\! {\smallint} \divideontimes }_{j}^{{ { \bar{\d{\rm L}}} }^{\diamond }}\forall j$, then

$$CFRFHWG\left({\hat{C} }_{om.-1}, {\hat{C} }_{om.-2}, \dots , {\hat{C} }_{om.-n}\right)\le CFRFHWG\left({\hat{C} }_{om.-1}^{\divideontimes }, {\hat{C} }_{om.-2}^{\divideontimes }, \dots , {\hat{C} }_{om.-n}^{\divideontimes }\right)$$

#### Remark 2

1. By using ${\Omega }_{\mathbbm{w}}={\left(\frac{1}{n}, \frac{1}{n}, \dots , \frac{1}{n}\right)}^{t}$ we have ${\hat{C} }_{om.-j}^{\divideontimes }=n\times \frac{1}{n}\times {\hat{C} }_{om.-j}={\hat{C} }_{om.-j}$ for $j=1, 2, .., n$. Hence, CFRFHWG AOs degenerate into CFRFOWG AOs.
2. By utilizing ${\Omega }_{{\mathbbm{w}}-j}^{*}={\left(\frac{1}{n}, \frac{1}{n}, \dots , \frac{1}{n}\right)}^{t}$, the CFRFWHG AOs degenerate into CFRFWG AOs.

## MADM approach for the developed notions

To see the effective use of the developed approach, here in this part of the article, we have to discuss the multi-attribute decision-making approach for the utilization of delivered notions. The MADM approach is one of the famous approaches for decision-making scenarios. In this approach, a specific algorithm based on developed theory is utilized for the selection of suitable alternatives from the set of given alternatives. Now we have to discuss that algorithm as follows:

Assume the set ${\mathfrak{G}}_{ter.}=\left\{{\mathfrak{G}}_{ter.-1}, {\mathfrak{G}}_{ter.-2}, {\mathfrak{G}}_{ter.-3}, \dots , {\mathfrak{G}}_{ter.-m}\right\}$ represent the alternatives set and there are $"m"$ alternatives. Furthermore, the set of $"n"$ attributes is given by ${\mathfrak{S}}_{atr.}=\left\{{\mathfrak{S}}_{atr.-1}, {\mathfrak{S}}_{atr.-2}, {\mathfrak{S}}_{atr.-3}, \dots , {\mathfrak{S}}_{atr.-n}\right\}.$ Let ${\Omega }_{\mathbbm{w}}=\left({\Omega }_{{\mathbbm{w}}-1}, {\Omega }_{{\mathbbm{w}}-2}, {\Omega }_{{\mathbbm{w}}-3}, \dots , {\Omega }_{{\mathbbm{w}}-n}\right)$ be the WVs of attributes such that $0\le {\Omega }_{{\mathbbm{w}}-j}\le 1$, and $\sum_{j=1}^{n}{\Omega }_{{\mathbbm{w}}-j}=1.$ Suppose decision analyst provide their assessment in the form of CFRNs and these values are given in the form of a matrix as $\mathfrak{Y}={\left({\hat{C} }_{om.-ij}\right)}_{m\times n}={\left({\mathfrak{L}}_{ij}^{{\rotatebox{180}{{\rm h}}}^{\diamond }}+\iota { \frown\!\!\!\!\!\! {\smallint} }_{ij}^{{\rotatebox{180}{{\rm h}}}^{\diamond }}, {\mathfrak{L}}_{ij}^{{ { \bar{\d{\rm L}}} }^{\diamond }}+\iota { \frown\!\!\!\!\!\! {\smallint} }_{ij}^{{ { \bar{\d{\rm L}}} }^{\diamond }}\right)}_{m\times n}$ where $\left(i:1, 2,\dots , m\right)\, {\text{and}}\, \left(j:1, 2,\dots , n\right).$ Now the stepwise algorithm is given by:

### MADM algorithm

We have to deliver the MADM algorithm under the environment of delivered ideas of CFRFWA and CFRFWG AOs as follows:

Step 1: Utilize the developed ideas of CFRFWA and CFRFWG AOs to the CFR data given in matrix $\mathfrak{Y}={\left({\hat{C} }_{om.-ij}\right)}_{m\times n}$

$${\hat{C} }_{om.-i}= CFRFWA\left({\hat{C} }_{om.-i1}, {\hat{C} }_{om.-i2}, {\hat{C} }_{om.-i3}, \dots , {\hat{C} }_{om.-in}\right)=\begin{array}{c}n\\ \oplus \\ j=1\end{array}{\Omega }_{{\mathbbm{w}}-j}{\hat{C} }_{om.-ij}$$

$$=\left(\begin{array}{c}1-\left({{\text{log}}}_{{{{\sf (} \! \rotatebox{146}{\sf c}}}^{\divideontimes }}\left(1+\prod_{j=1}^{n}{\left({{{{\sf (} \! \rotatebox{146}{\sf c}}}^{\divideontimes }}^{1-{\mathfrak{L}}_{ij}^{{\rotatebox{180}{{\rm h}}}^{\diamond }}}-1\right)}^{{\Omega }_{{\mathbbm{w}}-j}}\right)\right)+\iota 1-\left({{\text{log}}}_{{{{\sf (} \! \rotatebox{146}{\sf c}}}^{\divideontimes }}\left(1+\prod_{j=1}^{n}{\left({{{{\sf (} \! \rotatebox{146}{\sf c}}}^{\divideontimes }}^{1-{ \frown\!\!\!\!\!\! {\smallint} }_{ij}^{{\rotatebox{180}{{\rm h}}}^{\diamond }}}-1\right)}^{{\Omega }_{{\mathbbm{w}}-j}}\right)\right),\\ 1-\left({{\text{log}}}_{{{{\sf (} \! \rotatebox{146}{\sf c}}}^{\divideontimes }}\left(1+\prod_{j=1}^{n}{\left({{{{\sf (} \! \rotatebox{146}{\sf c}}}^{\divideontimes }}^{1-{\mathfrak{L}}_{ij}^{{ { \bar{\d{\rm L}}} }^{\diamond }}}-1\right)}^{{\Omega }_{{\mathbbm{w}}-j}}\right)\right)+\iota 1-\left({{\text{log}}}_{{{{\sf (} \! \rotatebox{146}{\sf c}}}^{\divideontimes }}\left(1+\prod_{j=1}^{n}{\left({{{{\sf (} \! \rotatebox{146}{\sf c}}}^{\divideontimes }}^{1-{ \frown\!\!\!\!\!\! {\smallint} }_{ij}^{{ { \bar{\d{\rm L}}} }^{\diamond }}}-1\right)}^{{\Omega }_{{\mathbbm{w}}-j}}\right)\right),\end{array}\right)$$

or

$${\hat{C} }_{om.-i}= CFRFWG\left({\hat{C} }_{om.-i1}, {\hat{C} }_{om.-i2}, {\hat{C} }_{om.-i3}, \dots , {\hat{C} }_{om.-in}\right)=\begin{array}{c}n\\ \otimes \\ j=1\end{array}{\left({\hat{C} }_{om.-j}\right)}^{{\Omega }_{{\mathbbm{w}}-j}}$$

$$=\left(\begin{array}{c}\left({{\text{log}}}_{{{{\sf (} \! \rotatebox{146}{\sf c}}}^{\divideontimes }}\left(1+\prod_{j=1}^{n}{\left({{{{\sf (} \! \rotatebox{146}{\sf c}}}^{\divideontimes }}^{{\mathfrak{L}}_{ij}^{{\rotatebox{180}{{\rm h}}}^{\diamond }}}-1\right)}^{{\Omega }_{{\mathbbm{w}}-j}}\right)\right)+\iota \left({{\text{log}}}_{{{{\sf (} \! \rotatebox{146}{\sf c}}}^{\divideontimes }}\left(1+\prod_{j=1}^{n}{\left({{{{\sf (} \! \rotatebox{146}{\sf c}}}^{\divideontimes }}^{{ \frown\!\!\!\!\!\! {\smallint} }_{ij}^{{\rotatebox{180}{{\rm h}}}^{\diamond }}}-1\right)}^{{\Omega }_{{\mathbbm{w}}-j}}\right)\right),\\ \left({{\text{log}}}_{{{{\sf (} \! \rotatebox{146}{\sf c}}}^{\divideontimes }}\left(1+\prod_{j=1}^{n}{\left({{{{\sf (} \! \rotatebox{146}{\sf c}}}^{\divideontimes }}^{{\mathfrak{L}}_{ij}^{{ { \bar{\d{\rm L}}} }^{\diamond }}}-1\right)}^{{\Omega }_{{\mathbbm{w}}-j}}\right)\right)+\iota \left({{\text{log}}}_{{{{\sf (} \! \rotatebox{146}{\sf c}}}^{\divideontimes }}\left(1+\prod_{j=1}^{n}{\left({{{{\sf (} \! \rotatebox{146}{\sf c}}}^{\divideontimes }}^{{ \frown\!\!\!\!\!\! {\smallint} }_{ij}^{{ { \bar{\d{\rm L}}} }^{\diamond }}}-1\right)}^{{\Omega }_{{\mathbbm{w}}-j}}\right)\right),\end{array}\right)$$

Step 2. Now use the Definition 9 to obtain the score values $Scr.\left({\hat{C} }_{om.-i}\right) \left(i=1, 2, 3, \dots , m\right)$ for ${\hat{C} }_{om.-i}$ to rank the alternatives. If the score value for any two CFRNs are the same then we utilize the accuracy function to rank the alternatives.

Step 3. Order all the alternatives to select the best alternative.

Step 4. End.

## Numerical example

### AI in civil engineering

The design, development, and maintenance of the physical and natural built environment are the focus of the engineering discipline known as civil engineering. It includes a broad range of tasks and initiatives, such as the design and construction of public works like highways, tunnels, bridges, airports, dams, buildings, and water and sewage systems. Civil engineers are in the position of assuring the sustainability, use, as well as security of these systems and structures, taking into account elements like cost-effectiveness, ecological impact, and choice of materials. They have a significant impact on how modern society is built and how its citizens' quality of life is maintained.

Numerous industries, including civil engineering, have adopted artificial intelligence (AI) in important ways. Infrastructural project planning, design, construction, and management practices for civil engineers are being revolutionized by IT. In civil engineering, the following are some important applications of AI (1) Design optimization (2) Structural health monitoring (3) Construction Management (4) environmental impact assessment (5) Risk assessment (6) Cost estimation (7) Building information modeling (8) Safety Monitoring (9) Natural disaster Preparedness (10) Project collaboration.

### Importance of AI in civil engineering

Here are some key points that show the importance of AI in civil engineering

1. Data analysis, design optimization, and project scheduling are just a few examples of the repetitive, time-consuming jobs that AI can automate. As a result, civil engineers can concentrate on more challenging and original areas of their work.
2. Large amounts of data from numerous sources, such as sensors and remote monitoring systems, can be processed by AI. It may analyze this data to offer perceptions, forecasts, and trends that can enhance planning, maintenance, and construction decision-making.
3. By performing simulations and optimizing designs depending on different characteristics, AI-powered tools can help with the design of structures and systems, resulting in more affordable and reliable solutions.
4. By examining past data and identifying prospective dangers, AI can assist in assessing the risks related to civil engineering projects. Engineers can reduce risks by making decisions based on this knowledge.
5. By analyzing sensor data, artificial intelligence (AI) can be used to continuously monitor the performance and health of infrastructure, such as bridges and buildings. It can spot early indications of damage and suggest upkeep or repairs, improving safety.
6. AI-powered project management solutions can help with planning, allocating resources, and scheduling to make sure that projects are finished on time and under budget.
7. Drones and sensors with AI capabilities can check and remotely monitor infrastructure, eliminating the need for manual inspections in hazardous or difficult-to-reach areas.

### AI tools for civil engineering

Different kinds of AI tools can be helpful for civil engineers. These are (1) Civil.AI (2) Autodesk generative design (3) Plaxis AI (4) Tekla Structural Designer. The discussion of these tools is given as follows:

#### 1. Civil.AI

An innovative newcomer, Civils.AI, is introducing an AI-powered platform that will revolutionize the dynamic field of civil engineering. This platform is a pioneer in its industry and one of the top AI tools for civil engineers, efficiently aiding activities related to infrastructural project design, construction, and maintenance. The AI-driven design tools offered by Civils.AI are among its most alluring features. Automation of essential processes including structural analysis, material selection, and cost estimation increases productivity and improves job quality. The design process is greatly simplified by these AI-powered tools, increasing output.

#### 2. Autodesk

A well-known software company that specializes in creating CAD, 3D modeling, and engineering tools is Autodesk. The company's software solutions are extensively used across a range of sectors, including media, entertainment, manufacturing, engineering, and architecture. The following are some of the software applications and platforms offered by Autodesk (1) AutoCAD (2) Autodesk Maya (3) AutoCAD civil CD (4) AutoCAD LT etc.

Professionals from all over the world use Autodesk's tools to design, model, simulate, and visualize a variety of projects.

#### 3. Plaxis 3D

The computer programed PLAXIS does finite element analysis for deformation, stability, and water flow in the field of geotechnical engineering. The words "Plastic" and "AXI Symmetry," which refer to the geometric types handled in the software's original code, are combined to form the title PLAXIS. For the investigation and creation of three-dimensional (3D) subsurface models, geotechnical engineers utilize the software package Plaxis 3D. It is frequently used in geotechnical engineering projects, such as foundations, tunnels, retaining walls, embankments, and more, to simulate and analyze the behavior of soil, rock, and structures. Plaxis 3D's main attributes and features are listed as (1) 3D modeling (2) finite element analysis (3) Material Model (4) Retaining wall analysis (5) Slope stability.

#### 4. Tekla Structural Designer

A specialized piece of software called Tekla Structural Designer is employed in the building and structural engineering industries. It was created by Trimble Solutions Corporation to make it easier to analyze and create different structural systems. Static and dynamic analysis, load combinations, and response spectrum analysis are among the structural analysis techniques offered by this software. Complex structures can be modeled by engineers, and their behavior can be assessed under various stress scenarios. Tekla Structural Designer enables automated construction design optimization to save material consumption while maintaining safety and code compliance. Both money and the environment may benefit from this. Engineers can design and manage 3D models of structures since they support BIM workflows. Modern structural engineering projects frequently include integration with other BIM software and collaborative technologies, which is an essential component.

Assume that

$${\mathfrak{G}}_{\text{ter.-1}}={\text{Civil}}.\text{AI},$$

$${\mathfrak{G}}_{\text{ter.-2}}={\text{Autodesk}},$$

$${\mathfrak{G}}_{\text{ter.-3}}={\text{Plaxis}}\,3{\text{D}}$$

$${\mathfrak{G}}_{\text{ter.-4}}={\text{Tekla structure designer}}$$

are four alternatives and the attributes of these alternatives are ${\mathfrak{S}}_{\text{atr.-1}}={\text{Interactive modeling}},$

$${\mathfrak{S}}_{\text{atr.-2}}={\text{Automated structural analysis}},$$

$${\mathfrak{S}}_{\text{atr.-3}}=Drawing \, and \, report \, creation$$

$${\mathfrak{S}}_{\text{atr.-4}}={\text{Deformation and stability analysis}}$$

Now we aim to classify the alternatives based on the observation of these attributes. Assume that decision analyst pride their assessment in the form of CFRNs and overall data is given in Table 2.

**Table 2:** The complex fuzzy rough information.

	${\mathfrak{S}}_{{\varvec{a}}{\varvec{t}}{\varvec{r}}.-1}$	${\mathfrak{S}}_{{\varvec{a}}{\varvec{t}}{\varvec{r}}.-2}$	${\mathfrak{S}}_{{\varvec{a}}{\varvec{t}}{\varvec{r}}.-3}$	${\mathfrak{S}}_{{\varvec{a}}{\varvec{t}}{\varvec{r}}.-4}$
${\mathfrak{G}}_{{\varvec{t}}{\varvec{e}}{\varvec{r}}.-1}$	$$\left(\begin{array}{c}0.14+i 0.12, \\ 0.11+i 0.29\end{array}\right)$$	$$\left(\begin{array}{c}0.33+i 0.39, \\ 0.30+i 0.42\end{array}\right)$$	$$\left(\begin{array}{c}0.40+i 0.47, \\ 0.33+i 0.21\end{array}\right)$$	$$\left(\begin{array}{c}0.16+i 0.13, \\ 0.18+i 0.11\end{array}\right)$$
${\mathfrak{G}}_{{\varvec{t}}{\varvec{e}}{\varvec{r}}.-2}$	$$\left(\begin{array}{c}0.19+i 0.15, \\ 0.37+i 0.46\end{array}\right)$$	$$\left(\begin{array}{c}0.37+i 0.51, \\ 0.13+i 0.34\end{array}\right)$$	$$\left(\begin{array}{c}0.19+i 0.15, \\ 0.15+i 0.17\end{array}\right)$$	$$\left(\begin{array}{c}0.14+i 0.15, \\ 0.17+i 0.12\end{array}\right)$$
${\mathfrak{G}}_{{\varvec{t}}{\varvec{e}}{\varvec{r}}.-3}$	$$\left(\begin{array}{c}0.24+i 0.29, \\ 0.29+i 0.30\end{array}\right)$$	$$\left(\begin{array}{c}0.27+i 0.25, \\ 0.29+i 0.35\end{array}\right)$$	$$\left(\begin{array}{c}0.19+i 0.52, \\ 0.63+i 0.66\end{array}\right)$$	$$\left(\begin{array}{c}0.36+i 0.18, \\ 0.27+i 0.18\end{array}\right)$$
${\mathfrak{G}}_{{\varvec{t}}{\varvec{e}}{\varvec{r}}.-4}$	$$\left(\begin{array}{c}0.51+i 0.55, \\ 0.66+i 0.14\end{array}\right)$$	$$\left(\begin{array}{c}0.26+i 0.23, \\ 0.25+i 0.15\end{array}\right)$$	$$\left(\begin{array}{c}0.58+i 0.59, \\ 0.29+i 0.56\end{array}\right)$$	$$\left(\begin{array}{c}0.25+i 0.28, \\ 0.36+i 0.40\end{array}\right)$$

Now we apply the stepwise algorithm to conclude the result.

### Utilization of CFRFWA AOs

First of all, we will use the notion of CFRFWA aggregation operator for the application of the proposed theory.

Step 1: Use ${{{\sf (} \! \rotatebox{146}{\sf c}}}^{\divideontimes }=2$ and utilize the developed idea of CFRFWA AOs for the values of ${\hat{C} }_{om.-i}.$ Assume that WVs for attributes are $\left(0.23, 0.22, 0.29, 0.26\right)$.

$${\hat{\text{C}} }_{om.-1}=\left(0.269+{\mathfrak{i}} 0.297, 0.238+{\mathfrak{i}} 0.255\right)$$, $${\hat{\text{C}} }_{om.-2}=\left(0.2200+{\mathfrak{i}} 0.2430, 0.2060+{\mathfrak{i}} 0.2710\right)$$,

$${\hat{\text{C}} }_{om.-3}=\left(0.2660+{\mathfrak{i}} 0.3310, 0.4030+{\mathfrak{i}} 0.4120\right)$$, $${\hat{\text{C}} }_{om.-4}=\left(0.4230+{\mathfrak{i}} 0.4390, 0.4050+{\mathfrak{i}} 0.3520\right)$$

Step 2: Calculate the score values $Scr.\left({\hat{C} }_{om.-i}\right)\left(i=1, {2,3}, 4\right)$ of the overall CFRNs ${\hat{C} }_{om.-i} \left(i=1, {2,3}, 4\right)$

$$Scr.\left({\hat{C} }_{om.-1}\right)=0.2650, Scr.\left({\hat{C} }_{om.-2}\right)=0.2350,$$

$$Scr.\left({\hat{C} }_{om.-3}\right)=0.3530, Scr.\left({\hat{C} }_{om.-4}\right)=0.4047$$

Step 3. According to the results obtained from step 2, we can order the alternatives and find out the best alternative.

Hence ordering is as follows

$${\mathfrak{G}}_{ter.-4}> {\mathfrak{G}}_{ter.-3}>{\mathfrak{G}}_{ter.-1}>{\mathfrak{G}}_{ter.-2}$$

So ${\mathfrak{G}}_{ter.-4}$ is the best alternative.

Step 4. End.

### Utilization of CFRFWG AOs

Here we will utilize the notion of CFRFWG aggregation operator for the data given in Table 2.

Step 1: Use ${{{\sf (} \! \rotatebox{146}{\sf c}}}^{\divideontimes }=2$ and utilize the developed idea of CFRFWG AOs for the values of ${\hat{C} }_{om.-i}.$ Assume that WVs for attributes are $\left(0.23, 0.22, 0.29, 0.26\right)$.

$${\hat{\text{C}} }_{om.-1}=\left(0.2390+{\mathfrak{i}} 0.2400, 0.2160+ {\mathfrak{i}} 0.2250\right)$$, $${\hat{\text{C}} }_{om.-2}=\left(0.2040+{\mathfrak{i}} 0.1990, 0.1860+{\mathfrak{i}} 0.2300\right)$$,

$${\hat{\text{C}} }_{om.-3}=\left(0.256+{\mathfrak{i}} 0.296,0.3600+{\mathfrak{i}} 0.3460\right)$$, $${\hat{\text{C}} }_{om.-4}=\left(0.383+{\mathfrak{i}} 0.393, 0.3620+{\mathfrak{i}} 0.2840\right)$$

Step 2: Calculate the score values $Scr.\left({\hat{C} }_{om.-i}\right)\left(i=1, {2,3}, 4\right)$ of the overall CFRNs ${\hat{C} }_{om.-i} \left(i=1, {2,3}, 4\right)$

$$Scr.\left({\hat{C} }_{om.-1}\right)=0.2300, Scr.\left({\hat{C} }_{om.-2}\right)=0.2047,$$

$$Scr.\left({\hat{C} }_{om.-3}\right)=0.3145, Scr.\left({\hat{C} }_{om.-4}\right)=0.3555$$

Step 3: According to the results obtained in step 2, we can order the alternatives and find out the best alternative.

Hence ordering is as follows

$${\mathfrak{G}}_{ter.-4}> {\mathfrak{G}}_{ter.-3}>{\mathfrak{G}}_{ter.-1}>{\mathfrak{G}}_{ter.-2}$$

So ${\mathfrak{G}}_{ter.-4}$ is the best alternative.

Step 4. End.

### Comparative analysis

In this section, we will discuss the comparative study of the developed approach with some existing notions to prove the quality of the developed work. Here we will prove that our work is dominant to existing notions. We will compare our work with Zadeh's^9^ method, Dubois and Prade's^27^ method, Ramot et al.^18^ method, and Tamir et al.^19^ method. In this overall discussion, we will discuss the data given in Table 2.

1. A fuzzy set^9^ uses the membership grade and its value lies between [0, 1]. But if we have to discuss the second dimension in one structure then the idea of a fuzzy set fails to handle such kind of information. Just like the idea of a complex fuzzy rough set in the Cartesian form proposed in the developed theory can handle the second dimension and this developed structure has some extra features of considering the upper and lower approximations. This feature ranks this structure dominant to a fuzzy set and more complex data can be handled.
2. If we compare our work with Dubois and Prade^27^, then we can see that although the fuzzy rough set introduced by Dubois and Prade's^27^ method can cover the lower and upper approximation properties but there is a drawback of not considering the second dimension as we have covered this drawback in the introduced structure. So developed theory is more generalized.
3. Note that the idea of a complex fuzzy set delivered by Ramot et al.^18^ uses the range of membership grade as a unit circle in a complex plane, but this structure is less applicable than that of the developed theory because the introduced structure is more generalized form and it uses the range of membership grade in the unit square in the complex plane. Also, the delivered approach can consider the lower and upper approximations that property ranks the introduced approach more dominant to existing notions.
4. Note that in the Tamir et al.^19^ method, the notion of a complex fuzzy set is given in the Cartesian form. It is a more generalized form than that of the Ramot et al.^18^ method because it uses the unit square as a range set in the complex plane instead of the unit circle as discussed in Ramot et al.^18^ theory. Notice that although Tamir et al.^19^ have discussed the second dimension this structure has the drawback of not considering the lower and upper approximations as we have discussed in the developed theory. Moreover, in Tamir et al.^19^ structure, there is a chance of data loss but in the developed theory due to the generalization of fuzzy sets and complex fuzzy sets, there is no chance of data loss.
5. If we discuss the data-wise comparison then we can see that all the above existing approaches cannot consider the data given in Table 2, because the data given in Table 2 is based on complex fuzzy rough information. It means that the delivered approach is dominant to all existing approaches by all means.

**Figure 2:**
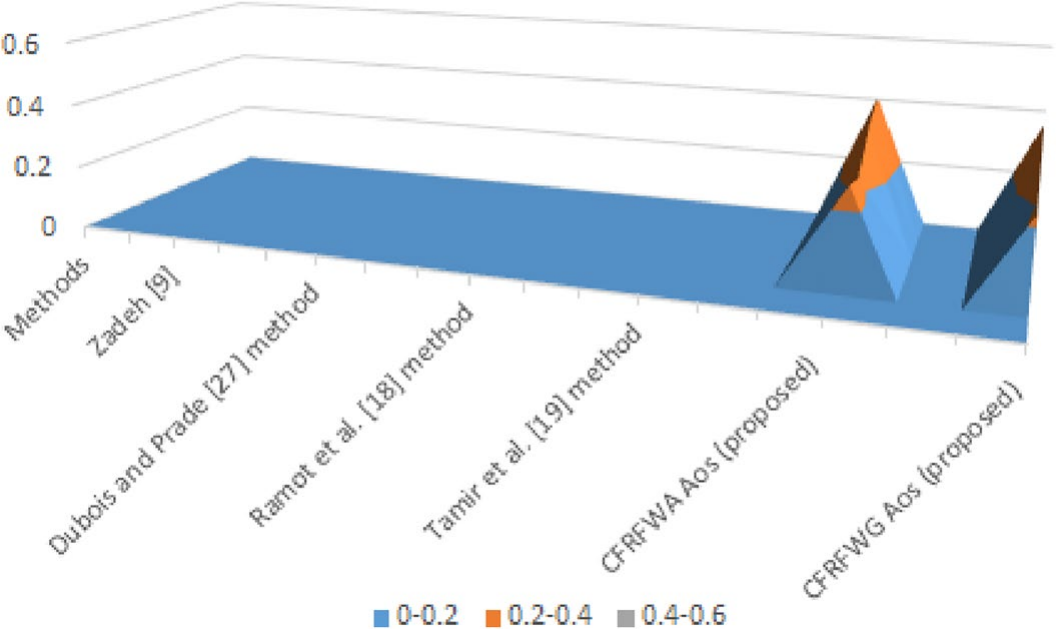
Graphical representation of data given in Table 3.

Moreover, the overall results are given in Table 3 and the graphical representation of the data present in Table 3 is given in Figure 2.

**Table 3:** Overall results.

Methods	Score values	Ranking results
Zadeh^9^	Cannot handle	No result
Dubois and Prade^27^ method	Cannot handle	No result
Ramot et al.^18^ method	Cannot handle	No result
Tamir et al.^19^ method	Cannot handle	No result
CFRFWA aggregation operators (proposed)		
CFRFWG aggregation operators (proposed)	$Scor.\left({\hat{C} }_{om.-1}\right)=0.2300, \,$ $Scor.\left({\hat{C} }_{om.-2}\right)=0.2047,\,$ $Scor.\left({\hat{C} }_{om.-3}\right)=0.3145, \,$ $Scor.\left({\hat{C} }_{om.-4}\right)=0.3555$	${\mathfrak{G}}_{ter.-4}> {\mathfrak{G}}_{ter.-3}>{\mathfrak{G}}_{ter.-1}>{\mathfrak{G}}_{ter.-2}$

## Conclusion

Researchers these days strive to handle as much data as possible and employ techniques and approaches that minimize or eliminate the possibility of data loss. The discussion of upper and lower approximations is not possible in structures such as fuzzy sets and complex fussy sets. Furthermore, we can see that the fuzzy rough set is unable to address the second dimension and that there is a risk of data loss in this situation. The idea of a complex fuzzy rough set in Cartesian form is necessary to address all of the concerns raised in earlier proposals since it can address both upper and lower approximations as well as the second dimension. So in this paper, we have defined the notion of a complex fuzzy rough set. Since the delivered approach in this form uses the range of membership grade as a unit square instead of a unit circle in the complex plane this property makes it dominant to many existing notions. AOs are the mathematical structure that converts the overall information into a single value and they can help in many decision-making situations. So based on frank t-norm and t-conorm, we have further discussed the theory of frank AOs under the notion of a complex fuzzy rough set. For the utilization of the delivered approach, we have defined an algorithm that can help in decision-making situations. We have proposed an example for the classification of AI tools for civil engineering. Moreover, the comparative analysis of the delivered approach shows the dominance of the introduced structure.

The established work is limited because whenever the decision-makers provide their assessment in the form of the complex fuzzy rough intuitionistic fuzzy rough and their generalized structure then the developed approach can never cover that kind of information because the introduced notion can never discuss the non-membership grade in upper and lower approximations with two dimensions.

Moreover, we can extend these notions to complex intuitionistic fuzzy rough sets and complex Pythagorean fuzzy rough sets.

## Data Availability

The datasets used and/or analyzed during the current study are available from the corresponding author on reasonable request.

## Abbreviations

US: Universal set
AI: Artificial intelligence
AOs: Aggregation operators
FSs: Fuzzy sets
RSs: Rough sets
FRSs: Fuzzy rough sets
IFRS: Intuitionistic fuzzy rough sets
PyFRSs: Pythagorean fuzzy rough sets
q-ROFRSs: Q-rung orthopair fuzzy rough sets
CFS: Complex fuzzy set
CFR: Complex fuzzy relation
CFRS: Complex fuzzy rough set
CFRNs: Complex fuzzy rough numbers
CFRFWA: Complex fuzzy rough Frank weighted average
CFRFOWA: Complex fuzzy rough Frank ordered weighted average
CFRFHWA: Complex fuzzy rough Frank hybrid weighted average
CFRFWG: Complex fuzzy rough Frank weighted geometric
CFRFOWG: Complex fuzzy rough Frank ordered weighted geometric
CFRFHWG: Complex fuzzy rough Frank hybrid weighted geometric
MADM: Multi-attribute decision making
